# The dynamics of genome replication using deep sequencing

**DOI:** 10.1093/nar/gkt878

**Published:** 2013-10-01

**Authors:** Carolin A. Müller, Michelle Hawkins, Renata Retkute, Sunir Malla, Ray Wilson, Martin J. Blythe, Ryuichiro Nakato, Makiko Komata, Katsuhiko Shirahige, Alessandro P.S. de Moura, Conrad A. Nieduszynski

**Affiliations:** ^1^School of Life Sciences, The University of Nottingham, Queen’s Medical Centre, Nottingham NG7 2UH, UK, ^2^Deep Seq, The University of Nottingham, Queen’s Medical Centre, Nottingham NG7 2UH, UK, ^3^Research Center for Epigenetic Disease, Institute of Molecular and Cellular Biosciences, The University of Tokyo, Tokyo, 113-0032, Japan and ^4^Institute for Complex Systems and Mathematical Biology, The University of Aberdeen, Aberdeen, AB24 3UE UK

## Abstract

Eukaryotic genomes are replicated from multiple DNA replication origins. We present complementary deep sequencing approaches to measure origin location and activity in *Saccharomyces cerevisiae*. Measuring the increase in DNA copy number during a synchronous S-phase allowed the precise determination of genome replication. To map origin locations, replication forks were stalled close to their initiation sites; therefore, copy number enrichment was limited to origins. Replication timing profiles were generated from asynchronous cultures using fluorescence-activated cell sorting. Applying this technique we show that the replication profiles of haploid and diploid cells are indistinguishable, indicating that both cell types use the same cohort of origins with the same activities. Finally, increasing sequencing depth allowed the direct measure of replication dynamics from an exponentially growing culture. This is the first time this approach, called marker frequency analysis, has been successfully applied to a eukaryote. These data provide a high-resolution resource and methodological framework for studying genome biology.

## INTRODUCTION

Complete replication of the genome is a requirement for successful cell division and therefore is fundamental to all life. The efficient replication of eukaryotic genomes requires coordinated initiation from thousands of DNA replication origins (1,2). Such coordinated origin activation leads to stereotypic replication-timing programs in which replication timing correlates with chromatin structure, gene expression, genome evolution and other critical aspects of genome metabolism. A major challenge in replication biology is the mapping of replication timing programs and the study of how they are regulated (3).

Individual origins vary in their time of activation during S-phase, creating a distinct spatial and temporal pattern of genome replication. Differences in origin activation time may reflect differences in affinity for a limited number of activating molecules, modulated by subnuclear positioning and chromatin state (4). In yeast, several mutations have been characterized that disrupt the temporal order of genome replication. Some of these mutants globally alter replication timing, either by changes to chromatin modifications or subnuclear positioning (5,6). Other mutations only alter the replication time of specific chromosomal domains. To date, mutants have been isolated that change the replication time of telomeres from late to early S-phase (7,8) and different mutants that delay the normally early replication time of centromeres (9).

Characterizing genome replication requires methodologies to locate replication origins and determine their activation time (10). Origins have been localized genome-wide by a range of approaches, including chromatin immunoprecipitation (ChIP) of ORC, MCM or incorporated BrdU, origin ‘bubble-trap’ assays, sequencing of Okazaki fragments, plasmid-based assays, measuring abundance of small nascent strands and replication in hydroxyurea (HU) (11–16). The temporal order of genome replication has been measured during a synchronous S-phase by tracking the progress of replication fork proteins, the incorporation of labeled nucleotides or direct detection of the products of DNA replication (17–19). Alternatively, cell sorting allows enrichment of replicating (and nonreplicating) cell populations without the requirement for cell cycle synchronization (20). Together these methods have allowed the measurement of genome replication dynamics in a range of mutants and various species, including *Saccharomyces cerevisiae*, *Schizosaccharomyces pombe* and cell lines from *Drosophila melanogaster*, *Mus musculus*, *Homo sapiens* and *Arabidopsis thaliana* (3). Although a variety of approaches have been used to map replication origins and measure replication kinetics, these techniques are often used in an *ad hoc* manner, and there have been few direct comparisons between them.

Here we present a direct comparison of different deep sequencing-based approaches for studying genome replication. First, we identified origin locations and activities using a checkpoint mutant subjected to the replication inhibitor HU. Second, a highly synchronous cell population was used to measure precisely wild-type replication dynamics. Third, replication dynamics in an asynchronous population were determined by sorting replicating cells. Finally, we show that replication dynamics can be directly measured from an exponentially growing cell population by direct sequencing of the genomic DNA. The use of a single strain background allows direct comparison between these approaches and provides a methodological and data resource for future investigation of genome replication.

## MATERIALS AND METHODS

### Yeast strains and methods

All strains used were from the W303 background and are listed in Supplementary Table S2. Cells were grown in standard rich YPD medium. For cell cycle synchronization, alpha factor was added to a final concentration of 200 nM; release was via resuspension in media containing 0.2 mg/ml pronase. Flow cytometry samples were fixed in 70% ethanol, washed with 50 mM sodium citrate, sonicated and treated with RNase A and proteinase K before staining with 1× SYTOX® green nucleic acid stain (Invitrogen). To kill culture samples for deep sequencing, sodium azide (final concentration 0.1%) and EDTA (20 mM) were added. Samples for marker frequency analysis (MFA) were grown at 30°C and harvested from exponential (OD_600_ of 0.7) and stationary phase (OD_600_ >4.0). RNaseA and proteinase K were used at final concentrations of 0.2 and 0.5 mg/ml, respectively, throughout. All DNA samples for deep sequencing were resuspended in TE (10 mM Tris, pH8, 1 mM EDTA).

### HU experiment

Cells were grown, arrested and released at 30°C into 200 mM HU. Cell pellets were resuspended in 5 ml cold freshly prepared NIB buffer (17% glycerol, 50 mM MOPS, 150 mM potassium acetate, 2 mM magnesium chloride, 500 mM spermidine, 150 mM spermine). After addition of a similar volume of glass beads, samples were vortexed vigorously for 30 s, followed by 30 s cooling in an ice-water bath. The vortex-cooling cycle was repeated until cell breakage was >95%. The extract was recovered from the glass beads and gently resuspended in 5 ml G2 buffer (QIAGEN). The sample was treated with RNase A and proteinase K followed by centrifugation. The supernatant was supplemented with 5 ml QBT buffer (QIAGEN) and then purified using an equilibrated QIAGEN Genomic-Tip 100/G column according to manufacturers’ instructions.

### Time course experiment

Cells were grown, arrested and released at 23°C and samples were collected every 2.5 min for ﬂow cytometry analysis and 5 min for isolation of genomic DNA. Samples for deep sequencing were resuspended in 1.6 ml of lysis buffer (10 mM Tris, pH8, 1 mM EDTA, 100 mM sodium chloride, 1% sodium dodecyl sulphate (SDS), 2% Triton X-100) to which 1.6 ml of glass beads, 0.8 ml of phenol and 0.8 ml chloroform were added. The sample was vortexed for 2 min, then the aqueous phase was recovered and treated with proteinase K and RNase A. The DNA was recovered by ethanol precipitation.

### Sort-seq

Cells were grown at 30°C to an OD_600_ of 0.65–0.85. Cells were pelleted, washed twice with water and fixed in 70% ethanol. Fixed cells were pelleted, washed twice and resuspended in 50 mM sodium citrate, sonicated and treated with RNase A and proteinase K. Cells were pelleted and resuspended in 50 mM sodium citrate containing 10× SYTOX® green nucleic acid stain (Invitrogen). At least 30 million cells were sorted from a particular cell cycle stage using a MoFlo Sorter (Coulter Beckman). The fluorescence-activated cell sorting (FACS) machine was set up according to the manufacturer’s instructions. An argon laser (488 nm) was used to excite the SYTOX® green stained cells. Data acquired in the FL1 channel was gated to remove background noise, cell debris and doublets. The FL1 histogram plot was used to set the gates to trigger the sorting. These were optimized for the yeast strains and adjusted manually throughout the sorting process as required. G2 phase cells were selected as the nonreplicating control owing to their greater abundance compared with G1-phase cells. The purity of the sorted cell fractions was confirmed by flow cytometry. Sorted cells were spheroplasted with Zymolyase (final concentration of 1 mg/ml) and then treated with SDS, proteinase K and RNase A. DNA was purified by phenol chloroform extraction followed by ethanol precipitation.

### ChIP-seq

ChIP was performed against FLAG-tagged Mcm4 using an anti-FLAG monoclonal antibody as described previously (21,22).

### Replication profiles

To generate replication timing profiles, the ratio of uniquely mapped reads in the replicating samples to the nonreplicating samples was calculated. Custom Perl scripts (available upon request) were used to independently calculate this ratio for every 1 kb window. Windows where fewer than a quarter of the expected number (based on total read number and the genome size) of reads were mapped in either sample were excluded. Differences in absolute read number were corrected for to give an average copy number value across the genome of one.

For the assessment of precision, the number of reads obtained from the 90 min sample were compared with the G1 sample. For the HU, sort-seq and MFA data were empirically normalized to give a minimum value of 1. For the time course experiment, each time point was normalized to the bulk DNA content as measured by flow cytometry. In each case, it was confirmed that the maximum DNA content did not exceed two genome copies. Comparisons between data sets were performed using the R environment, and quoted correlations are Pearson’s coefficients.

To calculate Trep profiles from replication time course data, a curve F[t] = c + [(d − c)/(1 + Exp[b(t − e)])^f] was fitted for every 1 kb window. The time at which the DNA at a given chromosomal coordinate has been replicated in half the cells is Trep = e + ln((−2(c − d)/(3 − 2c))^1/f^ − 1)/b.

### Smoothing, peak calling and motif identification

Where appropriate, replicating timing data were smoothed using a Fourier transformation method. The original signal was Fourier-transformed, the highest-frequency components were removed and the inverse Fourier transform was applied to obtain the smoothed signal. To avoid artifacts, smoothing was not applied to regions of low data density or the end 5 kb of each chromosome. Peak calling within genome-wide data was performed using the ‘peakdet’ script for MATLAB (from ‘www.billauer.co.il/peakdet.html’). To identify the enrichment of a single sequence motif, we used the GIbbsMarkov with Significance ANalysis (GIMSAN) algorithm (23). Motif searches were confined to windows of 40, 500 and 1000 bp centered on confirmed ARS Consensus Sequence (ACS) (Supplementary Table S3), Mcm4 ChIP-seq peaks and HU peaks, respectively.

## RESULTS

### Using deep sequencing to measure genome replication dynamics

**Figure 1.**
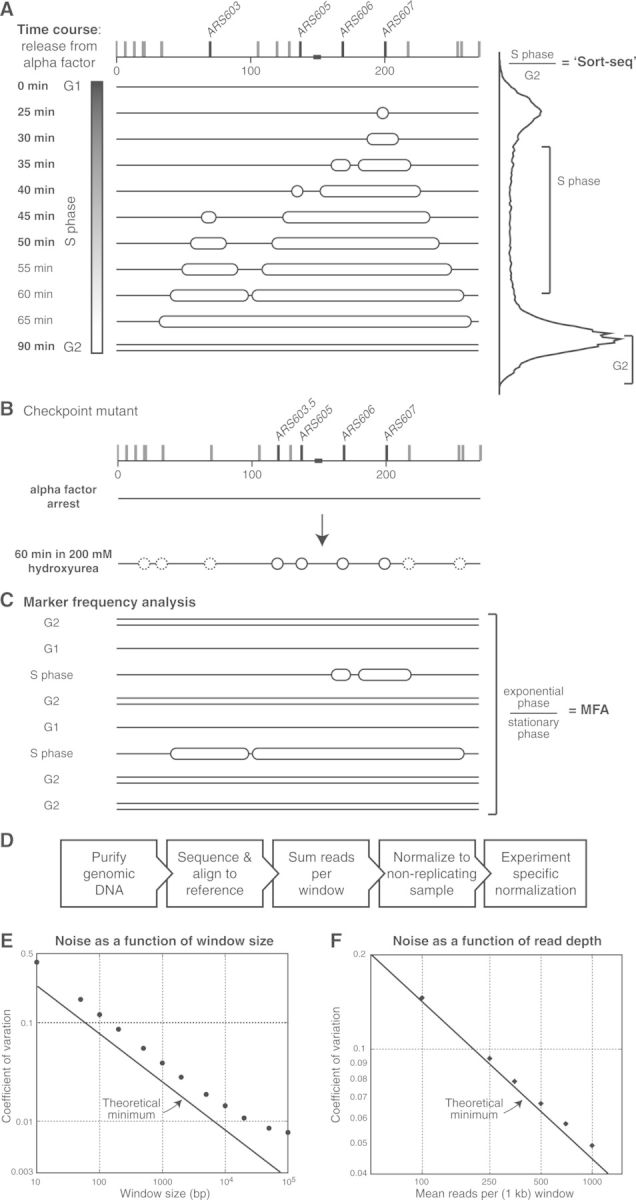
Deep sequencing measurements of DNA copy number to determine genome replication dynamics. (**A**) An overview of two strategies used to investigate genome replication. An example of *S. cerevisiae* chromosome 6 replication progression (top; replication origins marked by vertical ticks, centromere by box). (Left) Time points analyzed (bold); (right) schematic of flow cytometry profile indicating samples selected for typical sort-seq experiment. (**B**) Schematic of chromosome replication in checkpoint mutant subjected to replication stress (HU). Solid circles indicate early activating origins, dashed circles later activating origins. (**C**) MFA allows the measurement of copy number differences from exponential phase cultures (∼25% undergoing DNA replication) relative to stationary phase cultures (no replicating cells). (**D**) Overview of experimental strategy and data analyses. Relationship between experimental noise (coefficient of variation = standard deviation/mean) and various window sizes (**E**) and read depths (**F**). In each case, the points represent experimental values calculated from the sequencing data and the lines represent theoretical minimum values.

To determine the dynamics of genome replication in *S. cerevisiae*, deep sequencing was used to measure DNA copy number in replicating cells relative to nonreplicating cells (Figure 1A–D). Four different approaches were compared. First, checkpoint-deficient *S. cerevisiae* cells were synchronized in G1-phase (alpha factor arrest) and released into S-phase in the presence of the ribonucleotide reductase inhibitor HU. Second, for maximum temporal and spatial resolution cells were synchronously released from G1-phase and samples were taken during and after DNA replication. Third, replicating (S-phase) and nonreplicating (G2 phase) cells were selected by FACS. This ‘sort-seq' approach does not require cell synchronization and therefore allows the analysis of both haploid and diploid strains. Fourth, DNA from exponential and stationary phase cultures was directly sequenced to detect the enrichment in copy number at early-replicating relative to later-replicating sequences. The same genetic background (W303) was used throughout to allow direct comparisons between experiments.

To assess the precision with which deep sequencing can measure changes in relative DNA copy number, two nonreplicating samples from our time course experiment were compared. In these samples, relative copy number could be measured with a coefficient of variation of <4% at a spatial resolution of 1 kb. Increasing the number of reads mapping per window, either by increasing the window size or by increasing the total number of sequencing reads, further reduced this coefficient of variation (Figure 1E and F). The coefficient of variation determined from the experimental data was close to the theoretical minimum for a random distribution of reads to windows (compare data points with the line in Figure 1E and F). This demonstrates that deep sequencing offers a precise measure of relative DNA copy number and therefore has the potential to measure replication dynamics at high-resolution genome-wide.

### Genome-wide localization of replication origins

**Figure 2.**
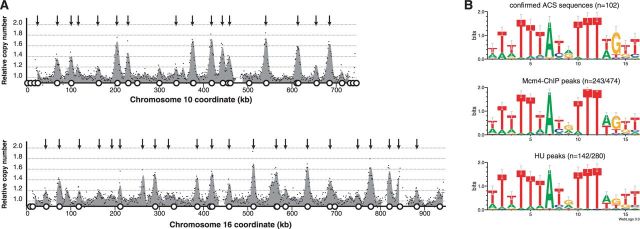
DNA copy number changes in a checkpoint mutant subjected to HU. (**A**) DNA copy number increase is restricted to origin proximal regions. The plots show the ratio between uniquely mapped sequencing reads from a G1- and S-phase sample, normalized to a baseline of 1. Regions lacking data are nonunique sequences where reads were not mapped. Black dots are the raw data points in 1 kb windows. Fourier transformation was applied to generate the smoothed profiles, shown in solid gray. Open circles represent known replication origin locations; arrows represent peak calls. The full genome is shown in Supplementary Figure S1. (**B**) HU peak calls allow rediscovery of the ORC-binding motif, the ACS. Sequence motifs were identified using GIMSAN from experimentally confirmed ACS (top), Mcm4 ChIP-seq peaks (middle) and HU peaks (bottom). The *n* refers to the number of motif occurrences identified (where appropriate relative to the number of peak calls).

Subjecting checkpoint-deficient cells to HU limits replication to regions proximal to origins, allowing the identification of initiation sites genome-wide (10). Haploid checkpoint-deficient (Δ*rad53* Δ*sml1*) cells were arrested in G1-phase and synchronously released into 200 mM HU. Samples were taken before release and 60 min after release into HU. Resulting DNA samples were subjected to deep sequencing. Ratios of uniquely mapped sequencing reads between the released and arrested sample were determined and normalized to a baseline of 1.0 (Figure 2A and Supplementary Figure S1). Genomic locations with a ratio of one had not been replicated in the released sample and are therefore present at the same copy number as in the arrested sample. Replication results in an increased copy number relative to G1-phase arrested cells and thus in a ratio above 1. The normalized ratios of the sequencing reads are a direct measure of the copy number and hence the amount of DNA synthesis that occurred during the 60 min in HU.

Genome-wide peak calling was performed to identify regions of DNA synthesis and thus potential replication origin locations. A total of 280 copy number peaks were detected with a median distance of 476 bp from previously defined origins (24). The HU peak locations allowed the identification of a sequence logo that strongly resembles ACS motifs determined from sites confirmed by mutagenesis or from sites we identified by Mcm4 ChIP-seq (Figure 2B, Supplementary Figure S2 and Supplementary Table S3). Therefore, copy number maxima coincide with locations of known replication origins at a resolution sufficient to rediscover the known origin sequence motif.

Peak heights represent the proportion of cells that replicated the sequence during the 60 min in HU. If a sequence was replicated in every cell of the population, the copy number would reach a theoretical maximum of 2. A range of peak heights was observed with a maximum of ∼1.8. Differences in the peak height associated with an origin could reflect differences in origin activity. For example, origins might not be licensed in every cell and therefore they would not be competent to activate in some cells (25). In addition, origins that activate later will experience lower dNTP concentrations, which may result in origin unwinding but no nascent strand synthesis, giving extended regions of single-stranded DNA (26). Therefore, later-activating origins would be anticipated to give rise to lower peaks as a consequence of reduced DNA synthesis. Comparison between HU peak height and the median replication time of an origin (see below) revealed a clear negative correlation (Supplementary Figure S3). Therefore, peak heights in HU data are influenced by origin activation time and provide a proxy for activation time.

### High temporal and spatial resolution replication dynamics

**Figure 3.**
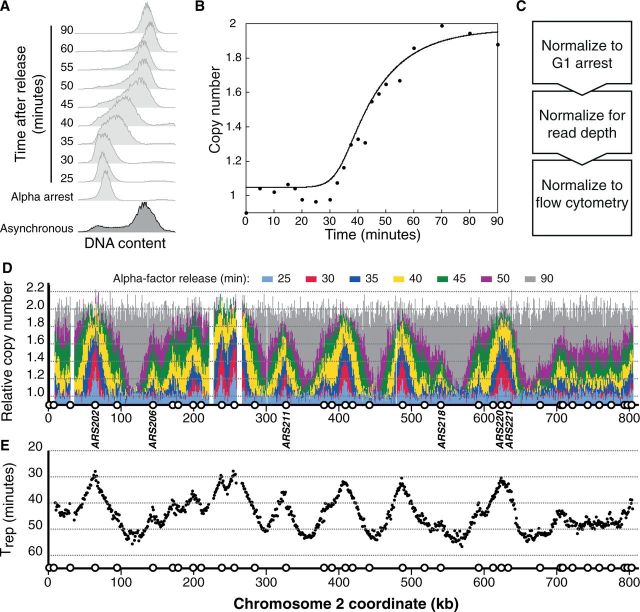
Time course analysis of genome replication. (**A**) Flow cytometry of a culture synchronously released from alpha factor arrest. (**B**) Quantification (with curve fit) of flow cytometry data for subsequent use in data normalization. (**C**) Data normalization steps. (**D**) *S. cerevisiae* chromosome 2 replication dynamics from seven time points through a synchronous S-phase (each normalized to the alpha factor arrest): 25 (light blue), 30 (red), 35 (blue), 40 (gold), 45 (green), 50 (purple) and 90 min (gray). Open circles represent known replication origin locations with six replication origins (as described in the text) labeled. The full genome is shown in Supplementary Figure S4. (**E**) Median replication time (Trep), calculated from the time course data. The full genome is shown in Supplementary Figure S5.

For maximum temporal and spatial resolution, we analyzed genome replication in a synchronous population of *S. cerevisiae*. Haploid cells were synchronously released into S-phase from a G1 arrest and time points were taken throughout and after S-phase. Bulk genome replication was assessed by flow cytometry (Figure 3A) and used to determine the fraction of the genome replicated at each time point (Figure 3B). For each of the time points, the DNA copy number was calculated relative to G1-phase and normalized for read number and the fraction of the genome replicated (Figure 3C) (18).

Peaks in each time point represent genomic locations that are more replicated than flanking sequences—i.e. replication origins (Figure 3D and Supplementary Figure S4). Early activating replication origins give rise to clear peaks in the first two time points (at 25 and 30 min, for example, *ARS202* and *ARS211*, Figure 3D), whereas later activating origins give rise to clear peaks in later time points (for example, *ARS206* and *ARS218*, Figure 3D). As S-phase progresses, forks move further from their initiation sites and collide with forks from other origins. Such regions are represented by valleys in the profiles. The flatness of the 90-min time point illustrates that replication is complete and every part of the genome is equally represented at a copy number of two.

The time at which each location has been replicated in half the cells (Trep) can be inferred from the time course data (Figure 3D and Supplementary Figure S5). The Trep profiles correlate with and offer increased resolution over previously published replication profiles (Supplementary Figure S6). Sharp peaks in replication profiles are a consequence of defined replication origins (25). Previously we have shown that smoothing replication timing data shifts peak positions and obscures their sharpness (25). The high spatial resolution of our data negates the need for smoothing. Therefore the sharp peak signatures of discrete origins can be resolved at individual time points and in the Trep curves (Figure 3D and E; Supplementary Figure S5). The Trep profiles also show smooth valleys, consistent with broad zones of replication termination due to stochastic origin activation (25,27).

Characterizing the activity of closely spaced origins from genome-wide replication profiles can be difficult since the peaks may not be distinguishable. The high spatial resolution of our data enables us to resolve multiple peaks at clusters of origins. For example, we can distinguish two peaks that arise from the activity of a pair of origins 9 kb apart (*ARS220*: 623 kb and *ARS221*: 632 kb) (Figure 3D) that previous studies could not (18,19,28). Finally, we called replication origin locations from our Trep data and compared them with confirmed origin locations (24). Peaks in our unsmoothed Trep data have a median distance of 1.2 kb from confirmed origin locations. In contrast origin locations called from previous Trep data (19) had a median distance of 3.4 kb (Supplementary Figure S7).

Analysis of genome replication in highly synchronous cultures by deep sequencing provides the highest temporal and spatial resolution currently available. Here we used mating pheromone (alpha factor) to achieve cell cycle synchronization, but alternatives include the use of conditional mutants (19), centrifugal elutriation of small daughter cells (29) or release from arrest induced by drug/nutrient deprivation that blocks a specific cell cycle stage (30). Synchronization may not be possible or desirable in all circumstances and therefore we investigated alternative approaches.

### Sort-Seq: replication dynamics from sorted asynchronous cell populations

**Figure 4.**
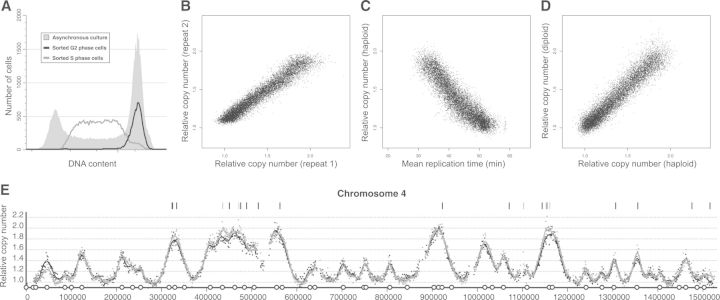
Sort-seq analysis reveals identical replication dynamics in haploids and diploids. (**A**) Flow cytometry of an asynchronous diploid *S. cerevisiae* culture (gray fill) and resulting S-phase enriched cells (gray line) and G2 enriched cells (black line). (**B**) Comparison of sort-seq from diploid *S. cerevisiae* for two biological replicates (repeat 1 used SOLiD; repeat 2 used Illumina sequencing). (**C**) Scatterplot of median replication time (Trep) versus relative copy number from sort-seq for haploid *S. cerevisiae*. (**D**) Scatterplot of relative copy number from sort-seq for haploid versus diploid *S.cerevisiae*. (**E**) Relative copy number replication profiles from sort-seq for *S.cerevisiae* chromosome 4. Dots are raw data points in 1 kb windows, and lines show smoothed profiles (black for diploid, gray for haploid). Bars above the profile indicate 1 kb windows that are significantly different (black for *P* < 0.001; gray for *P* < 0.01). The full genome is shown in Supplementary Figure S9.

It is possible that synchronizing cells with alpha factor alters the temporal dynamics of genome replication. To test this possibility we measured genome replication dynamics in an unperturbed cell population. FACS was used to enrich G2 phase (nonreplicating) and S-phase (replicating) cells from asynchronous cultures of haploid and diploid cells (Figure 4A). In S-phase, the copy number of a sequence is proportional to how early it replicates, whereas in G2 phase, all genomic locations are present at equivalent copy number. The relative copy number of each genomic location was determined by deep sequencing and normalized to a baseline of 1.0, which resulted in observed maximum peak heights of ∼2.0. Comparison of biological replicates demonstrated the reproducibility of the method (Figure 4B and Supplementary Figure S8).

Plots of relative copy number along every chromosome are comparable with replication timing profiles. Mathematically, we have previously shown that mean copy number has a negative linear relationship with median replication time, Trep (31). Therefore, the data from our haploid sort-seq experiment were compared with the data from the time course experiment (Figure 4C). As anticipated, later median replication times corresponded to lower mean copy number values with a clear negative linear relationship (correlation coefficient of −0.90; Supplementary Figure S6). Minor deviations from the linear relationship are apparent for the latest replicating sequences (Trep > ∼45 min) consistent with reduced discrimination of late replication time in the sort-seq sample. This could result from underrepresentation of the late S-phase cells in the sorted replicating sample or minor contamination of the nonreplicating sample with late S-phase cells (Figure 4A). Comparison of genome-wide replication profiles from the sort-seq with median replication time from the synchronous time course experiment did not reveal any locations or origins with altered replication times. Therefore, we find no evidence that cell cycle synchronization with alpha factor alters replication dynamics.

We compared the dynamics of genome replication for the diploid and haploid strains and observed a strong correlation (Figure 4D; correlation coefficient 0.95). Genome-wide the relative replication times are virtually superimposable (Figure 4E and Supplementary Figure S9). To identify regions with altered replication dynamics, the statistical significance of differences in replication time was calculated (*P* > 0.99) for each pair of data points. In theory, if a replication origin altered in activity (in a particular experimental comparison), it would be anticipated that a large proportion of the loci replicated by that origin (i.e. the replicon) would have a significant change in replication time. In contrast, if there were no significant changes in replication origin activity a more random distribution of loci with altered replication time would be expected, arising from experimental error. Random sources of experimental error should manifest randomly throughout the genome and differences in replication time should be both positive and negative. By contrast, systematic errors (for example, due to subtle differences in the fraction of S-phase cells collected) could give rise to values that are consistently higher in one sample than the other and a nonrandom genomic distribution. We observe that the diploid sample has a slight bias to higher values than the haploid sample, and these loci predominantly correspond to the earliest replicating sequences (data not shown). This is consistent with minor differences in the fractions sorted being responsible for the observed differences. We conclude that genome replication dynamics in haploid and diploid strains are indistinguishable.

### The future: sequencing from exponentially growing cells

The methods described above use various approaches to enrich for cells that are undergoing genome replication. These approaches can be time-consuming and have the potential to introduce biases in the observed genome replication dynamics. By contrast, in some prokaryotic species, a high proportion of cells within an exponentially growing population are undergoing genome replication. Consequently MFA can be used to infer replication dynamics. For example, we have used deep sequencing to characterize genome replication directly from exponentially growing cells in *Escherichia coli* (32) and *Haloferax volcanii* (Hawkins *et al.*, manuscript under review). In *S. cerevisiae*, ∼25% cells are undergoing genome replication in an exponentially growing culture (fraction of S-phase cells from flow cytometry data). Therefore, it is anticipated that in exponential phase, early replicating sequences will be ∼25% more abundant than late replicating sequences. The resolution offered by deep sequencing should allow sufficiently low levels of noise (see above; Figure 1F) to measure differences in copy number within the range of 1–1.25.

**Figure 5.**
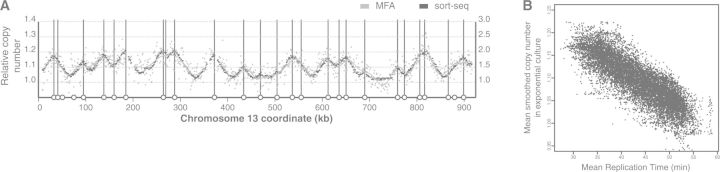
Direct measurement of genome replication in exponentially growing cells. (**A**) MFA as a proxy for replication time. Gray dots are the raw data points (in 1 kb windows), and the line shows the smoothed profile (left *y*-axis scale). Black dots show relative copy number from the (Illumina) sort-seq experiment (right *y*-axis scale). Open circles and vertical bars represent known replication origin locations. The full genome is shown in Supplementary Figure S10. (**B**) Comparison of MFA and median replication time (Trep).

Haploid cells were harvested in mid-log phase (replicating) and stationary phase (nonreplicating), and DNA copy number was measured by deep sequencing. Owing to the anticipated experimental noise, a smoothing algorithm was applied to the data. The maximum smoothed copy number enrichment at early versus late replicating sequences was ∼1.2, although this value will be an underestimate of the proportion of replicating cells as a consequence of smoothing (25). The resulting smoothed replication profile correlates with those obtained by sort-seq and the median replication time (Figure 5 and Supplementary Figure S10). The MFA data has higher noise than the other approaches; however, it clearly demonstrates the power of deep sequencing to measure replication dynamics in an unperturbed experiment.

## DISCUSSION

**Figure 6.**
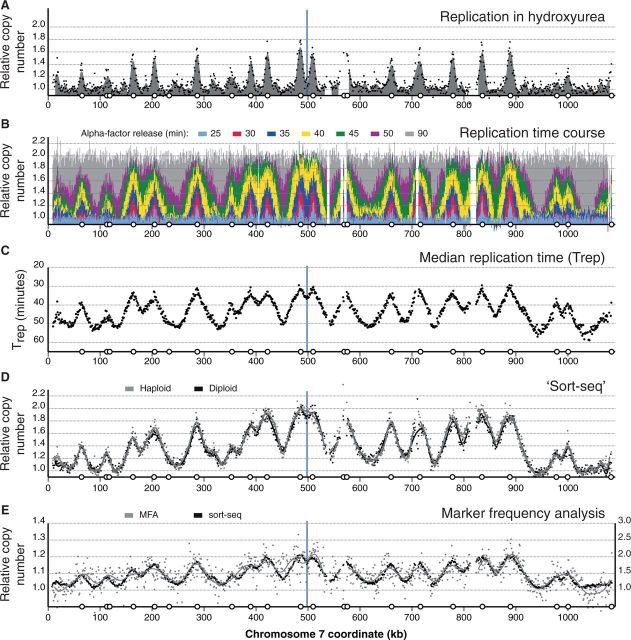
Juxtaposition of chromosome 7 data from the four copy number approaches to investigating genome replication. (**A**) DNA replication in a checkpoint mutant subjected to HU. (**B**) A time course through a synchronous S-phase (key as in legend to Figure 3). (**C**) Median replication time (Trep) as calculated from the time course data. (**D**) Sort-seq to directly compare the replication time of a haploid (gray) and diploid (black). (**E**) MFA as a proxy for replication time. Open circles represent known replication origin locations; blue bars represent the location of the centromere.

Here we present a systematic comparison of deep sequencing approaches to measure the dynamics of genome replication in a eukaryotic model organism (Figure 6). These methods measure the changes in DNA copy number resulting from DNA synthesis. These data provide a quantitative measure of the fraction of cells that have replicated a particular sequence in each sample. This approach is in contrast to a number of alternatives (e.g. BrdU ChIP or accumulation of single-stranded DNA) where the signal has no direct quantitative meaning, making comparisons between samples difficult. However, the challenge for DNA copy number–based approaches is the low signal-to-noise ratio, with a maximum difference of 2-fold between early- and late-replicating sequences, and with a significantly reduced range when using MFA. Here we demonstrate that deep sequencing can provide high temporal and spatial resolution replication profiles.

Application of these methods to other organisms will need consideration of the genome size, nature of genome replication and availability of cell cycle synchronization techniques. Obtaining >1000 reads per kb allowed us to achieve ∼5% standard deviation at 1 kb resolution. Therefore, large mammalian genomes will require proportionally more reads. For example, the sort-seq methodology has already been applied to cultured human cells with generation of ∼100 reads per kb (33). Larger genomes are generally replicated over a longer period (i.e require longer S-phases), which, coupled with the observation that in some organisms individual origins activate in only a small fraction of cell cycles, presents additional challenges. Surmounting these challenges may require the sorting of multiple S-phase fractions or the use of DNA replication inhibitors.

We used HU to limit replication and allow the precise detection of replication initiation sites (Figure 6A and Supplementary Figure S1). The accuracy of the identified origin locations was sufficient to rediscover the ORC-binding motif. The highly quantitative measure of the degree and extent of DNA synthesis from each origin allows not only direct comparisons between origins, but will also permit direct comparisons between strains and conditions. Furthermore, origin peak heights in HU provide an indirect measure of origin replication time.

The time course data from a synchronous cell population gave replication dynamics with the highest spatial and temporal resolution (Figure 6B and C), but required cell cycle synchronization and the sequencing of several samples per experiment. The sort-seq approach offers a compromise between resolution, ease of use and the number of samples to be sequenced; furthermore, there is no requirement for strain manipulation or cell cycle perturbation. Although there is no direct time measurement, the median replication time of each sequence can be inferred based on the length of S-phase, which in turn can be approximated from the proportion of S-phase cells in the population and the doubling time (20). Therefore, care should be taken when making direct comparisons between sort-seq data from strains or organisms with differences in the duration of S-phase. We have successfully applied sort-seq to a range of yeast species and strains (9,34). Here we use this approach to demonstrate for (as far as we are aware) the first time that genome replication in haploids and diploids is indistinguishable (Figure 6D).

Finally, we present the first whole-genome application of MFA in a eukaryote (Figure 6E). The removal of processing steps (e.g. FACS) avoids the minor biases that they can introduce (Supplementary Figure S6). As with sort-seq, the MFA offers no direct measure of time, but the resulting data correlate with replication time (Supplementary Figure S6). The low signal (relative to the current levels of experimental noise) in MFA will require a significant increase in the number of sequence reads if it is to provide the resolution currently available with sort-seq. This will particularly be the case in organisms where there are less dramatic differences in mean replication time across the genome, for example, for genomes replicated from many low-efficiency origins. Therefore, with current sequencing costs, we prefer approaches that enrich for cells undergoing genome replication. However, we foresee that direct sequencing of DNA samples from exponentially growing cells might become the most appropriate approach as sequencing costs decrease and read numbers increase. For example, for many experimental systems, highly synchronized populations cannot be achieved, and in some cases, sorting might not be technically possible or desirable (e.g. when there are difficulties in defining the S-phase cells). The use of exponentially growing cells involves the least effort, minimizes perturbation and can be applied to any cultivable organism. Thus, it may be that one of the earliest methods for analyzing chromosome replication (35) will in the future become the approach of choice owing to the dramatic developments in DNA sequencing.

## Data access

Mcm4 ChIP-seq sequence data has been deposited at the NCBI Short Read Archive under accession number SRX341889. The replication timing profiles and deep sequencing data have been submitted to the NCBI Gene Expression Omnibus (GEO) (http://www.ncbi.nlm.nih.gov/geo/) under accession numbers GSE42243 and GSE48212.

## SUPPLEMENTARY DATA

Supplementary Data are available at NAR Online, including [36–54].

## FUNDING

Biotechnology and Biological Sciences Research Council [BB/E023754/1, BB/G001596/1, BB/F00513X/1, BB/K007211/1]. Funding for open access charge: University of Nottingham Open Accesss Fund.

*Conflict of interest statement*. None declared.

## Supplementary Material

Supplementary Data

## References

[1] Newman TJ, Mamun MA, Nieduszynski CA, Blow JJ (2013). Replisome stall events have shaped the distribution of replication origins in the genomes of yeasts. Nucleic Acids Res..

[2] Mechali M (2010). Eukaryotic DNA replication origins: many choices for appropriate answers. Nat. Rev. Mol. Cell Biol..

[3] Rhind N, Gilbert DM (2013). DNA replication timing. Cold Spring Harb. Perspect. Biol..

[4] Mechali M, Yoshida K, Coulombe P, Pasero P (2013). Genetic and epigenetic determinants of DNA replication origins, position and activation. Curr. Opin. Genet. Dev..

[5] Knott SR, Peace JM, Ostrow AZ, Gan Y, Rex AE, Viggiani CJ, Tavare S, Aparicio OM (2012). Forkhead transcription factors establish origin timing and long-range clustering in *S. cerevisiae*. Cell.

[6] Knott SR, Viggiani CJ, Tavare S, Aparicio OM (2009). Genome-wide replication profiles indicate an expansive role for Rpd3L in regulating replication initiation timing or efficiency, and reveal genomic loci of Rpd3 function in *Saccharomyces cerevisiae*. Genes Dev..

[7] Lian HY, Robertson ED, Hiraga S, Alvino GM, Collingwood D, McCune HJ, Sridhar A, Brewer BJ, Raghuraman MK, Donaldson AD (2011). The effect of Ku on telomere replication time is mediated by telomere length but is independent of histone tail acetylation. Mol. Biol. Cell.

[8] Cosgrove AJ, Nieduszynski CA, Donaldson AD (2002). Ku complex controls the replication time of DNA in telomere regions. Genes Dev..

[9] Natsume T, Muller CA, Katou Y, Retkute R, Gierlinski M, Araki H, Blow JJ, Shirahige K, Nieduszynski CA, Tanaka TU (2013). Kinetochores coordinate pericentromeric cohesion and early DNA replication by Cdc7-Dbf4 kinase recruitment. Mol. Cell.

[10] Gilbert DM (2010). Evaluating genome-scale approaches to eukaryotic DNA replication. Nat. Rev. Genet..

[11] McGuffee SR, Smith DJ, Whitehouse I (2013). Quantitative, genome-wide analysis of eukaryotic replication initiation and termination. Mol. Cell.

[12] Liachko I, Youngblood RA, Keich U, Dunham MJ (2013). High-resolution mapping, characterization, and optimization of autonomously replicating sequences in yeast. Genome Res..

[13] Xu J, Yanagisawa Y, Tsankov AM, Hart C, Aoki K, Kommajosyula N, Steinmann KE, Bochicchio J, Russ C, Regev A (2012). Genome-wide identification and characterization of replication origins by deep sequencing. Genome Biol..

[14] Besnard E, Babled A, Lapasset L, Milhavet O, Parrinello H, Dantec C, Marin JM, Lemaitre JM (2012). Unraveling cell type-specific and reprogrammable human replication origin signatures associated with G-quadruplex consensus motifs. Nat. Struct. Mol. Biol..

[15] Mesner LD, Valsakumar V, Karnani N, Dutta A, Hamlin JL, Bekiranov S (2011). Bubble-chip analysis of human origin distributions demonstrates on a genomic scale significant clustering into zones and significant association with transcription. Genome Res..

[16] Eaton ML, Galani K, Kang S, Bell SP, MacAlpine DM (2010). Conserved nucleosome positioning defines replication origins. Genes Dev..

[17] Sekedat MD, Fenyo D, Rogers RS, Tackett AJ, Aitchison JD, Chait BT (2010). GINS motion reveals replication fork progression is remarkably uniform throughout the yeast genome. Mol. Syst. Biol..

[18] Yabuki N, Terashima H, Kitada K (2002). Mapping of early firing origins on a replication profile of budding yeast. Genes Cells.

[19] Raghuraman MK, Winzeler EA, Collingwood D, Hunt S, Wodicka L, Conway A, Lockhart DJ, Davis RW, Brewer BJ, Fangman WL (2001). Replication dynamics of the yeast genome. Science.

[20] Koren A, Soifer I, Barkai N (2010). MRC1-dependent scaling of the budding yeast DNA replication timing program. Genome Res..

[21] Nakato R, Itoh T, Shirahige K (2013). DROMPA: easy-to-handle peak calling and visualization software for the computational analysis and validation of ChIP-seq data. Genes Cells.

[22] De Piccoli G, Katou Y, Itoh T, Nakato R, Shirahige K, Labib K (2012). Replisome stability at defective DNA replication forks is independent of S phase checkpoint kinases. Mol. Cell.

[23] Ng P, Keich U (2008). GIMSAN: a Gibbs motif finder with significance analysis. Bioinformatics.

[24] Siow CC, Nieduszynska SR, Muller CA, Nieduszynski CA (2012). OriDB, the DNA replication origin database updated and extended. Nucleic Acids Res..

[25] de Moura AP, Retkute R, Hawkins M, Nieduszynski CA (2010). Mathematical modelling of whole chromosome replication. Nucleic Acids Res..

[26] Feng W, Collingwood D, Boeck ME, Fox LA, Alvino GM, Fangman WL, Raghuraman MK, Brewer BJ (2006). Genomic mapping of single-stranded DNA in hydroxyurea-challenged yeasts identifies origins of replication. Nat. Cell Biol..

[27] Retkute R, Nieduszynski CA, de Moura A (2011). Dynamics of DNA replication in yeast. Phys. Rev. Lett..

[28] Alvino GM, Collingwood D, Murphy JM, Delrow J, Brewer BJ, Raghuraman MK (2007). Replication in hydroxyurea: it's a matter of time. Mol. Cell. Biol..

[29] Agier N, Romano OM, Touzain F, Cosentino Lagomarsino M, Fischer G (2013). The spatiotemporal program of replication in the genome of *Lachancea kluyveri*. Genome Biol. Evol..

[30] Di Rienzi SC, Lindstrom KC, Mann T, Noble WS, Raghuraman MK, Brewer BJ (2012). Maintaining replication origins in the face of genomic change. Genome Res..

[31] Retkute R, Nieduszynski CA, de Moura A (2012). Mathematical modeling of genome replication. Phys. Rev. E.

[32] Rudolph CJ, Upton AL, Stockum A, Nieduszynski CA, Lloyd RG (2013). Avoiding chromosome pathology when replication forks collide. Nature.

[33] Koren A, Polak P, Nemesh J, Michaelson JJ, Sebat J, Sunyaev SR, McCarroll SA (2012). Differential relationship of DNA replication timing to different forms of human mutation and variation. Am. J. Hum. Genet..

[34] Muller CA, Nieduszynski CA (2012). Conservation of replication timing reveals global and local regulation of replication origin activity. Genome Res..

[35] Yoshikawa H, Sueoka N (1963). Sequential replication of Bacillus subtilis chromosome. I. Comparison of marker frequencies in exponential and stationary growth phases. Proc. Natl Acad. Sci. USA.

[36] Bouton AH, Smith MM (1986). Fine-structure analysis of the DNA sequence requirements for autonomous replication of *Saccharomyces cerevisiae* plasmids. Mol. Cell. Biol..

[37] Breier AM, Chatterji S, Cozzarelli NR (2004). Prediction of *Saccharomyces cerevisiae* replication origins. Genome Biol..

[38] Celniker SE, Sweder K, Srienc F, Bailey JE, Campbell JL (1984). Deletion mutations affecting autonomously replicating sequence ARS1 of *Saccharomyces cerevisiae*. Mol. Cell. Biol..

[39] Chang F, Theis JF, Miller J, Nieduszynski CA, Newlon CS, Weinreich M (2008). Analysis of chromosome III replicators reveals an unusual structure for the ARS318 silencer origin and a conserved WTW sequence within the origin recognition complex binding site. Mol. Cell. Biol..

[40] Crampton A, Chang F, Pappas DL, Frisch RL, Weinreich M (2008). An ARS element inhibits DNA replication through a SIR2-dependent mechanism. Mol. Cell.

[41] Friedman KL, Diller JD, Ferguson BM, Nyland SV, Brewer BJ, Fangman WL (1996). Multiple determinants controlling activation of yeast replication origins late in S phase. Genes Dev..

[42] Hoggard T, Shor E, Müller CA, Nieduszynski CA, Fox CA (2013). Linking ORC-origin binding mechanisms to the regulation of origin activation time. PLoS Genet..

[43] Huang RY, Kowalski D (1996). Multiple DNA elements in ARS305 determine replication origin activity in a yeast chromosome. Nucleic Acids Res..

[44] Kearsey S (1984). Structural requirements for the function of a yeast chromosomal replicator. Cell.

[45] Nieduszynski CA, Knox Y, Donaldson AD (2006). Genome-wide identification of replication origins in yeast by comparative genomics. Genes Dev..

[46] Palzkill TG, Newlon CS (1988). A yeast replication origin consists of multiple copies of a small conserved sequence. Cell.

[47] Sharma K, Weinberger M, Huberman JA (2001). Roles for internal and flanking sequences in regulating the activity of mating-type-silencer-associated replication origins in *Saccharomyces cerevisiae*. Genetics.

[48] Shirahige K, Iwasaki T, Rashid MB, Ogasawara N, Yoshikawa H (1993). Location and characterization of autonomously replicating sequences from chromosome VI of *Saccharomyces cerevisiae*. Mol. Cell. Biol..

[49] Theis JF, Newlon CS (1997). The ARS309 chromosomal replicator of *Saccharomyces cerevisiae* depends on an exceptional ARS consensus sequence. Proc. Natl Acad. Sci. USA.

[50] Theis JF, Newlon CS (2001). Two compound replication origins in *Saccharomyces cerevisiae* contain redundant origin recognition complex binding sites. Mol. Cell. Biol..

[51] Theis JF, Yang C, Schaefer CB, Newlon CS (1999). DNA sequence and functional analysis of homologous ARS elements of *Saccharomyces cerevisiae* and *S. carlsbergensis*. Genetics.

[52] Vujcic M, Miller CA, Kowalski D (1999). Activation of silent replication origins at autonomously replicating sequence elements near the HML locus in budding yeast. Mol. Cell. Biol..

[53] Xu W, Aparicio JG, Aparicio OM, Tavare S (2006). Genome-wide mapping of ORC and Mcm2p binding sites on tiling arrays and identification of essential ARS consensus sequences in S. cerevisiae. BMC Genomics.

[54] Yamashita M, Hori Y, Shinomiya T, Obuse C, Tsurimoto T, Yoshikawa H, Shirahige K (1997). The efficiency and timing of initiation of replication of multiple replicons of *Saccharomyces cerevisiae* chromosome VI. Genes Cells.

