# Supplementary material for: The dynamics of genome replication using deep sequencing
Source: Nucleic Acids Res. 2013 Oct 1;42(1):e3. doi: 10.1093/nar/gkt878 (PMC3874191; doi:10.1093/nar/gkt878)
Supplement: Supplementary Data [file supp_42_1_e3__index.html]

The dynamics of genome replication using deep sequencing — The dynamics of genome replication using deep sequencing — Supplementary Data 

# The dynamics of genome replication using deep sequencing

## Supplementary Data

files

**Files in this Data Supplement:**

- Supplementary Data - pdf file
