# Supplementary material for: The dynamics of genome replication using deep sequencing
Source: Nucleic Acids Res. 2013 Oct 1;42(1):e3. doi: 10.1093/nar/gkt878 (PMC3874191; doi:10.1093/nar/gkt878)
Supplement: Supplementary Data [file supp_gkt878_nar-02258-met-k-2013-File008.pdf]

Supplementary legends

Figure S1: DNA copy number changes in a checkpoint mutant subjected to HU. DNA copy number increase is restricted to origin proximal regions. The plots show the ratio between uniquely mapped sequencing reads from a G1 and S-phase sample, normalized to a baseline of 1. Regions lacking data are non-unique sequences where reads were not mapped. Black dots are the raw data points in 1 kb windows. Fourier transformation was applied to generate the smoothed profiles, shown in solid grey. Open circles represent known replication origin locations; arrows represent peak calls.

Figure S2: Mcm4 ChIP-seq data. Profile showing the enrichment of Mcm4 bound sequences in alpha factor arrested cells.

Figure S3: Scatter plots for comparisons between HU peak height and origin replication time. For each origin identified as a peak in the HU dataset the origin replication time is plotted against the HU peak height. (left) The median replication time (Trep in min, from time course analysis) is used as a measure of origin replication time. (right) The inferred mean origin activation time (Yang et al., 2010) is used as a measure of origin activation time. In each case correlation coefficients are given.

Figure S4: Time course analysis of genome replication. *S. cerevisiae* genome replication dynamics from seven time points through a synchronous S-phase (each normalized to the alpha factor arrest): 25 min (light blue), 30 min (red), 35 min (blue), 40 min (gold), 45 min (green), 50 min (violet) and 90 min (grey). Open circles represent known replication origin locations.

Figure S5: Median replication time from time course data. Open circles represent known replication origin locations.

Figure S6: Scatter plots for pairwise comparisons between the copy number approaches to measuring genome replication and two published studies (16,17). Correlation coefficients are given for each comparison.

Figure S7: Distance to nearest confirmed replication origin for peak calls from Trep data.

Figure S8: Comparions of sort-seq biological replicates. Relative copy number replication profiles for diploid *S. cerevisiae*. Replicate 1 (SOLiD sequencing) data points are shown in black, replicate 2 (Illumina sequencing) in red. Dots are raw data points in 1 kb windows.

Figure S9: Sort-seq analysis reveals identical replication dynamics in haploids and diploids. Relative copy number replication profiles from sort-seq for the *S. cerevisiae* genome. Dots are raw data points in 1 kb windows and lines show smoothed profiles (black for diploid, grey for haploid). Bars above the profile indicate 1 kb windows that are significantly different (black for $p < 0.001$; grey for $p < 0.01$).

Figure S10: Direct measurement of genome replication in exponentially growing cells. Marker frequency analysis as a proxy for replication time. Grey dots are the raw data points (in 1 kb windows) and the line shows the smoothed profile (left y-axis scale). Black dots show relative copy number from the (Illumina) sort-seq experiment (right y-axis scale). Open circles represent known replication origin locations; blue bars indicate centromere locations.

Table S1: Summary of deep sequencing experiments. Six different experiments are presented with the strain name and pertinent information from each genotype shown. For each experiment the following summary information is presented: the sequencing technology; the strain name; library construction method; a sample description; the mean DNA copy number as measured by flow cytometry (N/A – not applicable); total reads generated; and the total number of reads that mapped uniquely within the nuclear genome. In each case 50 bp sequencing reads were obtained.

Table S2: Genotypes of strains used in this study.

Table S3: Details of confirmed essential sequence elements at 102 replication origins.

Müller, Hawkins, et al. Figure S1

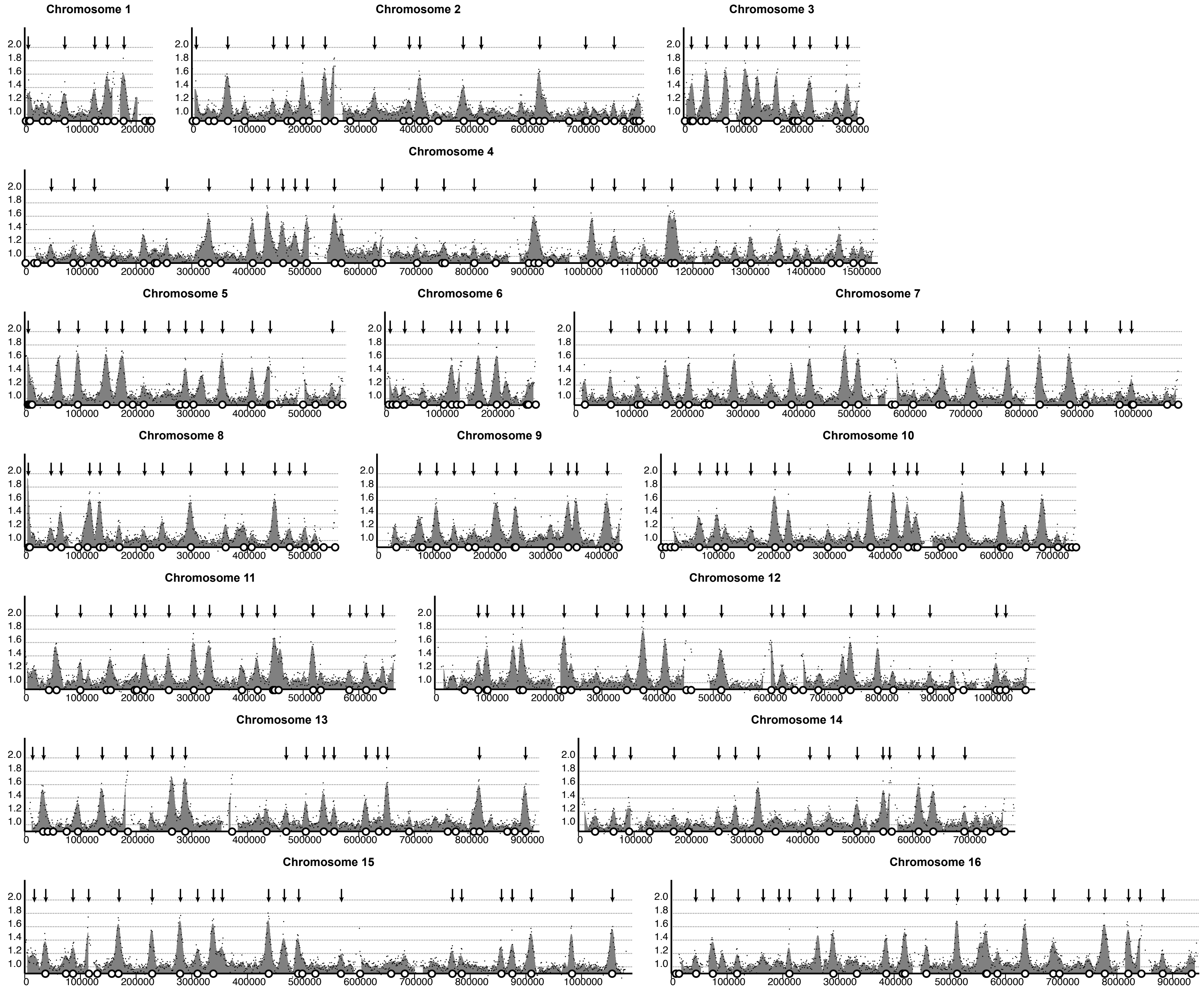

chrI

coding RNA
noncoding RNA
rRNA
LTR

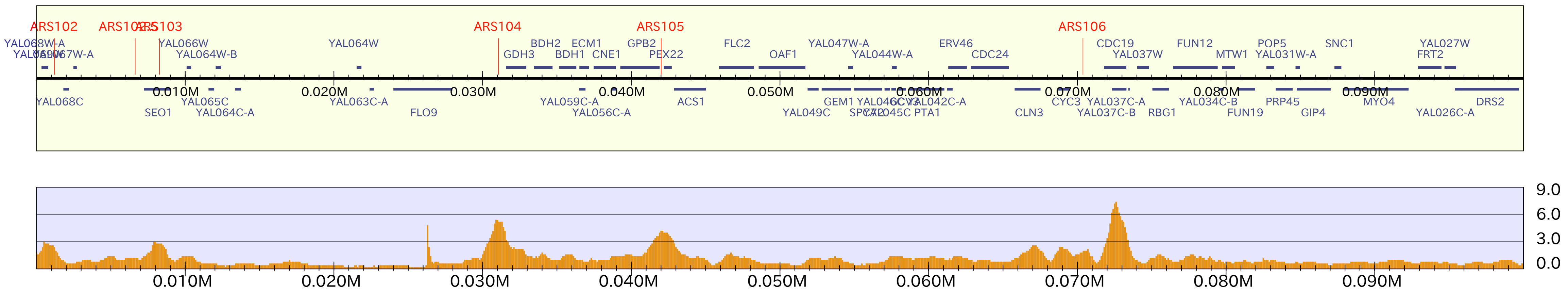

W303

coding RNA
noncoding RNA
rRNA
LTR

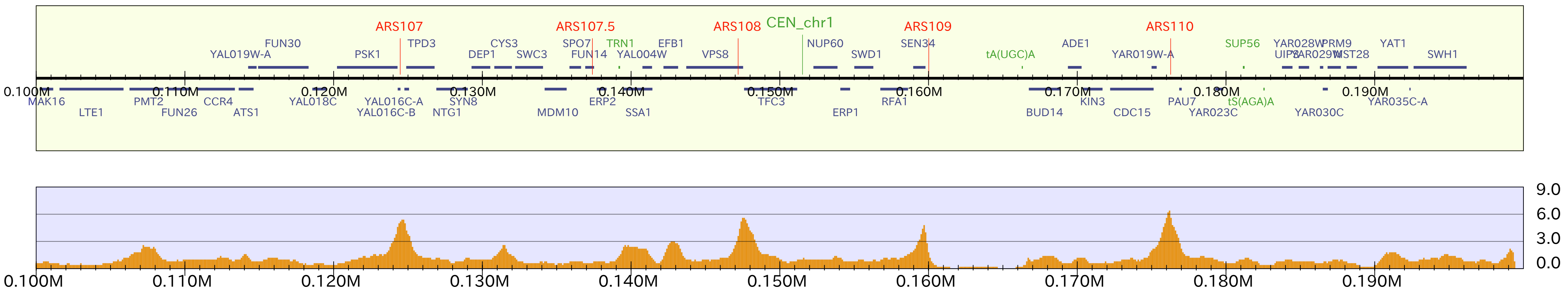

W303

coding RNA
noncoding RNA
rRNA
LTR

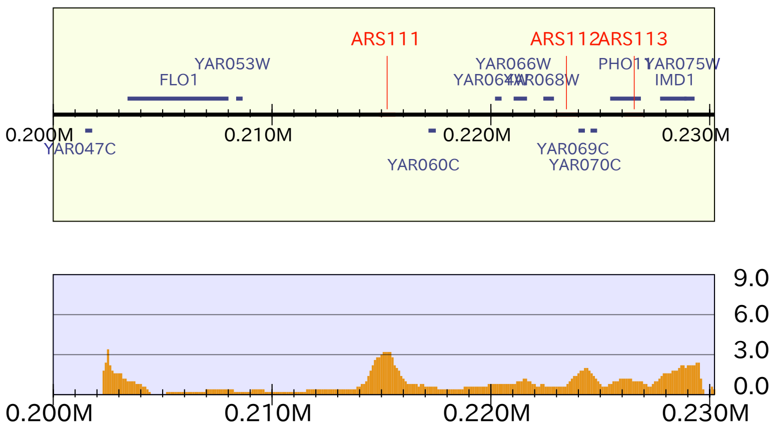

W303

chrII_1

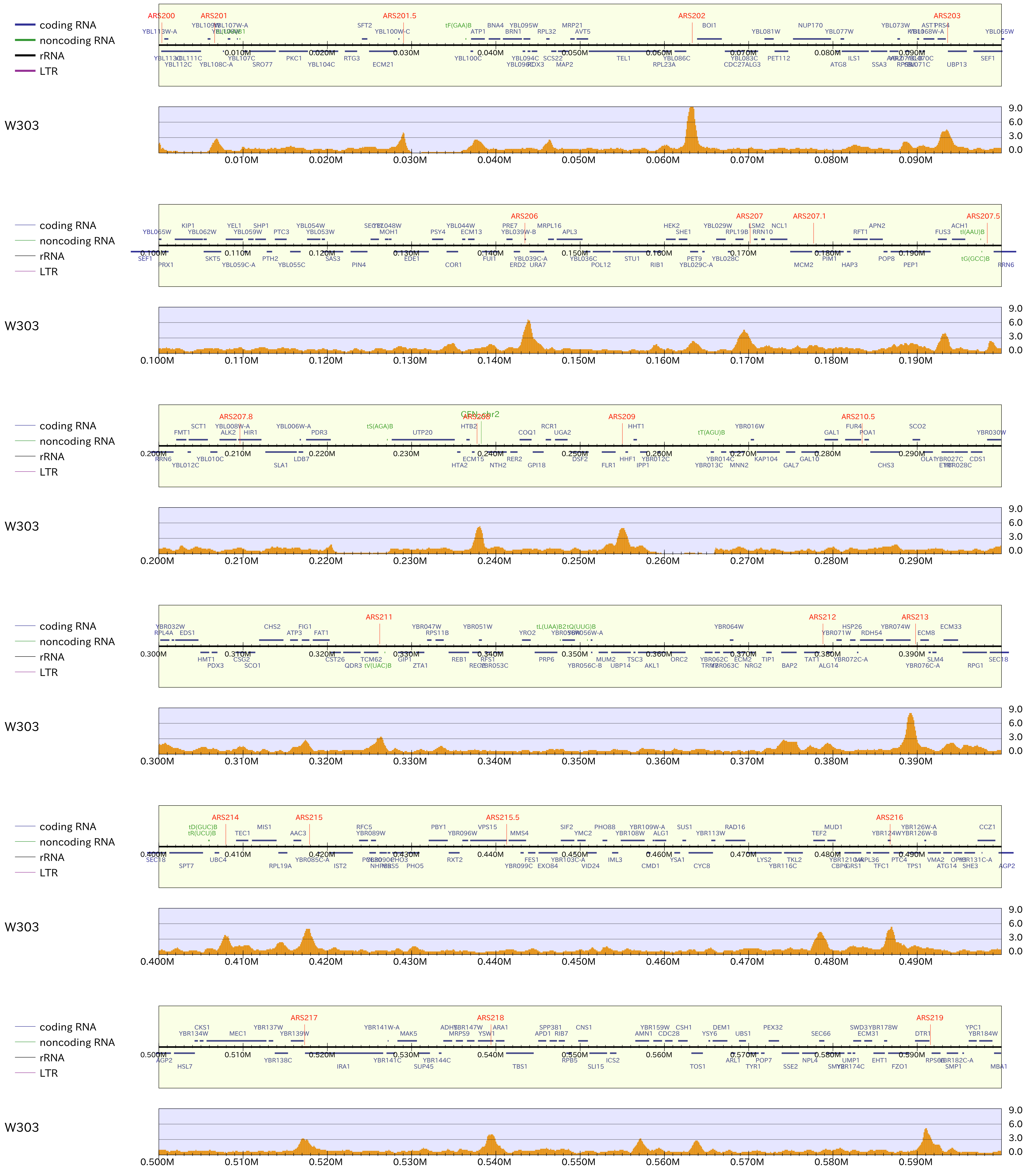
coding RNA
noncoding RNA
rRNA
LTR
W303
ARS200 ARS201 ARS201.5 ARS202 ARS203
ARS206 ARS207 ARS207.1 ARS207.5
ARS207.8 CEN_chr2 ARS208 ARS209 ARS210.5
ARS211 ARS212 ARS213
ARS214 ARS215 ARS215.5 ARS216
ARS217 ARS218 ARS219
9.0
6.0
3.0
0.0
0.010M 0.020M 0.030M 0.040M 0.050M 0.060M 0.070M 0.080M 0.090M
0.100M 0.110M 0.120M 0.130M 0.140M 0.150M 0.160M 0.170M 0.180M 0.190M
0.200M 0.210M 0.220M 0.230M 0.240M 0.250M 0.260M 0.270M 0.280M 0.290M
0.300M 0.310M 0.320M 0.330M 0.340M 0.350M 0.360M 0.370M 0.380M 0.390M
0.400M 0.410M 0.420M 0.430M 0.440M 0.450M 0.460M 0.470M 0.480M 0.490M
0.500M 0.510M 0.520M 0.530M 0.540M 0.550M 0.560M 0.570M 0.580M 0.590M

chrII_2

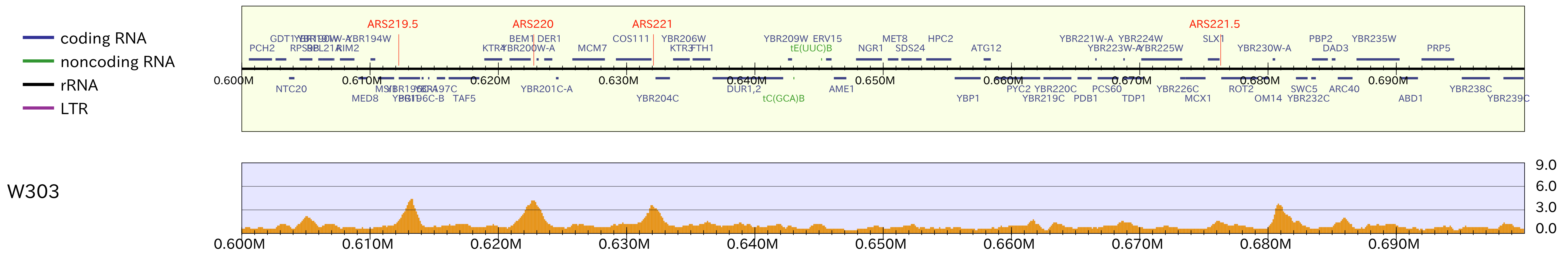
coding RNA
noncoding RNA
rRNA
LTR
ARS219.5
ARS220
ARS221
ARS221.5
PCH2
GDT1
RPS8B
AIM2
YBR194W
KTR4
BEM1
DER1
MCM7
COS111
YBR206W
KTR3
FTH1
YBR209W
ERV15
tE(UUC)B
NGR1
MET8
SDS24
HPC2
ATG12
YBR221W-A
YBR224W
YBR223W-A
YBR225W
SLX1
YBR230W-A
PBP2
DAD3
YBR235W
PRP5
0.600M
0.610M
0.620M
0.630M
0.640M
0.650M
0.660M
0.670M
0.680M
0.690M
NTC20
MED8
TAF5
YBR201C-A
YBR204C
DUR1,2
tC(GCA)B
AME1
YBP1
PYC2
YBR220C
YBR219C
PDB1
PCS60
TDP1
YBR226C
MCX1
ROT2
OM14
SWC5
YBR232C
ARC40
ABD1
YBR238C
YBR239C
W303
9.0
6.0
3.0
0.0

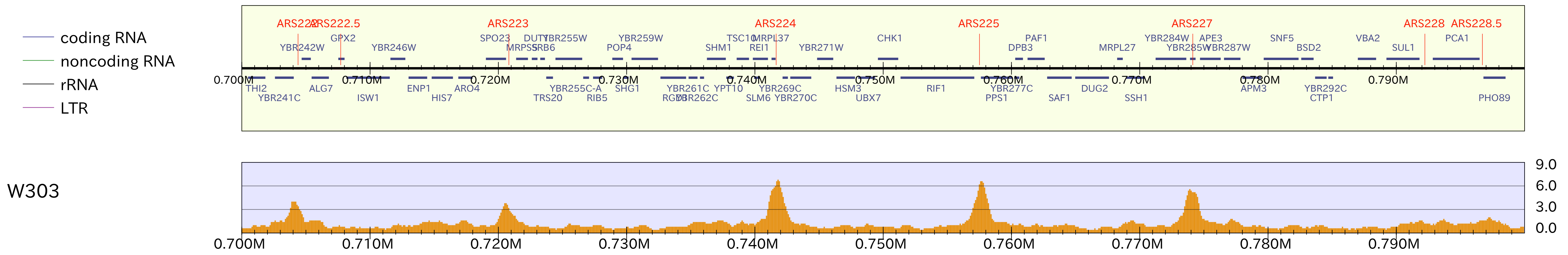
coding RNA
noncoding RNA
rRNA
LTR
ARS222
ARS222.5
ARS223
ARS224
ARS225
ARS227
ARS228
ARS228.5
YBR242W
GPX2
YBR246W
SPO23
DUT1
YBR255W
MRPS5
SRB6
YBR259W
POP4
SHM1
TSC10
MRPL37
REI1
YBR271W
CHK1
PAF1
DPB3
MRPL27
YBR284W
APE3
YBR287W
SNF5
BSD2
VBA2
SUL1
PCA1
0.700M
0.710M
0.720M
0.730M
0.740M
0.750M
0.760M
0.770M
0.780M
0.790M
THI2
YBR241C
ALG7
ISW1
ENP1
HIS7
ARO4
YBR255C-A
TRS20
RIB5
SHG1
YBR261C
YPT10
YBR262C
YBR269C
SLM6
YBR270C
HSM3
UBX7
RIF1
YBR277C
PPS1
SAF1
DUG2
SSH1
APM3
YBR292C
CTP1
PHO89
W303
9.0
6.0
3.0
0.0

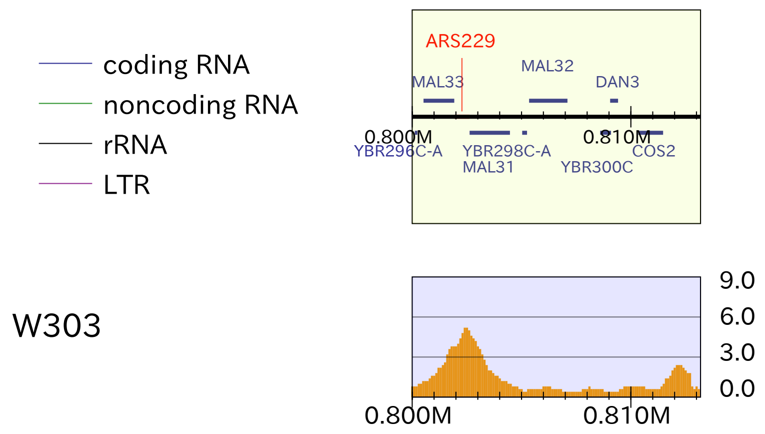
coding RNA
noncoding RNA
rRNA
LTR
ARS229
MAL33
MAL32
DAN3
0.800M
0.810M
YBR296C-A
YBR298C-A
MAL31
YBR300C
COS2
W303
9.0
6.0
3.0
0.0

chrIII

- coding RNA
- noncoding RNA
- rRNA
- LTR

W303

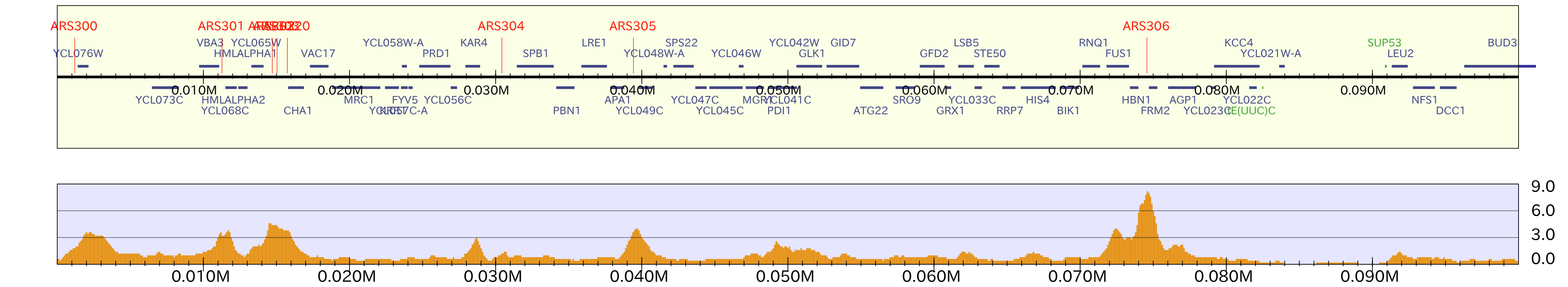

- coding RNA
- noncoding RNA
- rRNA
- LTR

W303

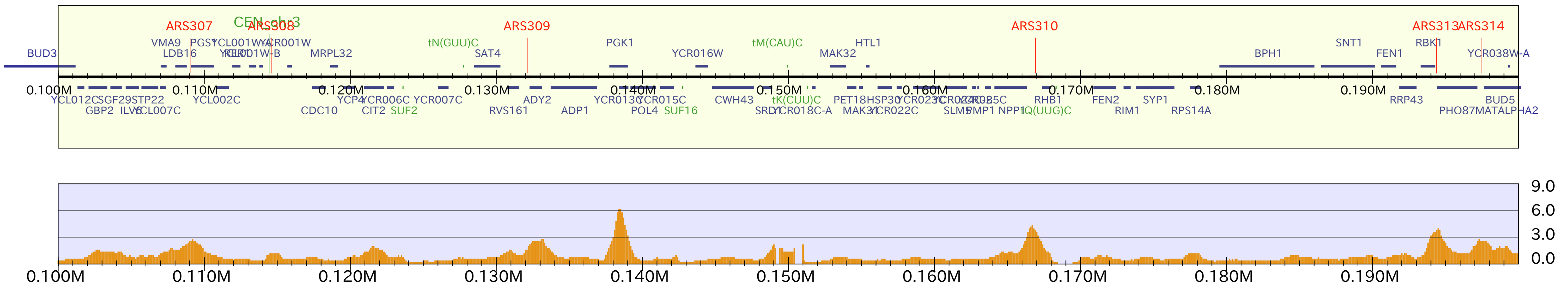

- coding RNA
- noncoding RNA
- rRNA
- LTR

W303

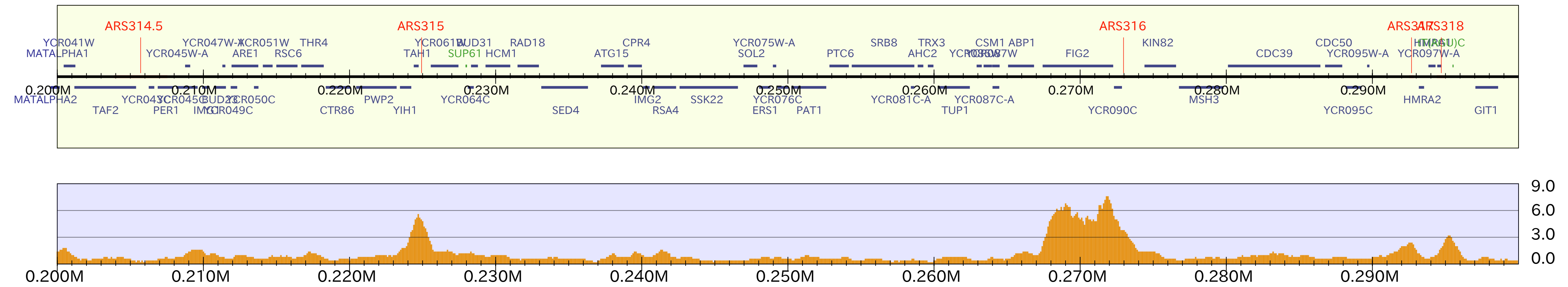

- coding RNA
- noncoding RNA
- rRNA
- LTR

W303

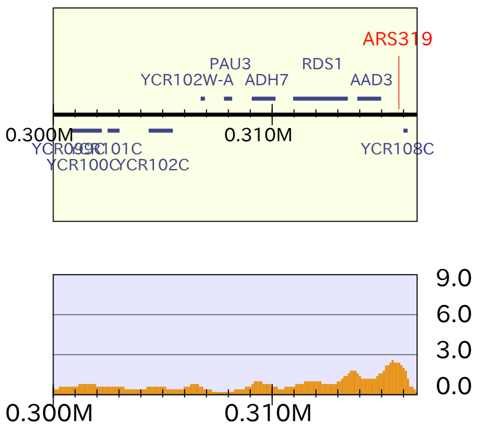

chrIV_1

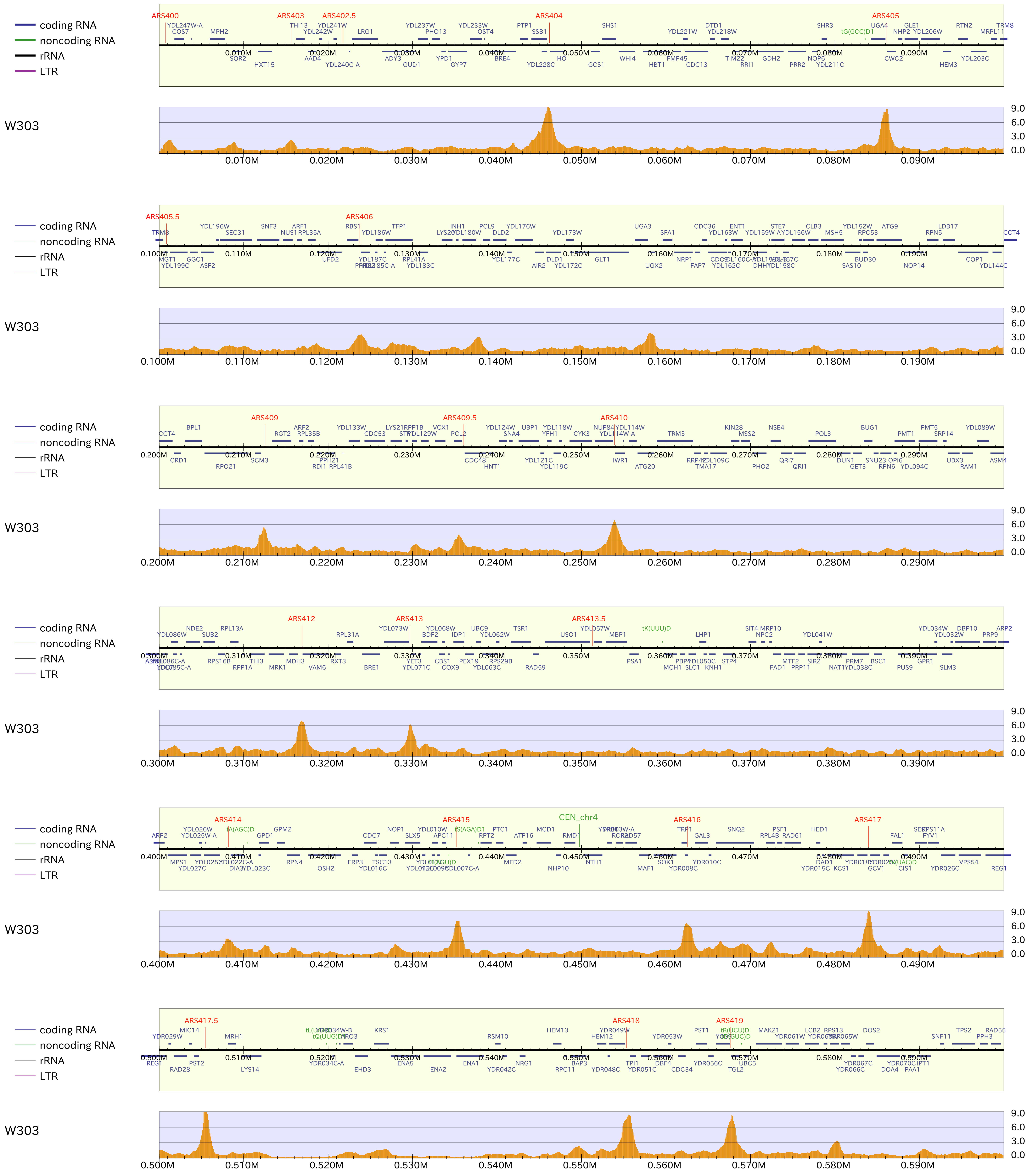

coding RNA
noncoding RNA
rRNA
LTR
W303
ARS400
ARS403
ARS402.5
ARS404
ARS405
ARS405.5
ARS406
ARS409
ARS409.5
ARS410
ARS412
ARS413
ARS413.5
ARS414
ARS415
CEN_chr4
ARS416
ARS417
ARS417.5
ARS418
ARS419
9.0
6.0
3.0
0.0
0.010M
0.020M
0.030M
0.040M
0.050M
0.060M
0.070M
0.080M
0.090M
0.100M
0.110M
0.120M
0.130M
0.140M
0.150M
0.160M
0.170M
0.180M
0.190M
0.200M
0.210M
0.220M
0.230M
0.240M
0.250M
0.260M
0.270M
0.280M
0.290M
0.300M
0.310M
0.320M
0.330M
0.340M
0.350M
0.360M
0.370M
0.380M
0.390M
0.400M
0.410M
0.420M
0.430M
0.440M
0.450M
0.460M
0.470M
0.480M
0.490M
0.500M
0.510M
0.520M
0.530M
0.540M
0.550M
0.560M
0.570M
0.580M
0.590M

chrIV_2

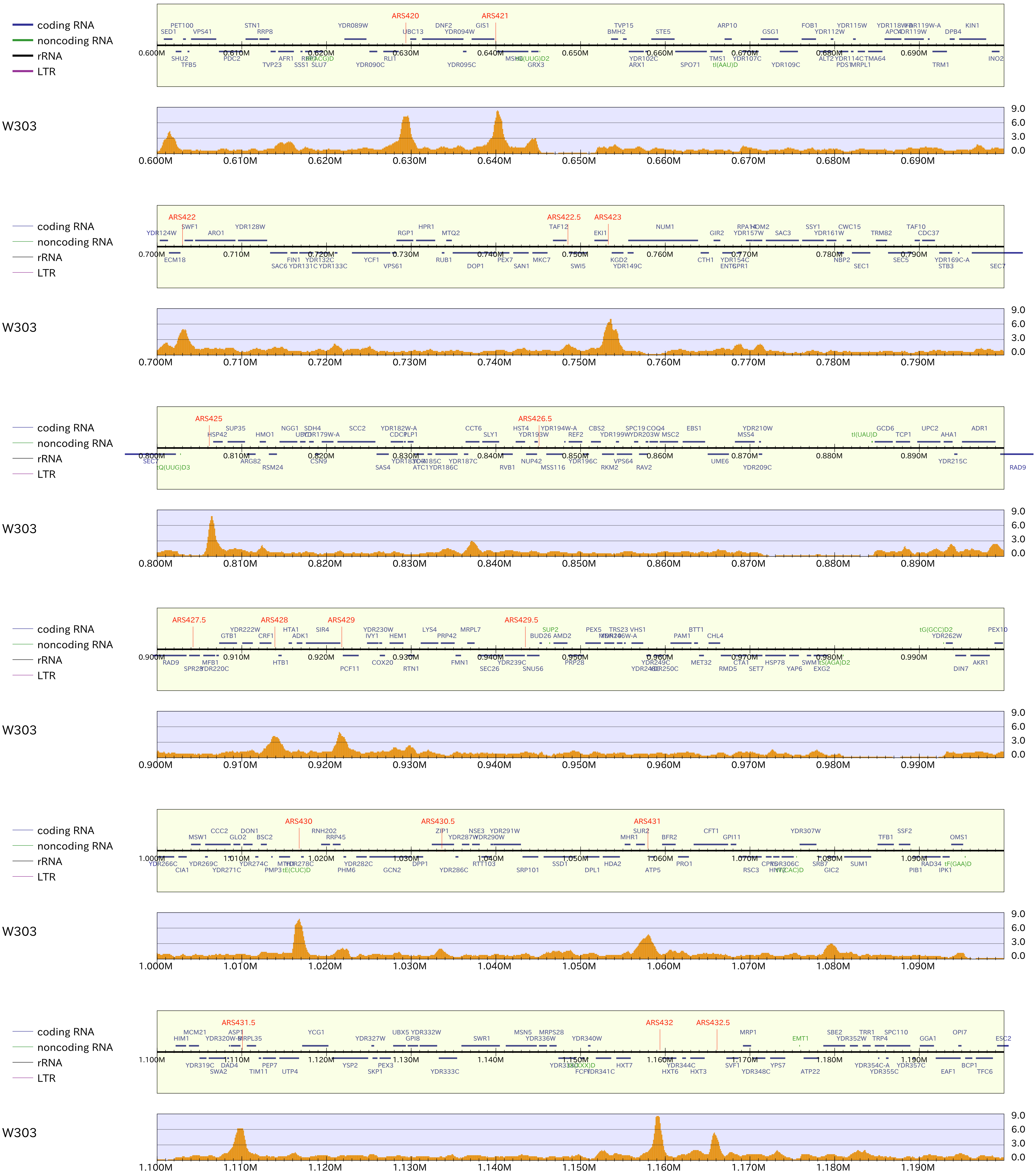

coding RNA
noncoding RNA
rRNA
LTR
ARS420
ARS421
PET100 SED1 VPS41 STN1 RRP8 YDR089W UBC13 DNF2 YDR094W GIS1 TVP15 BMH2 STE5 ARP10 GSG1 FOB1 YDR112W YDR115W APC4 YDR119W DPB4 KIN1
SHU2 TFB5 PDC2 AFR1 TVP23 SSS1 SLU7 RLI1 YDR090C YDR095C GRX3 YDR102C ARX1 SPO71 TMS1 tI(AAU)D YDR107C YDR109C ALT2 YDR114C TMA64 PDS1 MRPL1 TRM1 INO2
W303
0.600M 0.610M 0.620M 0.630M 0.640M 0.650M 0.660M 0.670M 0.680M 0.690M
9.0 6.0 3.0 0.0
ARS422
ARS422.5
ARS423
YDR124W SWF1 ARO1 YDR128W RGP1 HPR1 MTQ2 TAF12 EKI1 NUM1 GIR2 YDR157W SAC3 SSY1 YDR161W CWC15 TRM82 TAF10 CDC37
ECM18 FIN1 YDR132C SAC6 YDR131C YDR133C YCF1 VPS61 RUB1 DOP1 PEX7 SAN1 MKC7 SWI5 KGD2 YDR149C CTH1 YDR154C NBP2 SEC1 SEC5 YDR169C-A STB3 SEC7
0.700M 0.710M 0.720M 0.730M 0.740M 0.750M 0.760M 0.770M 0.780M 0.790M
ARS425
ARS426.5
HSP42 SUP35 HMO1 NGG1 SDH4 YDR179W-A SCC2 YDR182W-A CCT6 SLY1 HST4 YDR193W YDR194W-A REF2 CBS2 SPC19 COQ4 YDR199W YDR203W MSC2 EBS1 YDR210W MSS4 tI(UAU)D GCD6 TCP1 UPC2 AHA1 ADR1
SEC7 tQ(UUG)D3 ARG82 RSM24 CSN9 SAS4 ATC1 YDR186C YDR187C RVB1 NUP42 MSS116 YDR196C RKM2 VPS64 RAV2 UME6 YDR209C YDR215C RAD9
0.800M 0.810M 0.820M 0.830M 0.840M 0.850M 0.860M 0.870M 0.880M 0.890M
ARS427.5
ARS428
ARS429
ARS429.5
YDR222W GTB1 CRF1 HTA1 ADK1 SIR4 YDR230W IVY1 HEM1 LYS4 PRP42 MRPL7 SUP2 BUD26 AMD2 PEX5 TRS23 VHS1 PAM1 BTT1 CHL4 tG(GCC)D2 YDR262W PEX10
RAD9 MFB1 SPR28 YDR220C HTB1 PCF11 COX20 RTN1 FMN1 SEC26 YDR239C SNU56 PRP28 YDR249C YDR250C MET32 CTA1 RMD5 SET7 HSP78 YAP6 EXG2 DIN7 AKR1
0.900M 0.910M 0.920M 0.930M 0.940M 0.950M 0.960M 0.970M 0.980M 0.990M
ARS430
ARS430.5
ARS431
MSW1 CCC2 GLO2 DON1 BSC2 RNH202 RRP45 ZIP1 YDR287W NSE3 YDR290W YDR291W MHR1 SUR2 BFR2 CFT1 GPI11 YDR307W TFB1 SSF2 OMS1
YDR266C CIA1 YDR269C YDR271C YDR274C PMP3 YDR278C tE(CUC)D YDR282C PHM6 GCN2 DPP1 YDR286C RTT103 SRP101 SSD1 DPL1 HDA2 ATP5 PRO1 RSC3 YDR306C SRB7 GIC2 SUM1 RAD34 PIB1 tF(GAA)D IPK1
1.000M 1.010M 1.020M 1.030M 1.040M 1.050M 1.060M 1.070M 1.080M 1.090M
ARS431.5
ARS432
ARS432.5
HIM1 MCM21 YDR320W-B ASP1 MRPL35 YCG1 YDR327W UBX5 YDR332W GPI8 SWR1 MSN5 YDR336W MRPS28 YDR340W MRP1 EMT1 SBE2 YDR352W TRR1 TRP4 SPC110 GGA1 OPI7 ESC2
YDR319C DAD4 SWA2 PEP7 TIM11 UTP4 YSP2 PEX3 SKP1 YDR333C FCF1 YDR341C HXT7 YDR344C HXT6 HXT3 SVF1 YDR348C YPS7 ATP22 YDR354C-A YDR355C YDR357C BCP1 EAF1 TFC6
1.100M 1.110M 1.120M 1.130M 1.140M 1.150M 1.160M 1.170M 1.180M 1.190M

chrIV_3

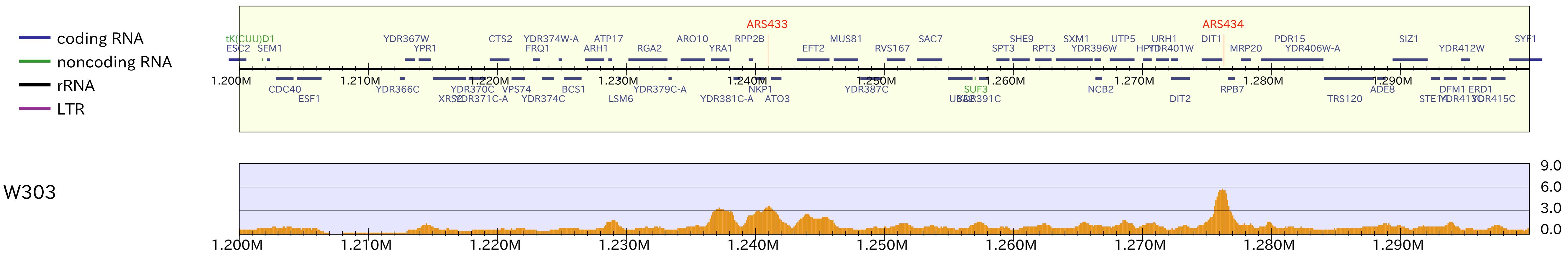
coding RNA
noncoding RNA
rRNA
LTR
ARS433
ARS434
tK(CUU)D1
ESC2 SEM1
YDR367W YPR1
CTS2 YDR374W-A FRQ1
ATP17 ARH1
RGA2
ARO10 YRA1
RPP2B
EFT2
MUS81
RVS167
SAC7
SHE9 SPT3
RPT3
SXM1 YDR396W
UTP5
URH1 HPT1 YDR401W
DIT1
MRP20
PDR15 YDR406W-A
SIZ1
YDR412W
SYF1
1.200M 1.210M 1.220M 1.230M 1.240M 1.250M 1.260M 1.270M 1.280M 1.290M
CDC40 ESF1
YDR366C
YDR370C XRS2 YDR371C-A
VPS74 YDR374C
BCS1
LSM6
YDR379C-A
YDR381C-A
NKP1 ATO3
YDR387C
SUF3
UBA2 YDR391C
NCB2
DIT2
RPB7
TRS120
ADE8
DFM1 ERD1 STE14 YDR413C YDR415C
W303
9.0 6.0 3.0 0.0

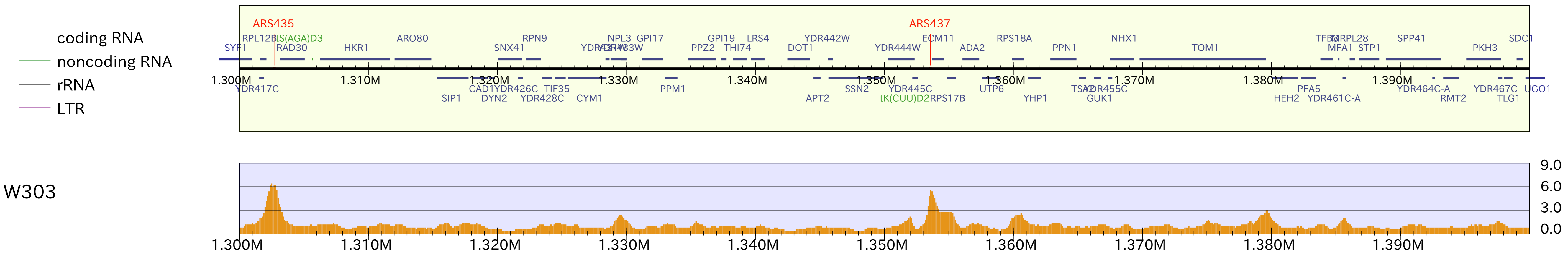
coding RNA
noncoding RNA
rRNA
LTR
ARS435
ARS437
SYF1
RPL12B tS(AGA)D3 RAD30
HKR1
ARO80
SNX41
RPN9
NPL3 GPI17 YDR432W YDR433W
GPI19 PPZ2
LRS4 THI74
YDR442W DOT1
YDR444W
ECM11
ADA2
RPS18A
PPN1
NHX1
TOM1
TFB3 MRPL28 MFA1 STP1
SPP41
PKH3
SDC1
1.300M 1.310M 1.320M 1.330M 1.340M 1.350M 1.360M 1.370M 1.380M 1.390M
YDR417C
SIP1
CAD1 YDR426C DYN2
TIF35 YDR428C
CYM1
PPM1
APT2
SSN2
YDR445C tK(CUU)D2 RPS17B
UTP6
YHP1
TSA2 YDR455C GUK1
HEH2
PFA5 YDR461C-A
YDR464C-A
RMT2
YDR467C TLG1
UGO1
W303
9.0 6.0 3.0 0.0

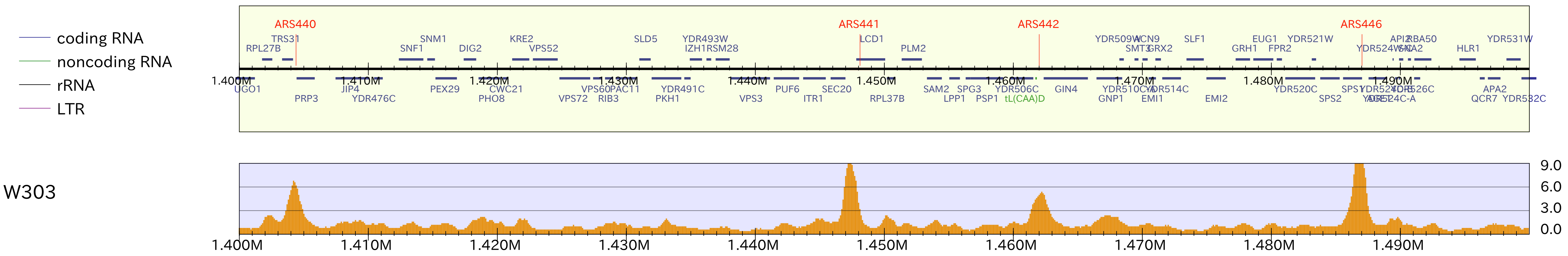
coding RNA
noncoding RNA
rRNA
LTR
ARS440
ARS441
ARS442
ARS446
RPL27B TRS31
SNF1 SNM1
DIG2
KRE2 VPS52
SLD5
YDR493W IZH1 RSM28
LCD1
PLM2
YDR509W SMT3 WCN9 GRX2
SLF1
EUG1 GRH1 FPR2
YDR521W
YDR524W-C API2 RBA50 SNA2
HLR1
YDR531W
1.400M 1.410M 1.420M 1.430M 1.440M 1.450M 1.460M 1.470M 1.480M 1.490M
UGO1
PRP3
JIP4 YDR476C
PEX29
CWC21 PHO8
VPS60 PAC11 VPS72 RIB3
YDR491C PKH1
VPS3
PUF6 ITR1
SEC20
RPL37B
SAM2 LPP1
SPG3 PSP1
YDR506C tL(CAA)D
GIN4
YDR510C GNP1
YDR514C EMI1
EMI2
YDR520C
SPS1 SPS2
APA2 QCR7
YDR532C
W303
9.0 6.0 3.0 0.0

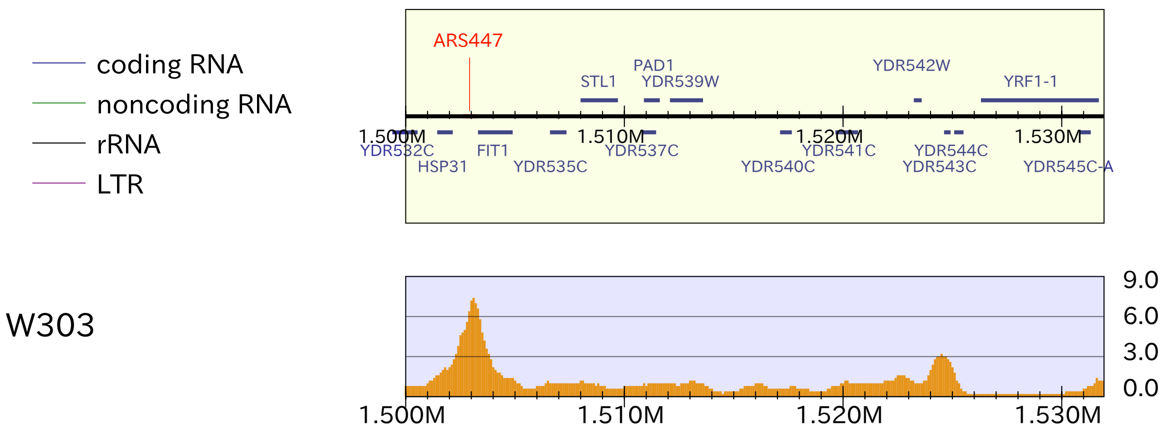
coding RNA
noncoding RNA
rRNA
LTR
ARS447
PAD1 STL1 YDR539W
YDR542W
YRF1-1
1.500M 1.510M 1.520M 1.530M
YDR532C HSP31
FIT1 YDR535C
YDR537C
YDR540C
YDR541C
YDR544C YDR543C
YDR545C-A
W303
9.0 6.0 3.0 0.0

chrV

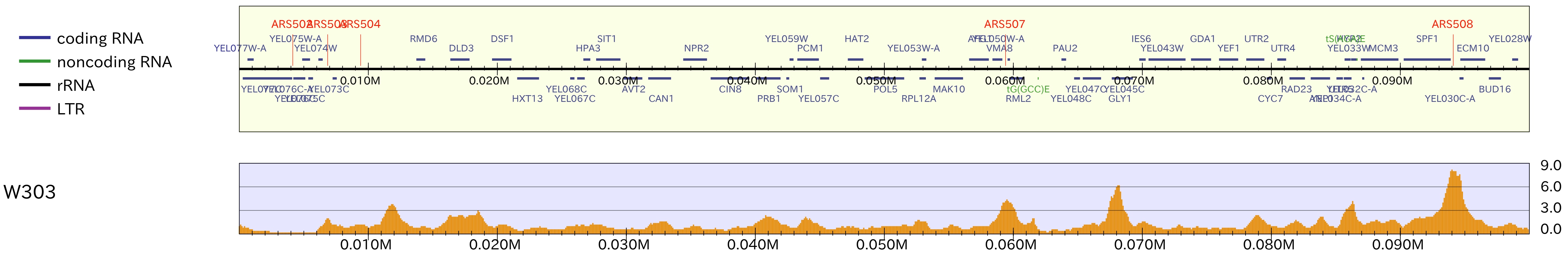
coding RNA
noncoding RNA
rRNA
LTR
ARS502
ARS505
ARS504
ARS507
ARS508
W303
9.0
6.0
3.0
0.0
0.010M
0.020M
0.030M
0.040M
0.050M
0.060M
0.070M
0.080M
0.090M

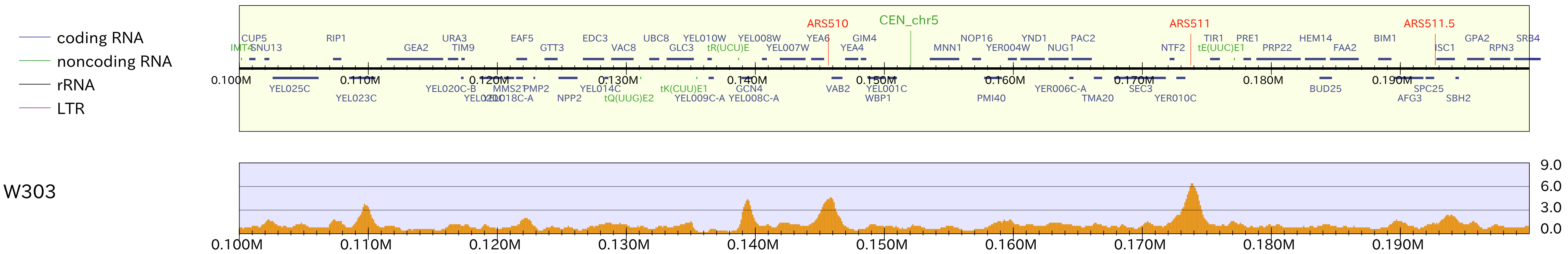
coding RNA
noncoding RNA
rRNA
LTR
ARS510
CEN_chr5
ARS511
ARS511.5
W303
9.0
6.0
3.0
0.0
0.100M
0.110M
0.120M
0.130M
0.140M
0.150M
0.160M
0.170M
0.180M
0.190M

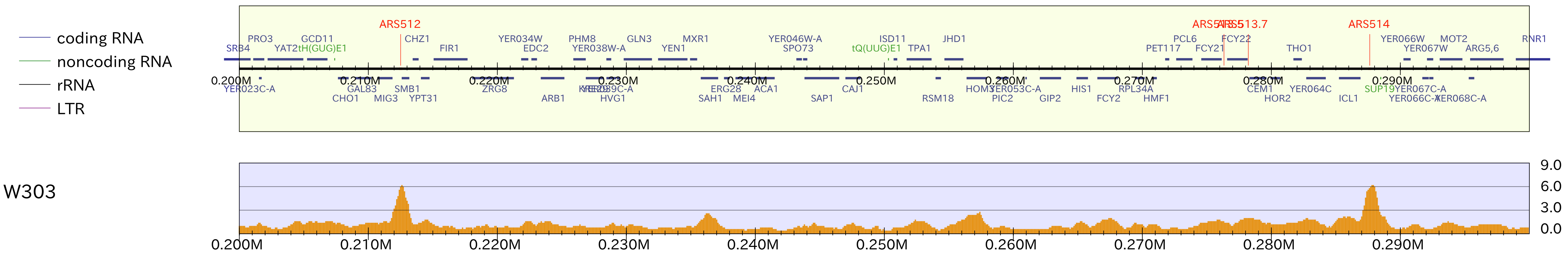
coding RNA
noncoding RNA
rRNA
LTR
ARS512
ARS514
W303
9.0
6.0
3.0
0.0
0.200M
0.210M
0.220M
0.230M
0.240M
0.250M
0.260M
0.270M
0.280M
0.290M

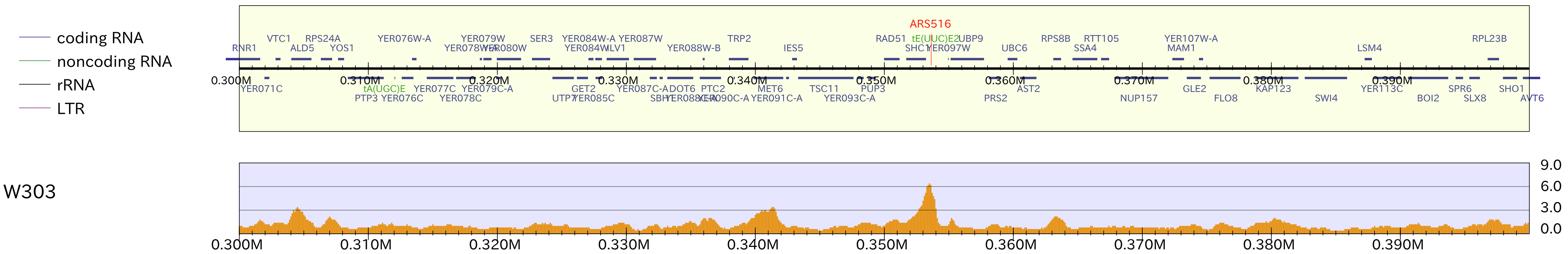
coding RNA
noncoding RNA
rRNA
LTR
ARS516
W303
9.0
6.0
3.0
0.0
0.300M
0.310M
0.320M
0.330M
0.340M
0.350M
0.360M
0.370M
0.380M
0.390M

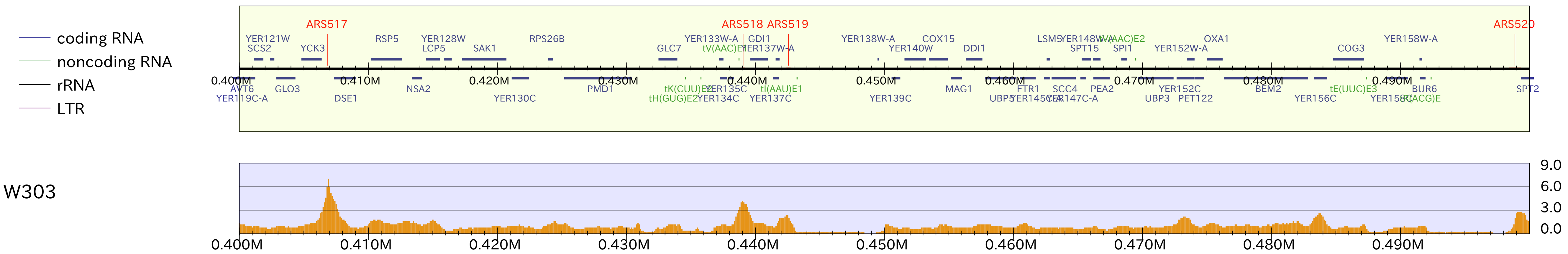
coding RNA
noncoding RNA
rRNA
LTR
ARS517
ARS518
ARS519
ARS520
W303
9.0
6.0
3.0
0.0
0.400M
0.410M
0.420M
0.430M
0.440M
0.450M
0.460M
0.470M
0.480M
0.490M

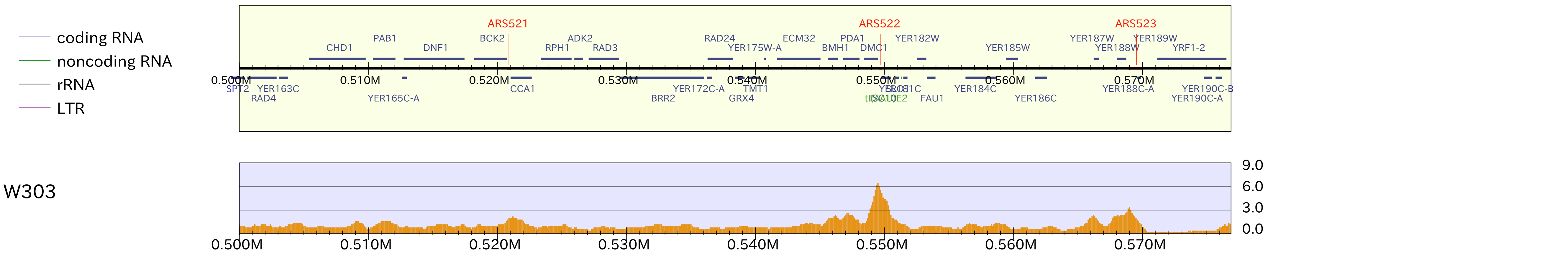
coding RNA
noncoding RNA
rRNA
LTR
ARS521
ARS522
ARS523
W303
9.0
6.0
3.0
0.0
0.500M
0.510M
0.520M
0.530M
0.540M
0.550M
0.560M
0.570M

chrVI

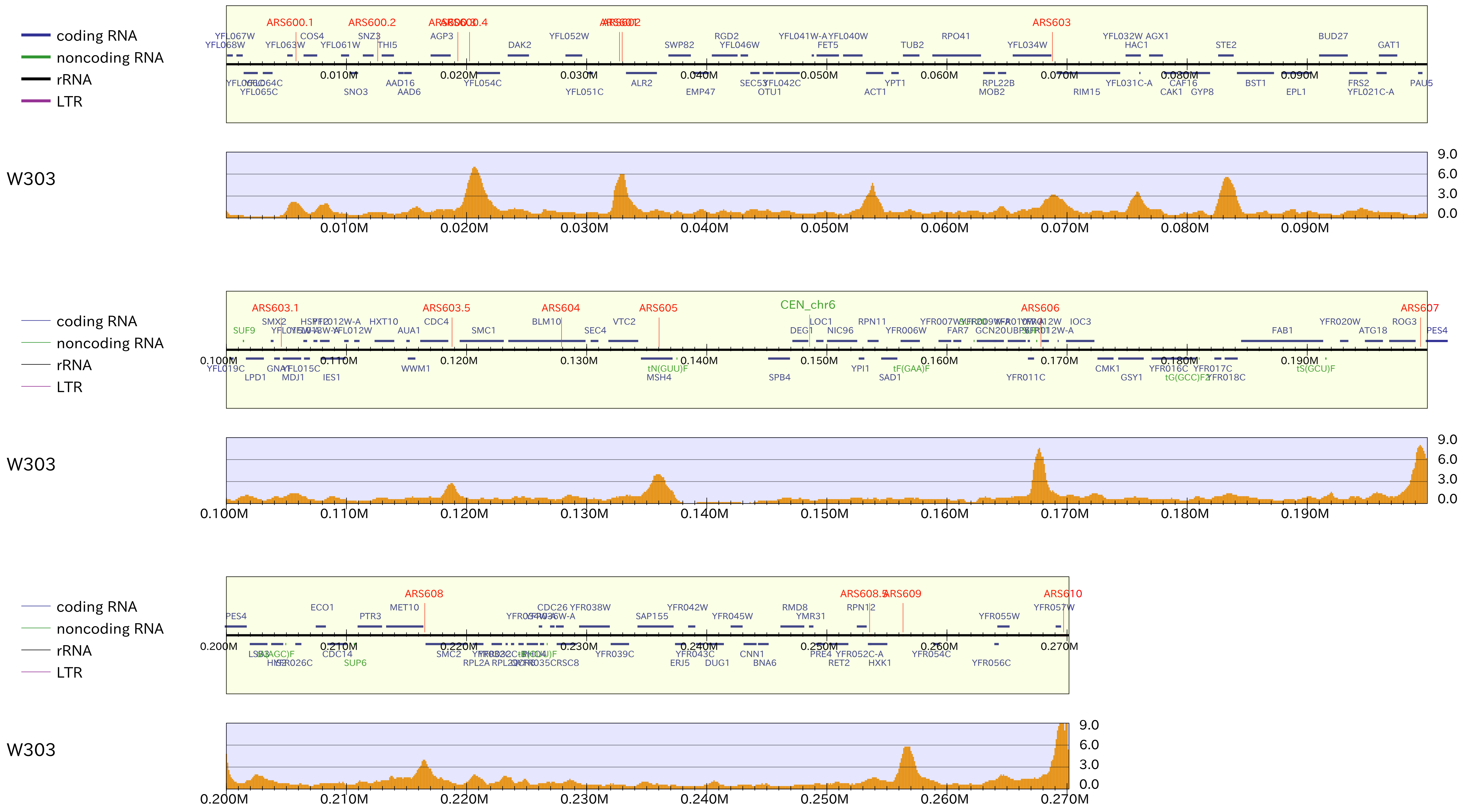
coding RNA
noncoding RNA
rRNA
LTR
W303
ARS600.1
ARS600.2
ARS603
CEN_chr6
ARS603.1
ARS603.5
ARS604
ARS605
ARS606
ARS607
ARS608
ARS609
ARS610
9.0
6.0
3.0
0.0
0.010M
0.020M
0.030M
0.040M
0.050M
0.060M
0.070M
0.080M
0.090M
0.100M
0.110M
0.120M
0.130M
0.140M
0.150M
0.160M
0.170M
0.180M
0.190M
0.200M
0.210M
0.220M
0.230M
0.240M
0.250M
0.260M
0.270M

chrVII_1

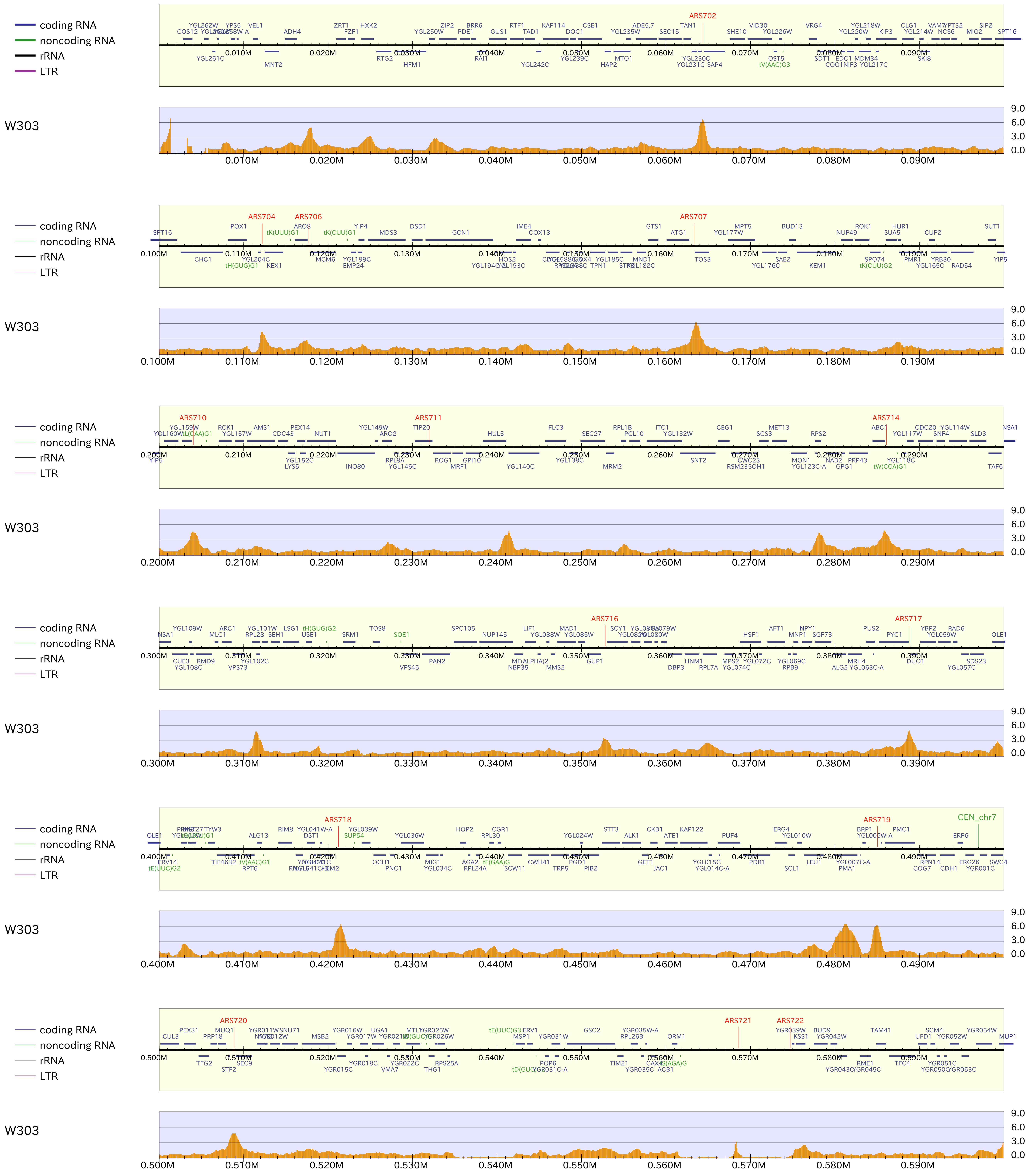

chrVII_2

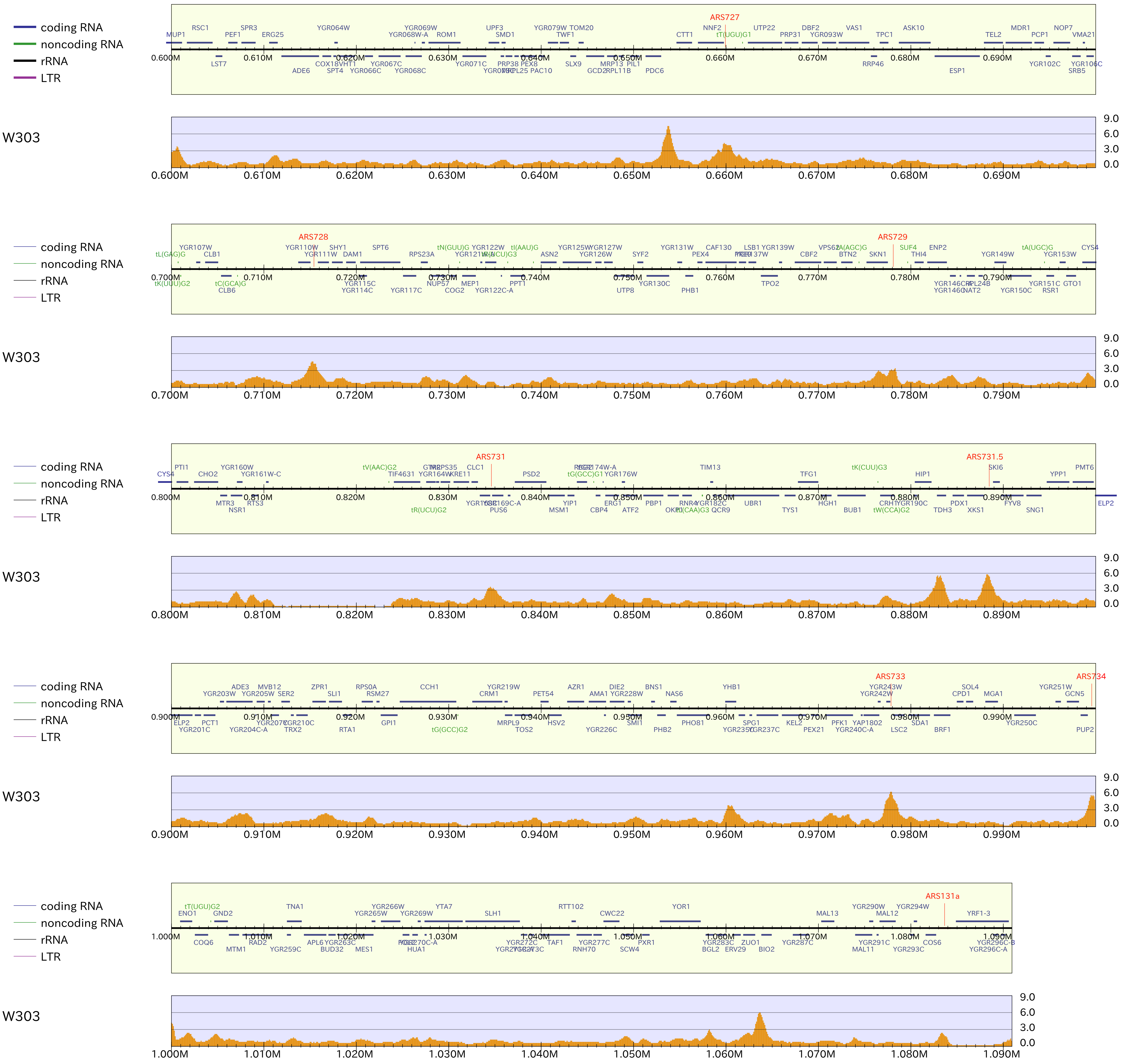
coding RNA
noncoding RNA
rRNA
LTR
ARS727
W303
9.0
6.0
3.0
0.0
0.600M
0.610M
0.620M
0.630M
0.640M
0.650M
0.660M
0.670M
0.680M
0.690M
ARS728
ARS729
0.700M
0.710M
0.720M
0.730M
0.740M
0.750M
0.760M
0.770M
0.780M
0.790M
ARS731
ARS731.5
0.800M
0.810M
0.820M
0.830M
0.840M
0.850M
0.860M
0.870M
0.880M
0.890M
ARS733
ARS734
0.900M
0.910M
0.920M
0.930M
0.940M
0.950M
0.960M
0.970M
0.980M
0.990M
ARS131a
1.000M
1.010M
1.020M
1.030M
1.040M
1.050M
1.060M
1.070M
1.080M
1.090M

chrVIII

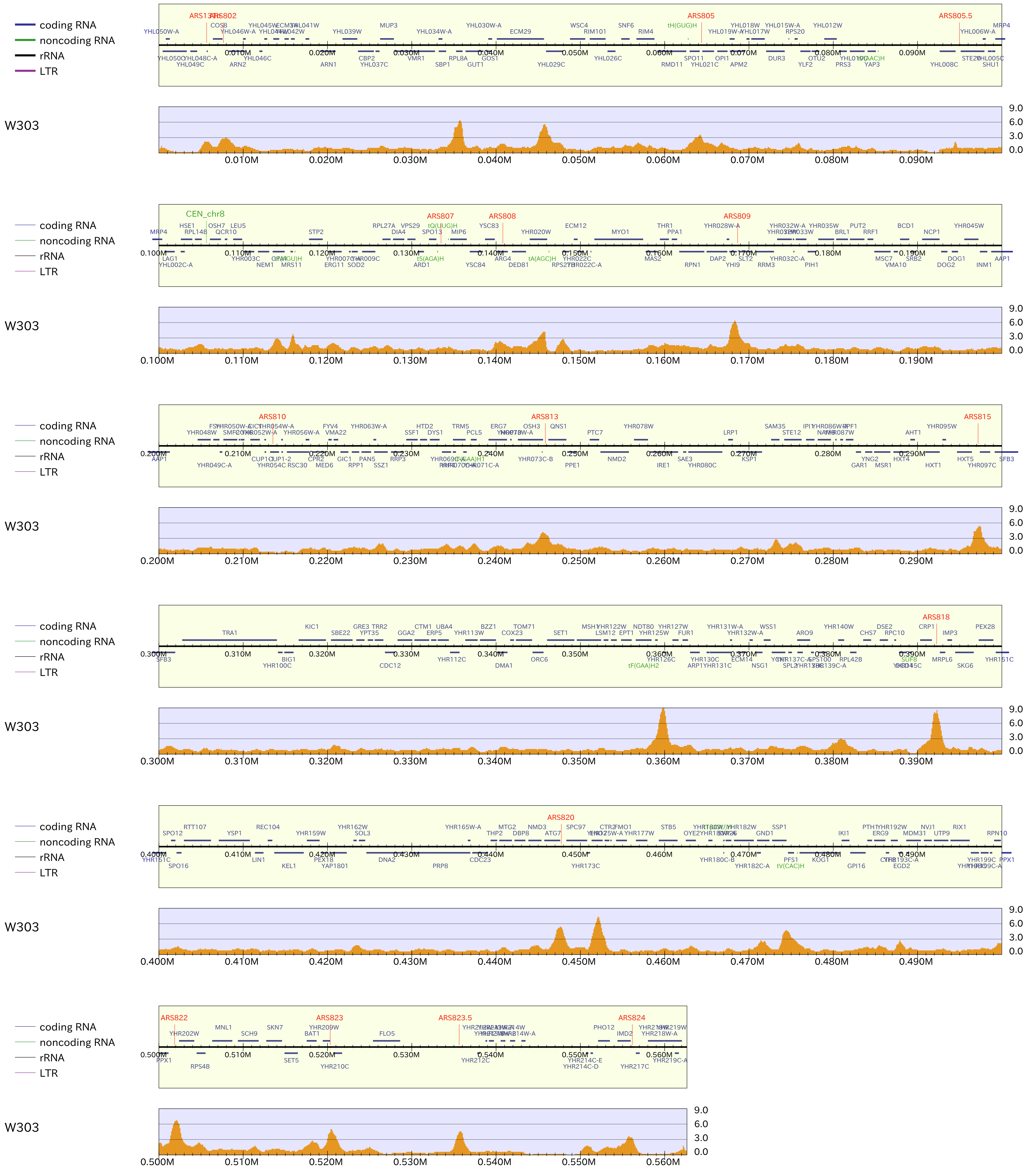

coding RNA
noncoding RNA
rRNA
LTR
W303
9.0
6.0
3.0
0.0
ARS1? ARS802
ARS805
ARS805.5
0.010M 0.020M 0.030M 0.040M 0.050M 0.060M 0.070M 0.080M 0.090M
CEN_chr8
ARS807
ARS808
ARS809
0.100M 0.110M 0.120M 0.130M 0.140M 0.150M 0.160M 0.170M 0.180M 0.190M
ARS810
ARS813
ARS815
0.200M 0.210M 0.220M 0.230M 0.240M 0.250M 0.260M 0.270M 0.280M 0.290M
ARS818
0.300M 0.310M 0.320M 0.330M 0.340M 0.350M 0.360M 0.370M 0.380M 0.390M
ARS820
0.400M 0.410M 0.420M 0.430M 0.440M 0.450M 0.460M 0.470M 0.480M 0.490M
ARS822
ARS823
ARS823.5
ARS824
0.500M 0.510M 0.520M 0.530M 0.540M 0.550M 0.560M

chrIX

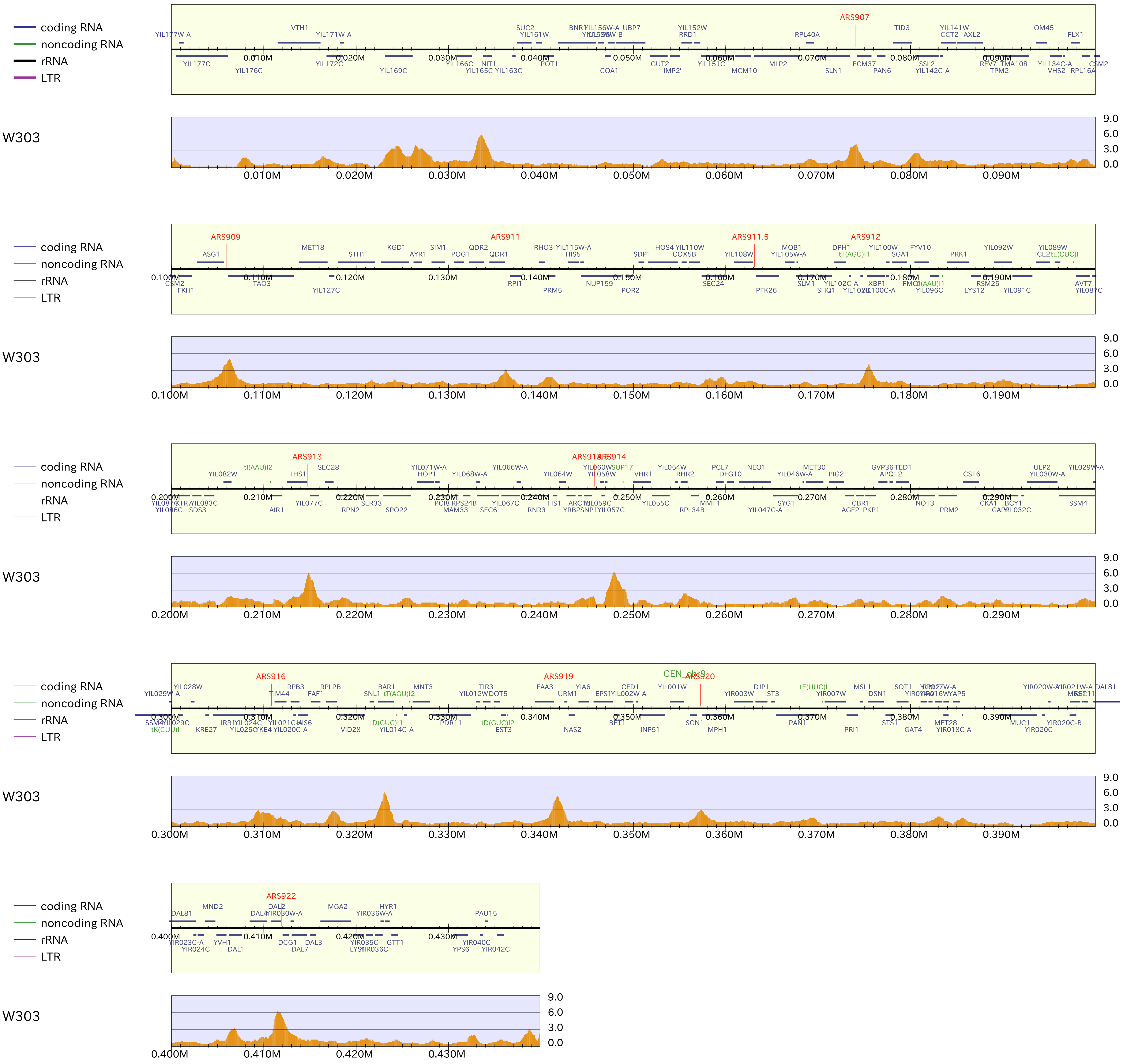
coding RNA
noncoding RNA
rRNA
LTR
W303
9.0
6.0
3.0
0.0
0.010M
0.020M
0.030M
0.040M
0.050M
0.060M
0.070M
0.080M
0.090M
0.100M
0.110M
0.120M
0.130M
0.140M
0.150M
0.160M
0.170M
0.180M
0.190M
0.200M
0.210M
0.220M
0.230M
0.240M
0.250M
0.260M
0.270M
0.280M
0.290M
0.300M
0.310M
0.320M
0.330M
0.340M
0.350M
0.360M
0.370M
0.380M
0.390M
0.400M
0.410M
0.420M
0.430M
ARS907
ARS909
ARS911
ARS911.5
ARS912
ARS913
ARS914
ARS916
ARS919
ARS920
ARS922
CEN_chr9

chrX_1

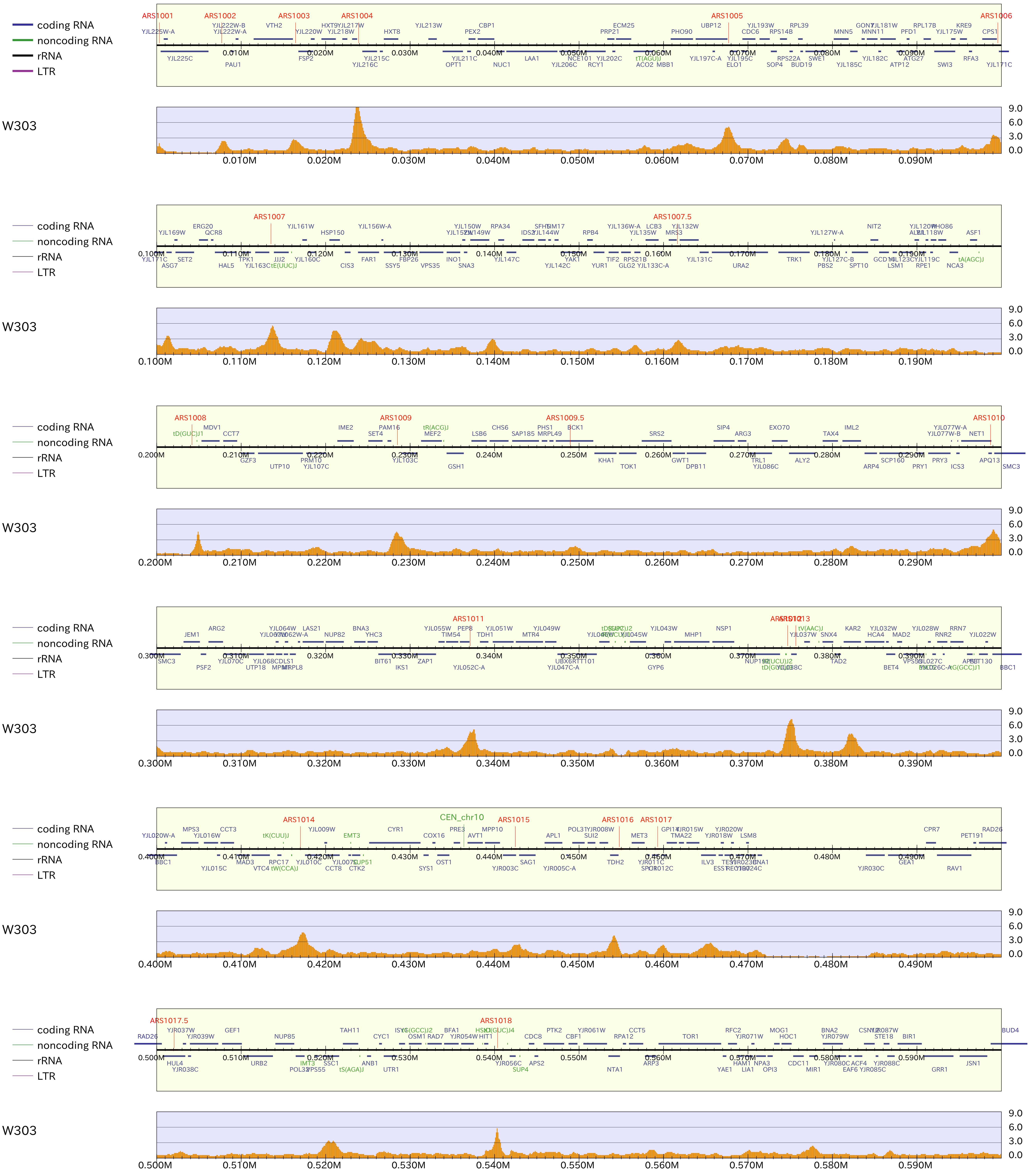

coding RNA
noncoding RNA
rRNA
LTR
W303
ARS1001 ARS1002 ARS1003 ARS1004 ARS1005 ARS1006
9.0 6.0 3.0 0.0
0.010M 0.020M 0.030M 0.040M 0.050M 0.060M 0.070M 0.080M 0.090M
ARS1007 ARS1007.5
0.100M 0.110M 0.120M 0.130M 0.140M 0.150M 0.160M 0.170M 0.180M 0.190M
ARS1008 ARS1009 ARS1009.5 ARS1010
0.200M 0.210M 0.220M 0.230M 0.240M 0.250M 0.260M 0.270M 0.280M 0.290M
ARS1011
0.300M 0.310M 0.320M 0.330M 0.340M 0.350M 0.360M 0.370M 0.380M 0.390M
ARS1014 CEN_chr10 ARS1015 ARS1016 ARS1017
0.400M 0.410M 0.420M 0.430M 0.440M 0.450M 0.460M 0.470M 0.480M 0.490M
ARS1017.5 ARS1018
0.500M 0.510M 0.520M 0.530M 0.540M 0.550M 0.560M 0.570M 0.580M 0.590M

chrX_2

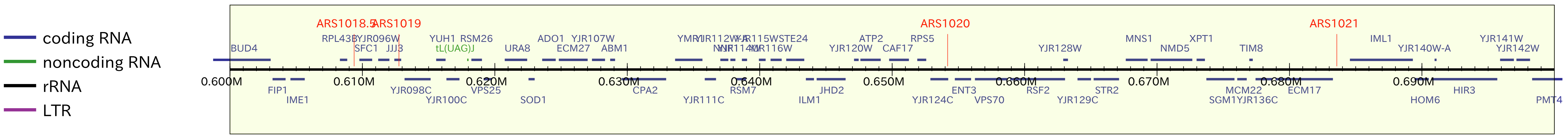

W303

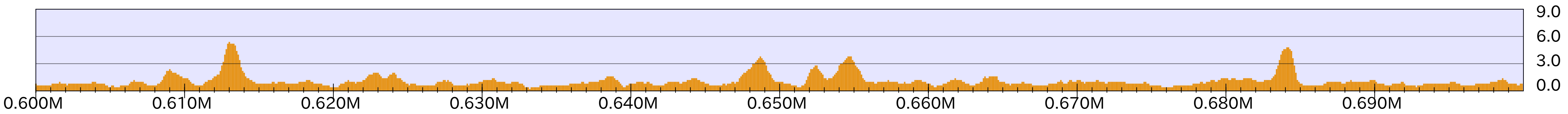

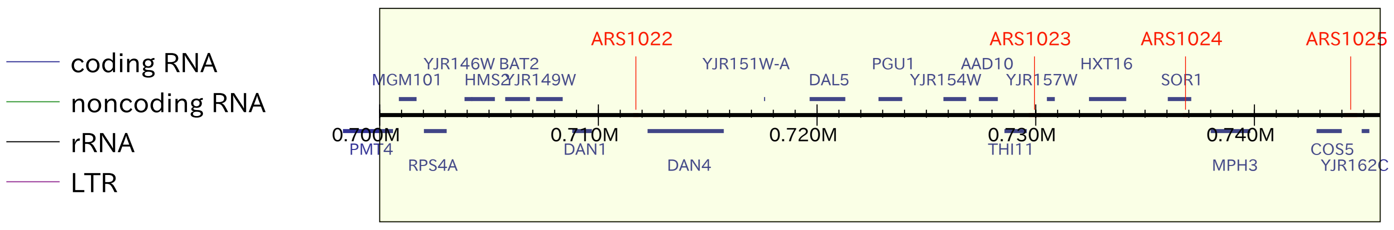

W303

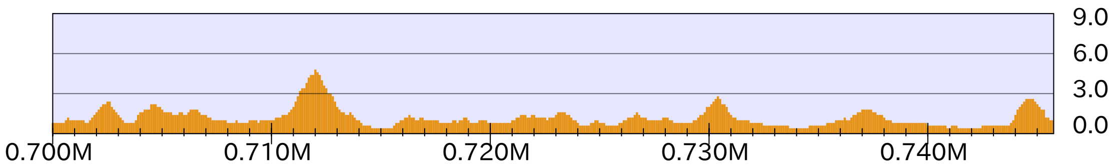

chrXI_1

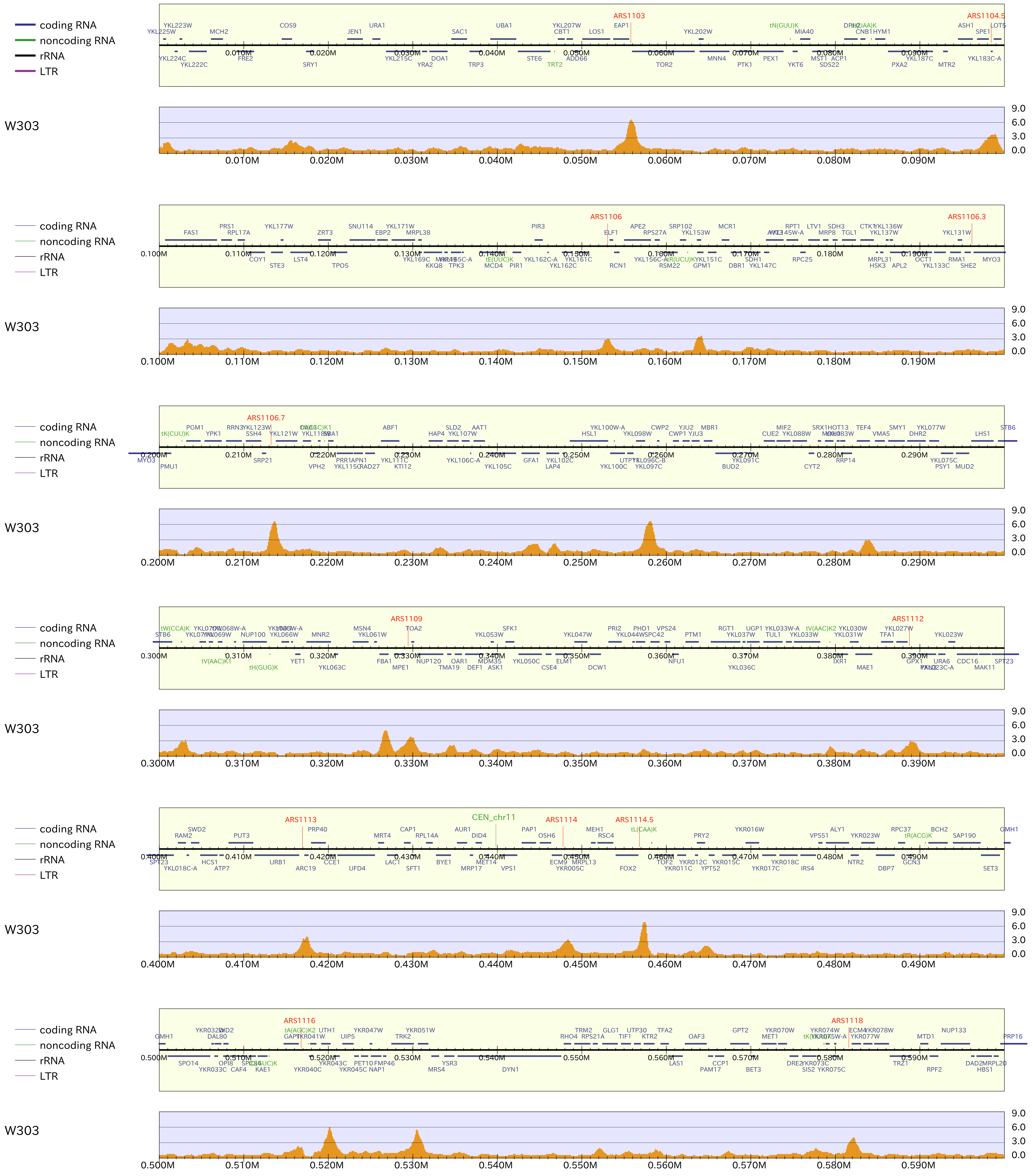
coding RNA
noncoding RNA
rRNA
LTR
W303
9.0
6.0
3.0
0.0
ARS1103
ARS1104.5
0.010M
0.020M
0.030M
0.040M
0.050M
0.060M
0.070M
0.080M
0.090M
ARS1106
ARS1106.3
0.100M
0.110M
0.120M
0.130M
0.140M
0.150M
0.160M
0.170M
0.180M
0.190M
ARS1106.7
0.200M
0.210M
0.220M
0.230M
0.240M
0.250M
0.260M
0.270M
0.280M
0.290M
ARS1109
ARS1112
0.300M
0.310M
0.320M
0.330M
0.340M
0.350M
0.360M
0.370M
0.380M
0.390M
ARS1113
CEN_chr11
ARS1114
ARS1114.5
0.400M
0.410M
0.420M
0.430M
0.440M
0.450M
0.460M
0.470M
0.480M
0.490M
ARS1116
ARS1118
0.500M
0.510M
0.520M
0.530M
0.540M
0.550M
0.560M
0.570M
0.580M
0.590M

chrXI_2

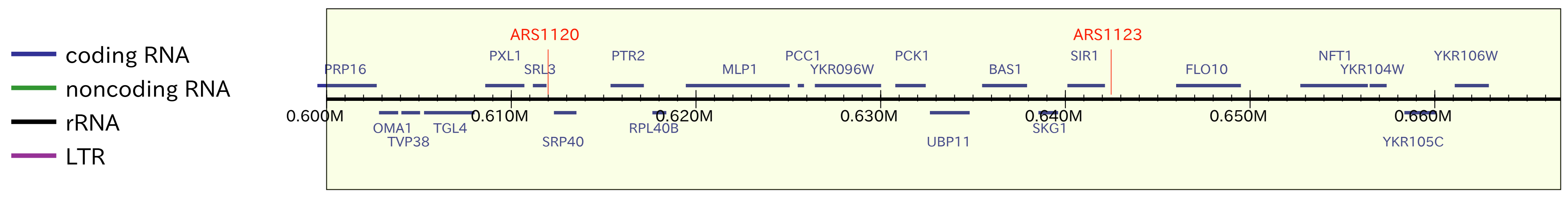

coding RNA
noncoding RNA
rRNA
LTR
ARS1120
ARS1123
PRP16
PXL1
SRL3
PTR2
MLP1
PCC1
YKR096W
PCK1
BAS1
SIR1
FLO10
NFT1
YKR104W
YKR106W
0.600M
0.610M
0.620M
0.630M
0.640M
0.650M
0.660M
OMA1
TVP38
TGL4
SRP40
RPL40B
UBP11
SKG1
YKR105C

W303

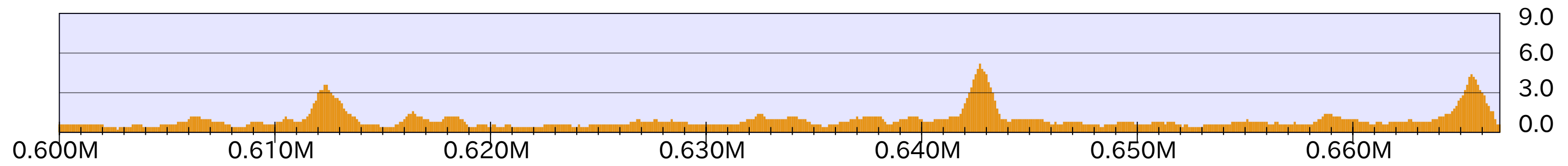

9.0
6.0
3.0
0.0
0.600M
0.610M
0.620M
0.630M
0.640M
0.650M
0.660M

chrXII_1

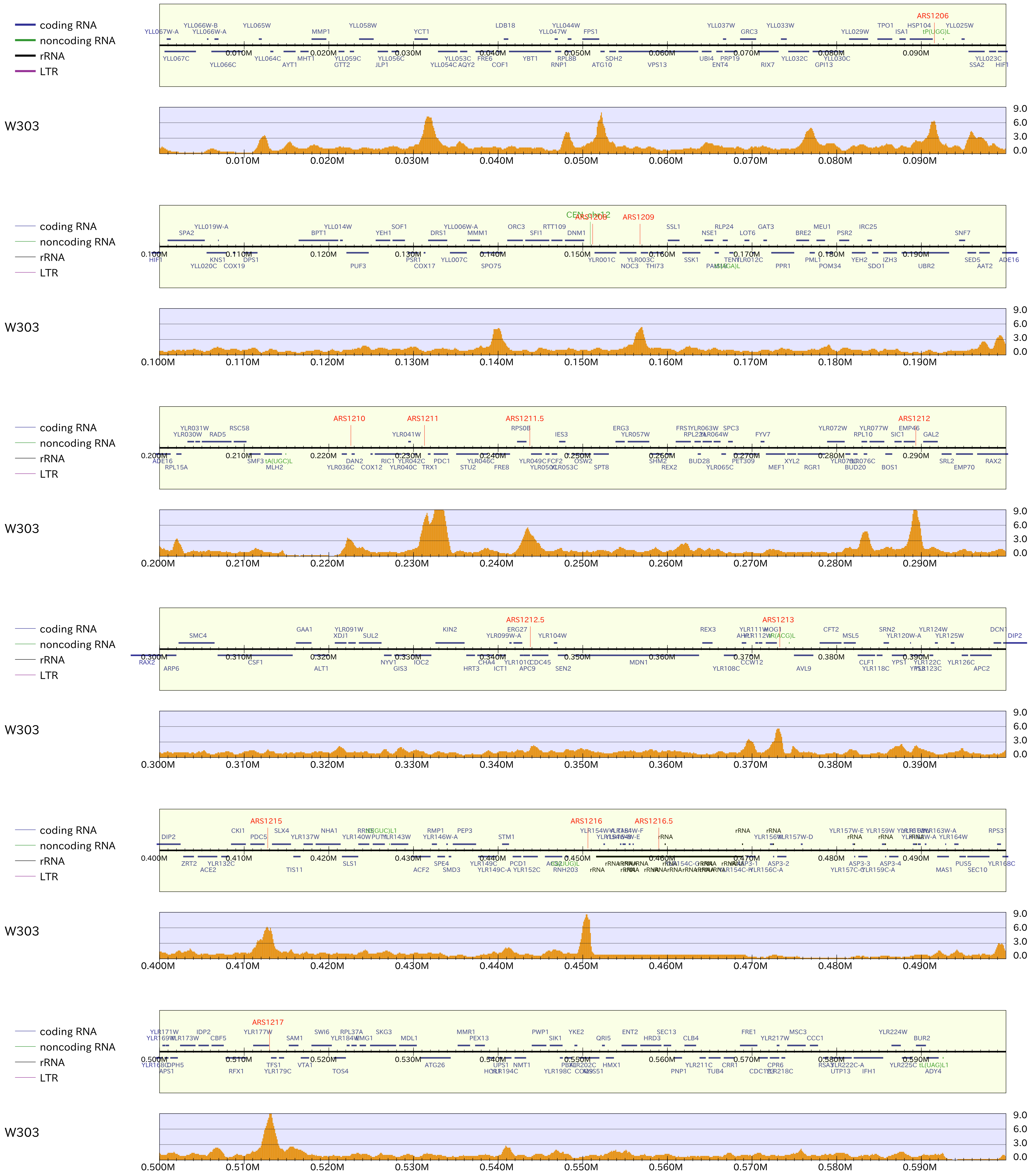
coding RNA
noncoding RNA
rRNA
LTR
W303
9.0
6.0
3.0
0.0
0.010M
0.020M
0.030M
0.040M
0.050M
0.060M
0.070M
0.080M
0.090M
0.100M
0.110M
0.120M
0.130M
0.140M
0.150M
0.160M
0.170M
0.180M
0.190M
0.200M
0.210M
0.220M
0.230M
0.240M
0.250M
0.260M
0.270M
0.280M
0.290M
0.300M
0.310M
0.320M
0.330M
0.340M
0.350M
0.360M
0.370M
0.380M
0.390M
0.400M
0.410M
0.420M
0.430M
0.440M
0.450M
0.460M
0.470M
0.480M
0.490M
0.500M
0.510M
0.520M
0.530M
0.540M
0.550M
0.560M
0.570M
0.580M
0.590M

chrXII_2

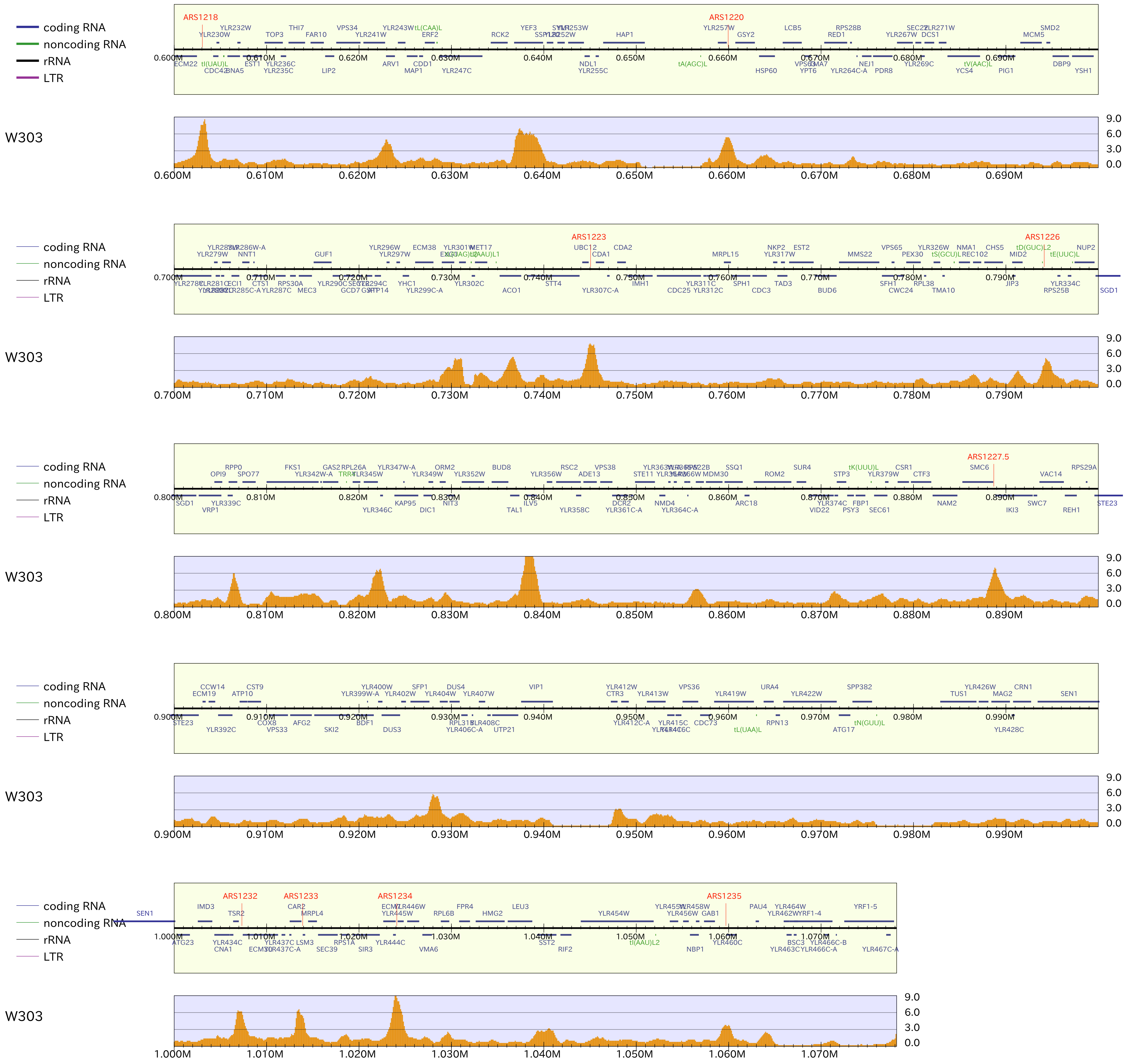

coding RNA
noncoding RNA
rRNA
LTR
W303
ARS1218
ARS1220
ARS1223
ARS1226
ARS1227.5
ARS1232
ARS1233
ARS1234
ARS1235
0.600M 0.610M 0.620M 0.630M 0.640M 0.650M 0.660M 0.670M 0.680M 0.690M
0.700M 0.710M 0.720M 0.730M 0.740M 0.750M 0.760M 0.770M 0.780M 0.790M
0.800M 0.810M 0.820M 0.830M 0.840M 0.850M 0.860M 0.870M 0.880M 0.890M
0.900M 0.910M 0.920M 0.930M 0.940M 0.950M 0.960M 0.970M 0.980M 0.990M
1.000M 1.010M 1.020M 1.030M 1.040M 1.050M 1.060M 1.070M
9.0
6.0
3.0
0.0

chrXIII_1

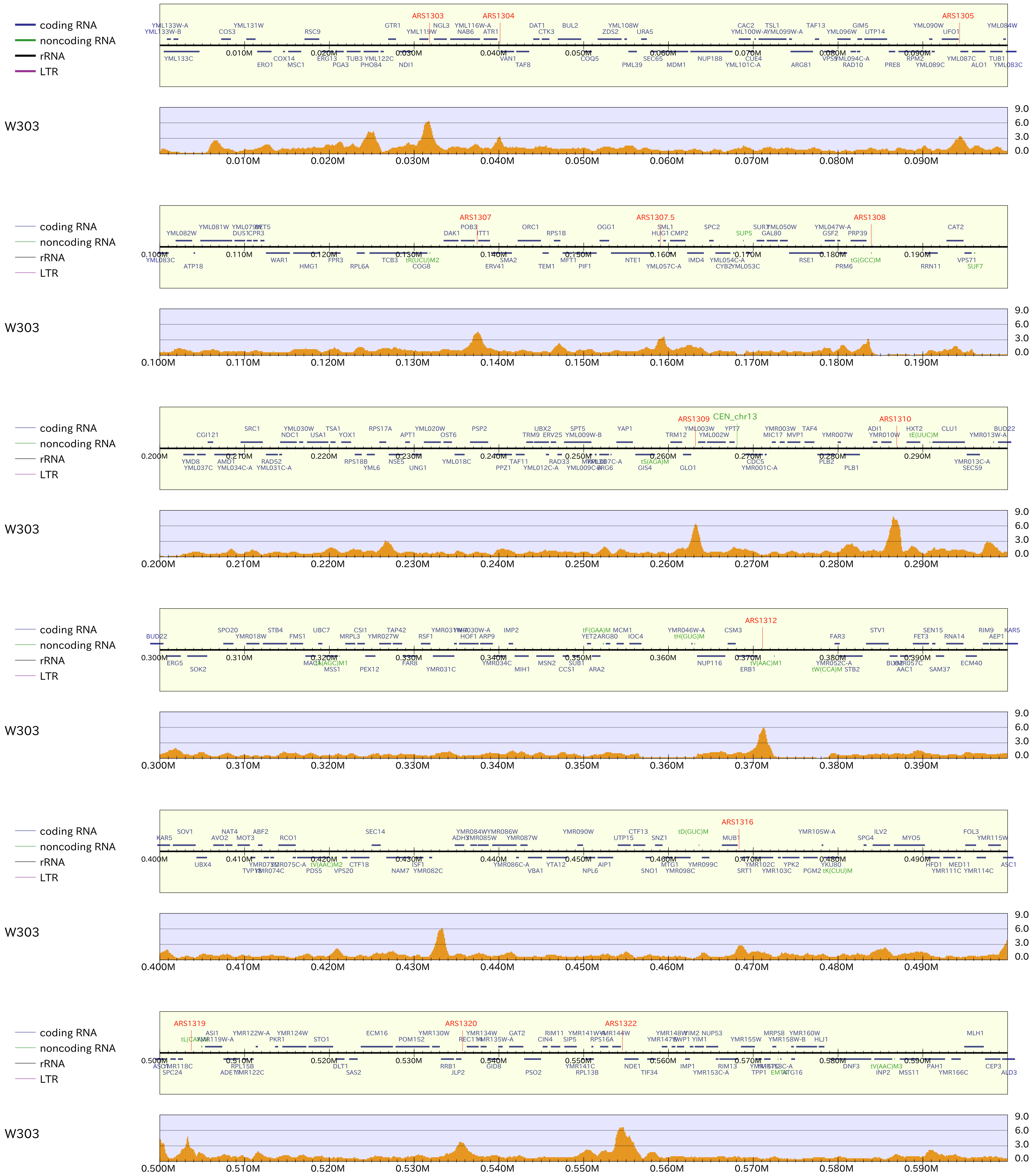
coding RNA
noncoding RNA
rRNA
LTR
W303
9.0
6.0
3.0
0.0
ARS1303
ARS1304
ARS1305
0.010M 0.020M 0.030M 0.040M 0.050M 0.060M 0.070M 0.080M 0.090M
ARS1307
ARS1307.5
ARS1308
0.100M 0.110M 0.120M 0.130M 0.140M 0.150M 0.160M 0.170M 0.180M 0.190M
ARS1309
CEN_chr13
ARS1310
0.200M 0.210M 0.220M 0.230M 0.240M 0.250M 0.260M 0.270M 0.280M 0.290M
ARS1312
0.300M 0.310M 0.320M 0.330M 0.340M 0.350M 0.360M 0.370M 0.380M 0.390M
ARS1316
0.400M 0.410M 0.420M 0.430M 0.440M 0.450M 0.460M 0.470M 0.480M 0.490M
ARS1319
ARS1320
ARS1322
0.500M 0.510M 0.520M 0.530M 0.540M 0.550M 0.560M 0.570M 0.580M 0.590M

chrXIII_2

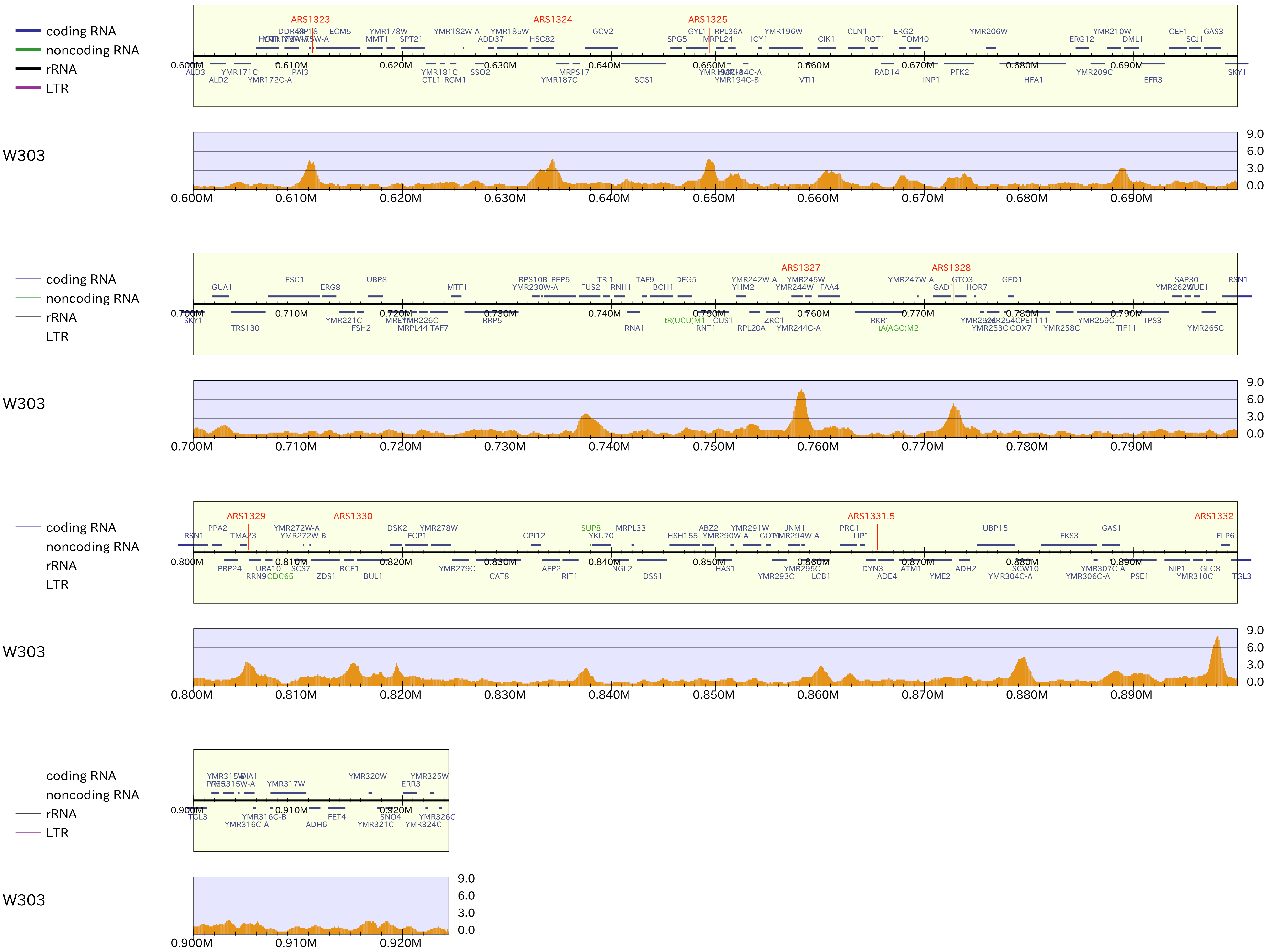

coding RNA
noncoding RNA
rRNA
LTR
ARS1323
ARS1324
ARS1325
ECM5
YMR178W
YMR182W-A
YMR185W
GCV2
GYL1
RPL36A
YMR196W
CLN1
ERG2
YMR206W
YMR210W
CEF1
GAS3
MMT1
SPT21
ADD37
HSC82
SPG5
MRPL24
ICY1
CIK1
ROT1
TOM40
ERG12
DML1
SCJ1
0.600M
0.610M
0.620M
0.630M
0.640M
0.650M
0.660M
0.670M
0.680M
0.690M
ALD3
ALD2
YMR171C
YMR172C-A
PAI3
YMR181C
CTL1
RGM1
SSO2
MRPS17
YMR187C
SGS1
YMR194C-B
VTI1
RAD14
INP1
PFK2
HFA1
YMR209C
EFR3
SKY1
W303
9.0
6.0
3.0
0.0
ARS1327
ARS1328
GUA1
ESC1
ERG8
UBP8
MTF1
RPS10B
PEP5
YMR230W-A
TRI1
FUS2
RNH1
TAF9
BCH1
DFG5
YMR242W-A
YHM2
YMR245W
YMR244W
FAA4
YMR247W-A
GTO3
GAD1
HOR7
GFD1
SAP30
RSN1
0.700M
0.710M
0.720M
0.730M
0.740M
0.750M
0.760M
0.770M
0.780M
0.790M
SKY1
TRS130
YMR221C
FSH2
YMR226C
MRPL44
TAF7
RRP5
RNA1
tR(UCU)M1
CUS1
RNT1
RPL20A
ZRC1
YMR244C-A
RKR1
tA(AGC)M2
YMR253C
COX7
YMR258C
YMR259C
TIF11
TPS3
YMR265C
ARS1329
ARS1330
ARS1331.5
ARS1332
RSN1
PPA2
TMA23
YMR272W-A
YMR272W-B
DSK2
FCP1
YMR278W
GPI12
SUP8
YKU70
MRPL33
HSH155
ABZ2
YMR290W-A
YMR291W
JNM1
YMR294W-A
PRC1
LIP1
UBP15
FKS3
GAS1
ELP6
0.800M
0.810M
0.820M
0.830M
0.840M
0.850M
0.860M
0.870M
0.880M
0.890M
PRP24
URA10
RRN9
CDC65
SCS7
ZDS1
RCE1
BUL1
YMR279C
CAT8
AEP2
RIT1
NGL2
DSS1
HAS1
YMR293C
YMR295C
LCB1
DYN3
ADE4
ATM1
YME2
ADH2
SCW10
YMR304C-A
YMR306C-A
YMR307C-A
PSE1
NIP1
YMR310C
GLC8
TGL3
YMR317W
YMR320W
YMR325W
ERR3
0.900M
0.910M
0.920M
TGL3
YMR316C-B
YMR316C-A
ADH6
FET4
SNO4
YMR321C
YMR324C
YMR326C

chrXIV_1

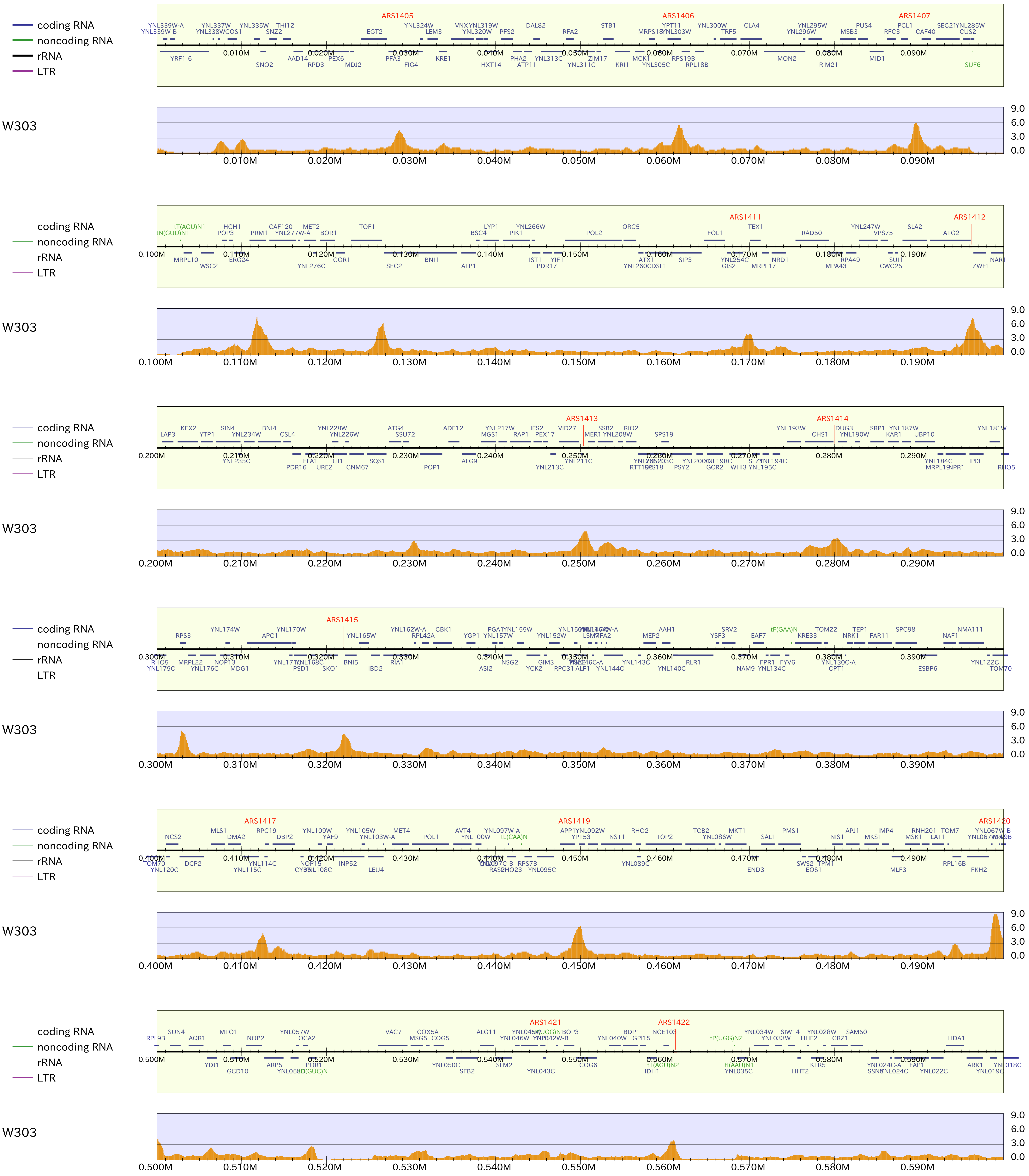

coding RNA
noncoding RNA
rRNA
LTR
W303
ARS1405
ARS1406
ARS1407
ARS1411
ARS1412
ARS1413
ARS1414
ARS1415
ARS1417
ARS1419
ARS1420
ARS1421
ARS1422
9.0
6.0
3.0
0.0
0.010M
0.020M
0.030M
0.040M
0.050M
0.060M
0.070M
0.080M
0.090M
0.100M
0.110M
0.120M
0.130M
0.140M
0.150M
0.160M
0.170M
0.180M
0.190M
0.200M
0.210M
0.220M
0.230M
0.240M
0.250M
0.260M
0.270M
0.280M
0.290M
0.300M
0.310M
0.320M
0.330M
0.340M
0.350M
0.360M
0.370M
0.380M
0.390M
0.400M
0.410M
0.420M
0.430M
0.440M
0.450M
0.460M
0.470M
0.480M
0.490M
0.500M
0.510M
0.520M
0.530M
0.540M
0.550M
0.560M
0.570M
0.580M
0.590M

chrXIV_2

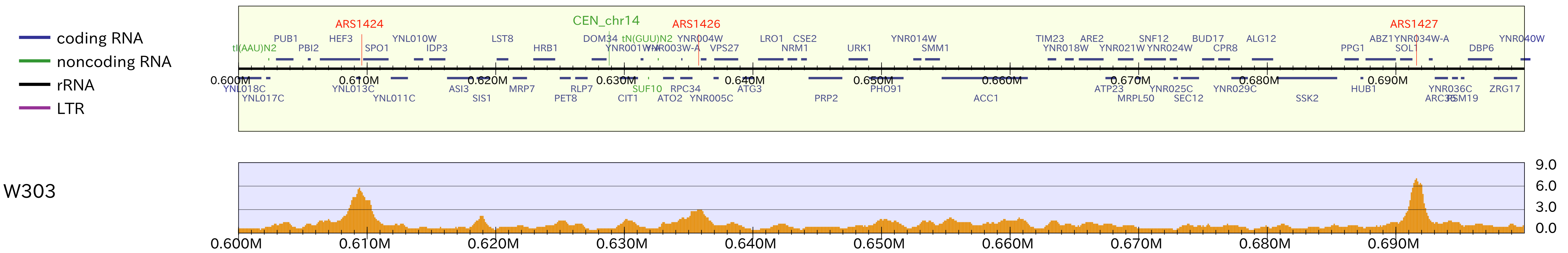

coding RNA
noncoding RNA
rRNA
LTR
ARS1424
CEN_chr14
ARS1426
ARS1427
W303
9.0
6.0
3.0
0.0
0.600M
0.610M
0.620M
0.630M
0.640M
0.650M
0.660M
0.670M
0.680M
0.690M

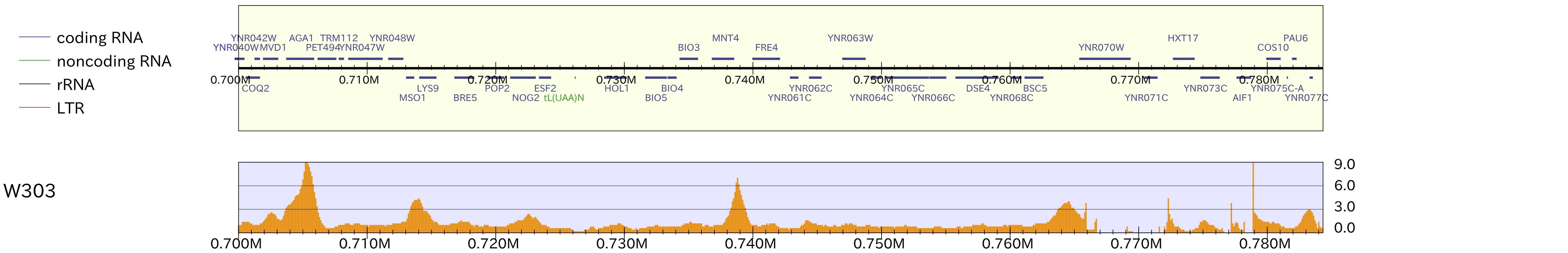

coding RNA
noncoding RNA
rRNA
LTR
W303
9.0
6.0
3.0
0.0
0.700M
0.710M
0.720M
0.730M
0.740M
0.750M
0.760M
0.770M
0.780M

chrXV_1

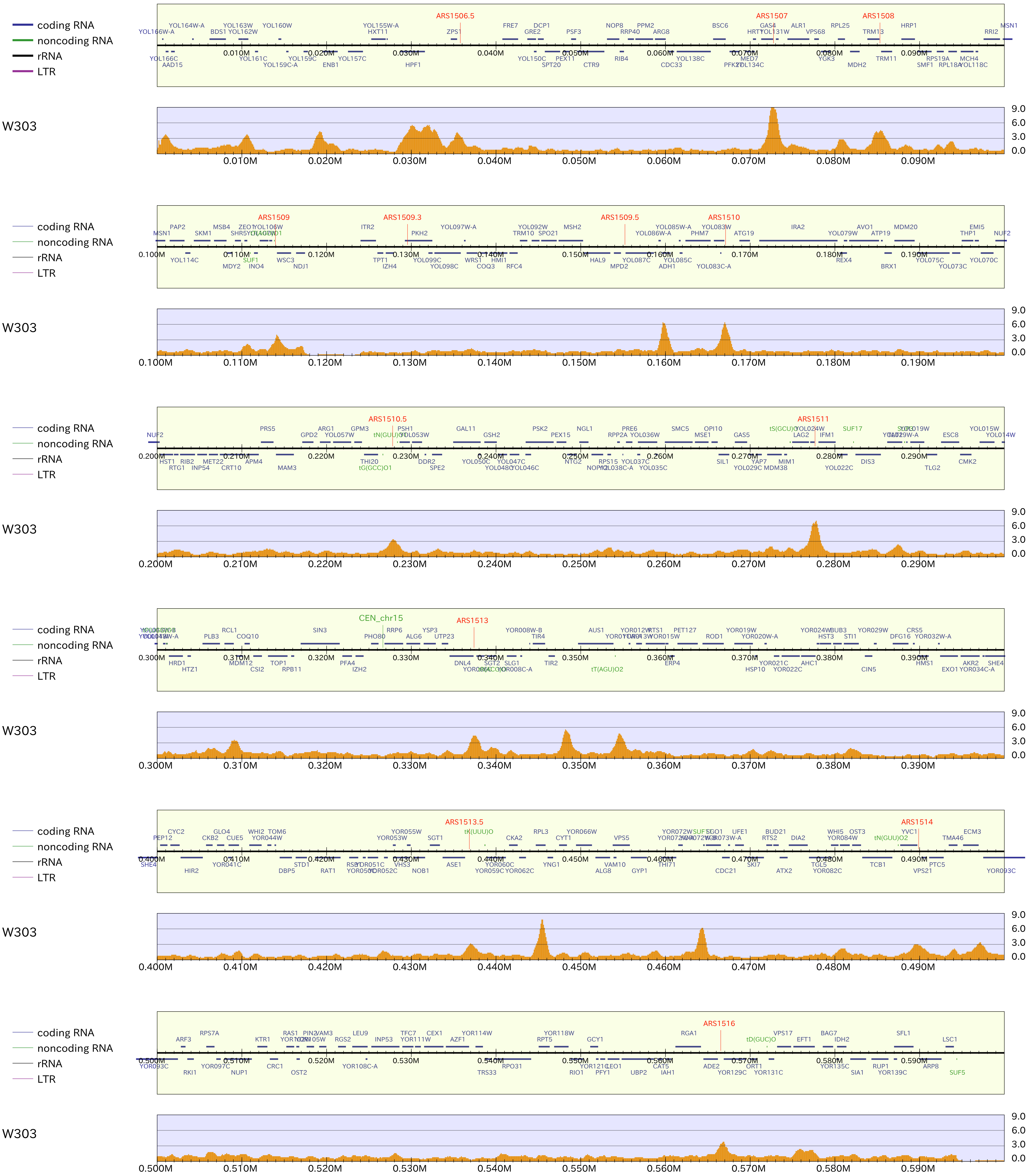
coding RNA
noncoding RNA
rRNA
LTR
W303
9.0
6.0
3.0
0.0
ARS1506.5
ARS1507
ARS1508
ARS1509
ARS1509.3
ARS1509.5
ARS1510
ARS1510.5
ARS1511
CEN_chr15
ARS1513
ARS1513.5
ARS1514
ARS1516
0.010M 0.020M 0.030M 0.040M 0.050M 0.060M 0.070M 0.080M 0.090M
0.100M 0.110M 0.120M 0.130M 0.140M 0.150M 0.160M 0.170M 0.180M 0.190M
0.200M 0.210M 0.220M 0.230M 0.240M 0.250M 0.260M 0.270M 0.280M 0.290M
0.300M 0.310M 0.320M 0.330M 0.340M 0.350M 0.360M 0.370M 0.380M 0.390M
0.400M 0.410M 0.420M 0.430M 0.440M 0.450M 0.460M 0.470M 0.480M 0.490M
0.500M 0.510M 0.520M 0.530M 0.540M 0.550M 0.560M 0.570M 0.580M 0.590M

chrXV_2

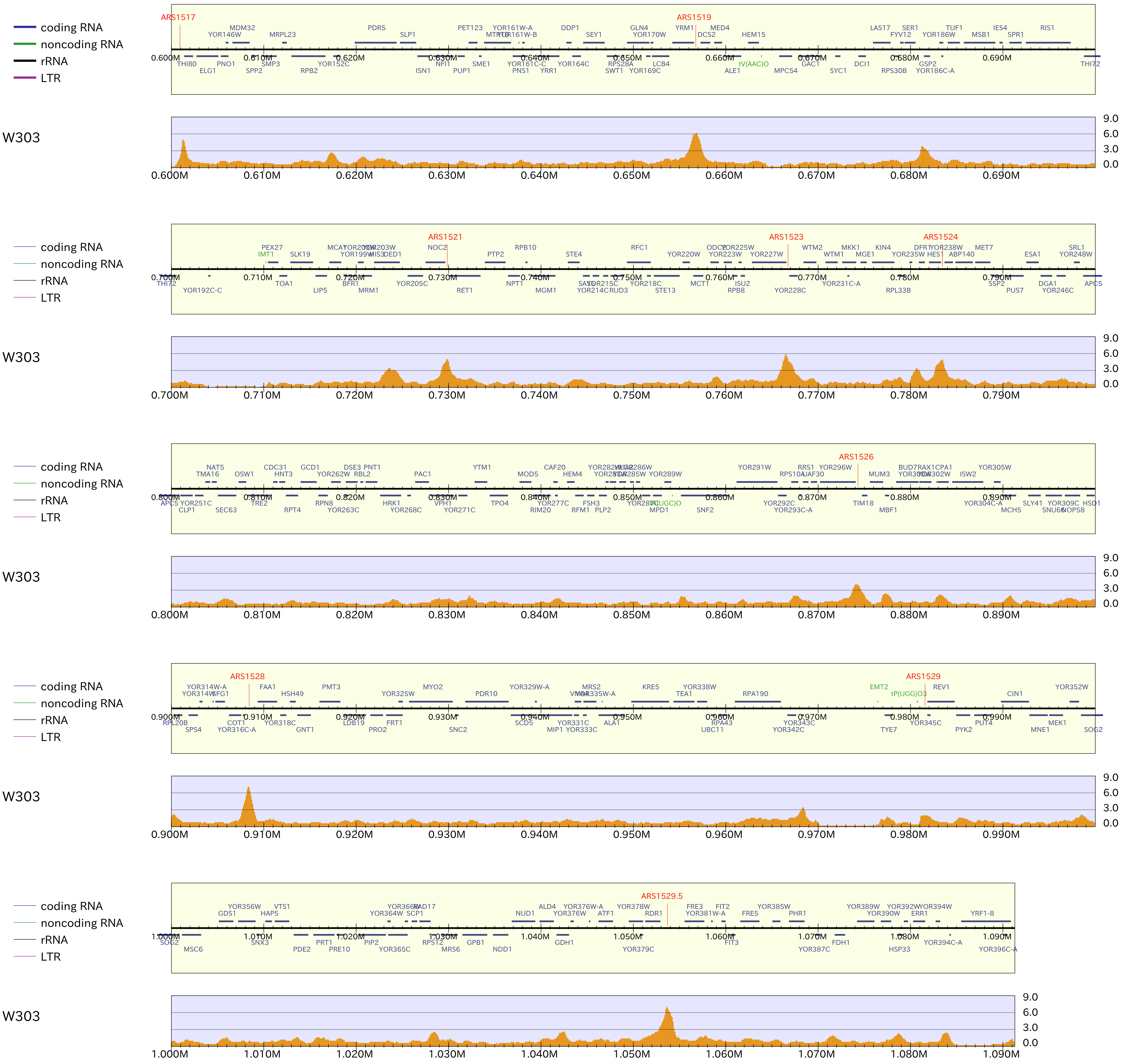

coding RNA
noncoding RNA
rRNA
LTR
W303
ARS1517
ARS1519
ARS1521
ARS1523
ARS1524
ARS1526
ARS1528
ARS1529
ARS1529.5
9.0
6.0
3.0
0.0
0.600M
0.610M
0.620M
0.630M
0.640M
0.650M
0.660M
0.670M
0.680M
0.690M
0.700M
0.710M
0.720M
0.730M
0.740M
0.750M
0.760M
0.770M
0.780M
0.790M
0.800M
0.810M
0.820M
0.830M
0.840M
0.850M
0.860M
0.870M
0.880M
0.890M
0.900M
0.910M
0.920M
0.930M
0.940M
0.950M
0.960M
0.970M
0.980M
0.990M
1.000M
1.010M
1.020M
1.030M
1.040M
1.050M
1.060M
1.070M
1.080M
1.090M

chrXVI_1

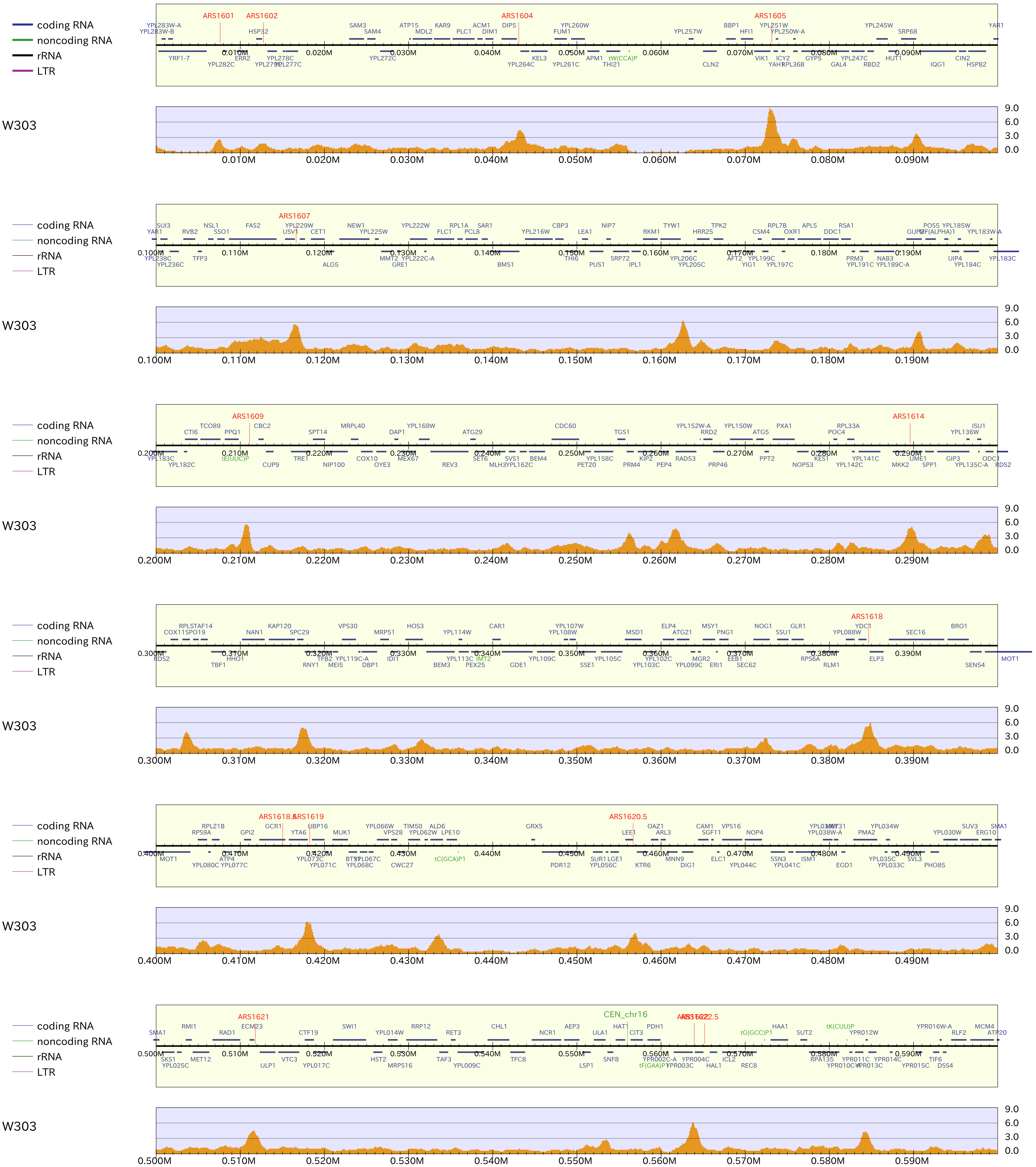
coding RNA
noncoding RNA
rRNA
LTR
W303
9.0
6.0
3.0
0.0
ARS1601
ARS1602
ARS1604
ARS1605
ARS1607
ARS1609
ARS1614
ARS1618
ARS1618.5
ARS1619
ARS1620.5
ARS1621
CEN_chr16
0.010M
0.020M
0.030M
0.040M
0.050M
0.060M
0.070M
0.080M
0.090M
0.100M
0.110M
0.120M
0.130M
0.140M
0.150M
0.160M
0.170M
0.180M
0.190M
0.200M
0.210M
0.220M
0.230M
0.240M
0.250M
0.260M
0.270M
0.280M
0.290M
0.300M
0.310M
0.320M
0.330M
0.340M
0.350M
0.360M
0.370M
0.380M
0.390M
0.400M
0.410M
0.420M
0.430M
0.440M
0.450M
0.460M
0.470M
0.480M
0.490M
0.500M
0.510M
0.520M
0.530M
0.540M
0.550M
0.560M
0.570M
0.580M
0.590M

chrXVI_2

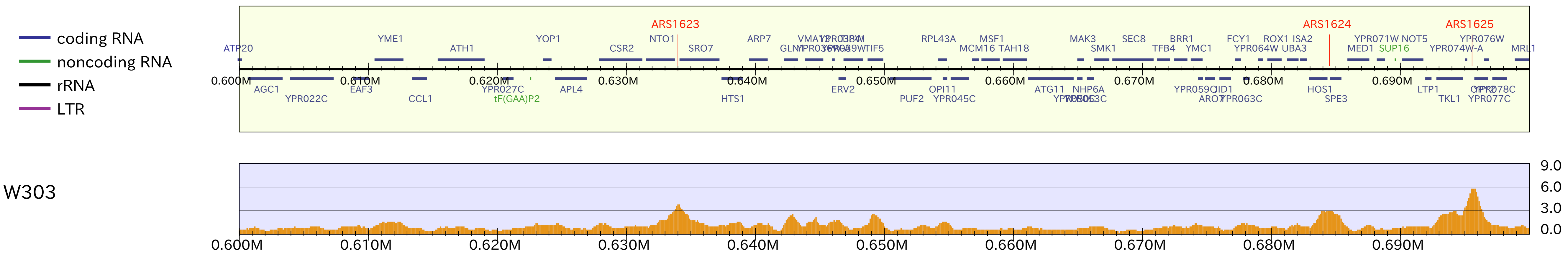
coding RNA
noncoding RNA
rRNA
LTR
ARS1623
ARS1624
ARS1625
ATP20 YME1 ATH1 YOP1 CSR2 NTO1 SRO7 ARP7 VMA13 GLN1 ERV2 RPL43A MSF1 MCM16 TAH18 MAK3 SMK1 SEC8 TFB4 BRR1 YMC1 FCY1 YPR064W ROX1 ISA2 UBA3 YPR071W NOT5 MED1 SUP16 YPR074W-A YPR076W MRL1
AGC1 YPR022C EAF3 CCL1 YPR027C tF(GAA)P2 APL4 HTS1 PUF2 OPI11 YPR045C ATG11 NHP6A AROT YPR063C HOS1 SPE3 LTP1 TKL1 YPR077C
0.600M 0.610M 0.620M 0.630M 0.640M 0.650M 0.660M 0.670M 0.680M 0.690M
W303
9.0
6.0
3.0
0.0

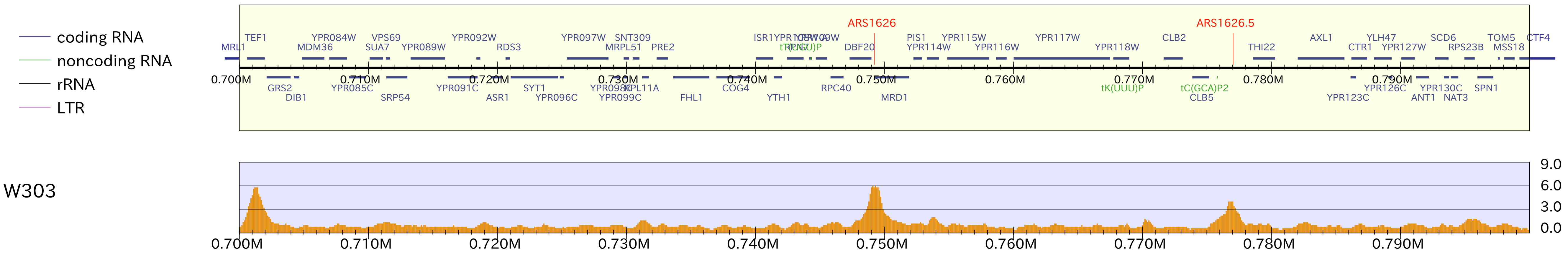
coding RNA
noncoding RNA
rRNA
LTR
ARS1626
ARS1626.5
MRL1 TEF1 MDM36 YPR084W VPS69 SUA7 YPR089W YPR092W RDS3 YPR097W SNT309 MRPL51 PRE2 DBF20 PIS1 YPR114W YPR115W YPR116W YPR117W YPR118W CLB2 THI22 AXL1 CTR1 YLH47 YPR127W SCD6 RPS23B TOM5 MSS18 CTF4
GRS2 DIB1 YPR085C SRP54 YPR091C ASR1 SYT1 YPR096C YPR099C FHL1 COG4 YTH1 RPC40 MRD1 tK(UUU)P tC(GCA)P2 CLB5 YPR123C YPR126C ANT1 YPR130C NAT3 SPN1
0.700M 0.710M 0.720M 0.730M 0.740M 0.750M 0.760M 0.770M 0.780M 0.790M
W303
9.0
6.0
3.0
0.0

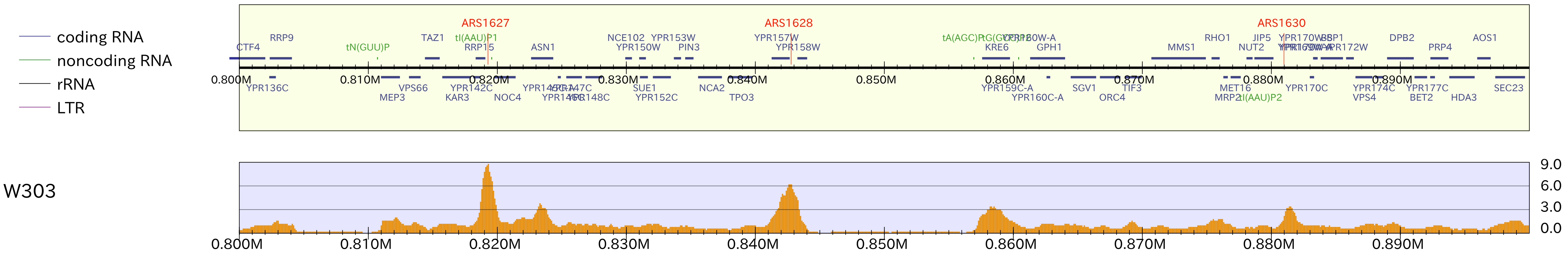
coding RNA
noncoding RNA
rRNA
LTR
ARS1627
ARS1628
ARS1630
CTF4 RRP9 tN(GUU)P TAZ1 tI(AAU)P1 RRP15 ASN1 NCE102 YPR150W YPR153W PIN3 YPR157W YPR158W KRE6 GPH1 MMS1 RHO1 NUT2 JIP5 DPB2 PRP4 AOS1
YPR136C MEP3 VPS66 KAR3 YPR142C NOC4 YPR148C SUE1 YPR152C NCA2 TPO3 YPR159C-A YPR160C-A SGV1 ORC4 TIF3 MET16 MRP21 tI(AAU)P2 YPR170C YPR174C VPS4 YPR177C BET2 HDA3 SEC23
0.800M 0.810M 0.820M 0.830M 0.840M 0.850M 0.860M 0.870M 0.880M 0.890M
W303
9.0
6.0
3.0
0.0

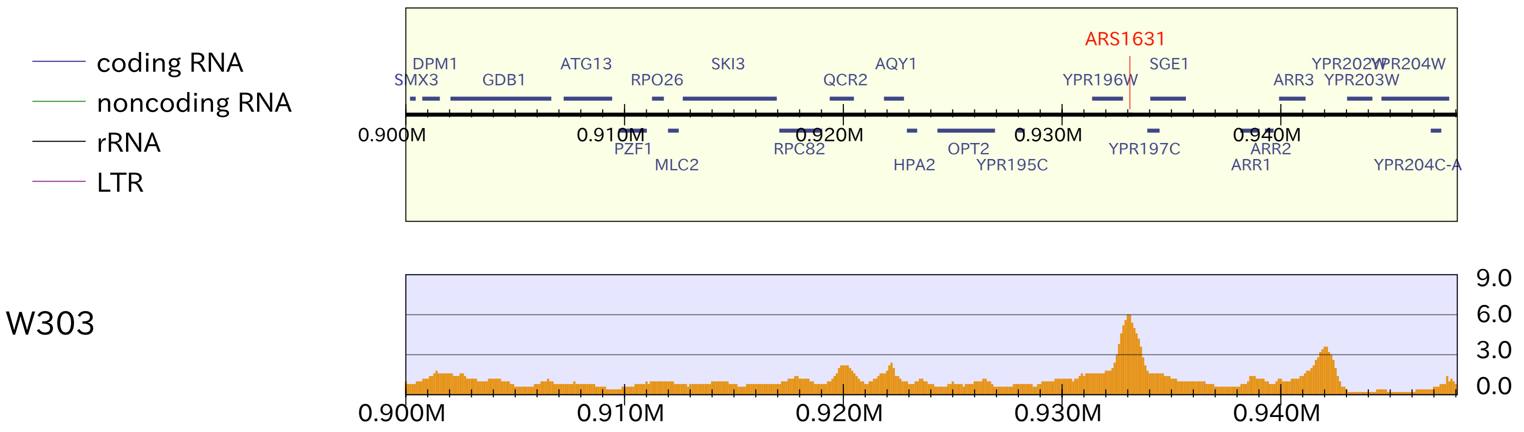
coding RNA
noncoding RNA
rRNA
LTR
ARS1631
DPM1 SMX3 GDB1 ATG13 RPO26 SKI3 QCR2 AQY1 YPR196W SGE1 ARR3 YPR202W YPR203W YPR204W
PZF1 MLC2 RPC82 HPA2 OPT2 YPR195C YPR197C ARR2 ARR1 YPR204C-A
0.900M 0.910M 0.920M 0.930M 0.940M
W303
9.0
6.0
3.0
0.0

chrM

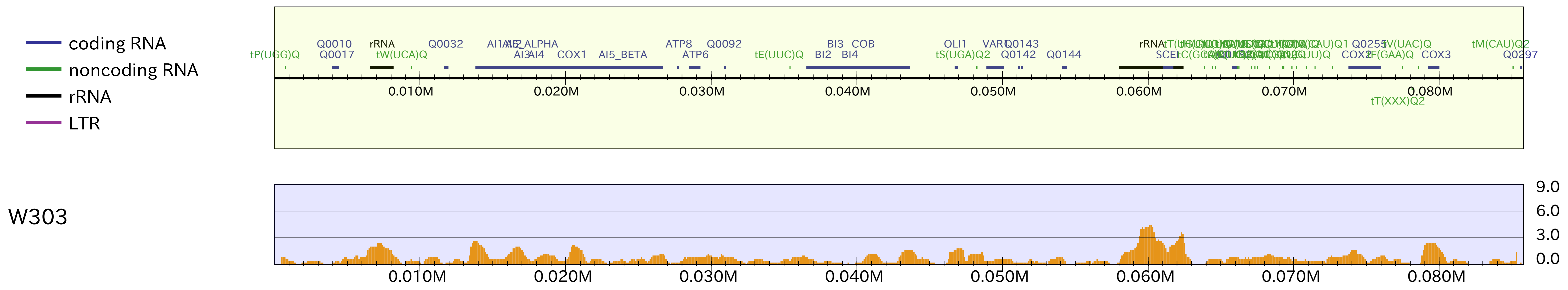
coding RNA
noncoding RNA
rRNA
LTR
W303
0.010M
0.020M
0.030M
0.040M
0.050M
0.060M
0.070M
0.080M
9.0
6.0
3.0
0.0

chr2micron

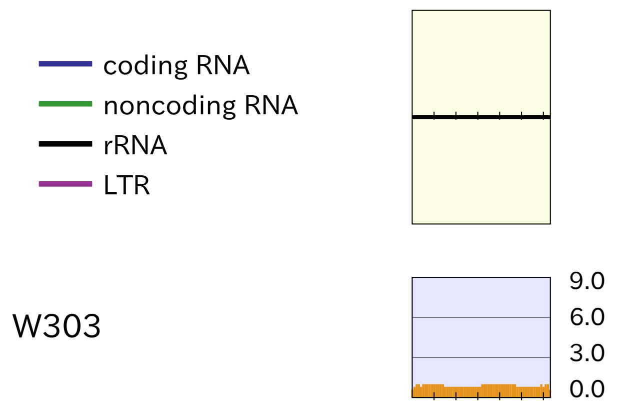

Müller, Hawkins, et al. Figure S3

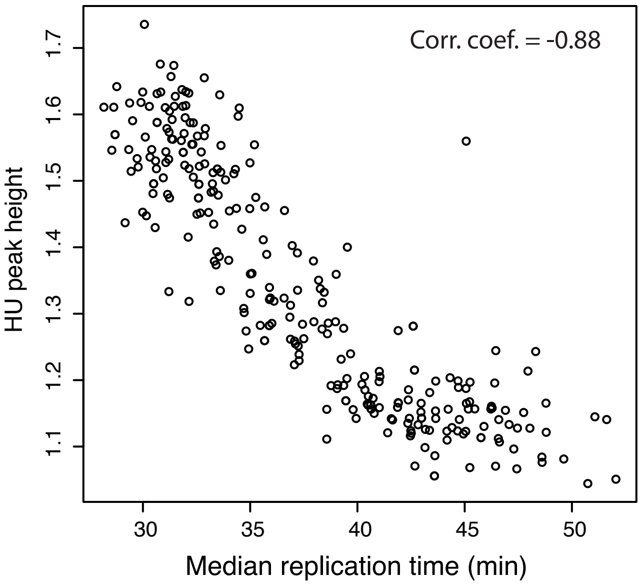

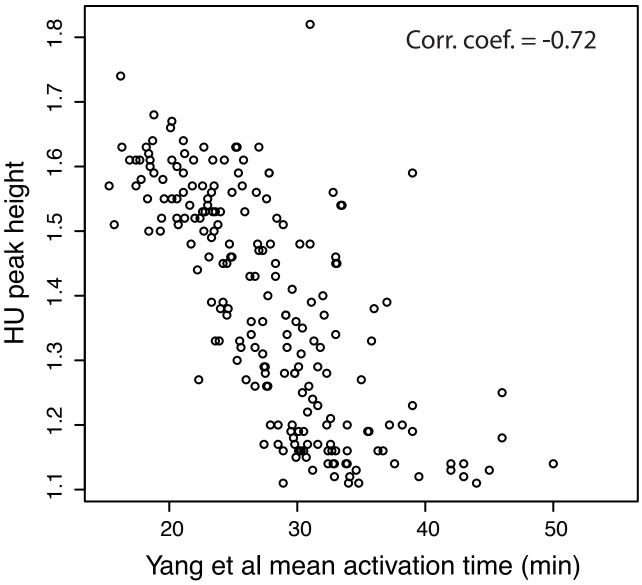

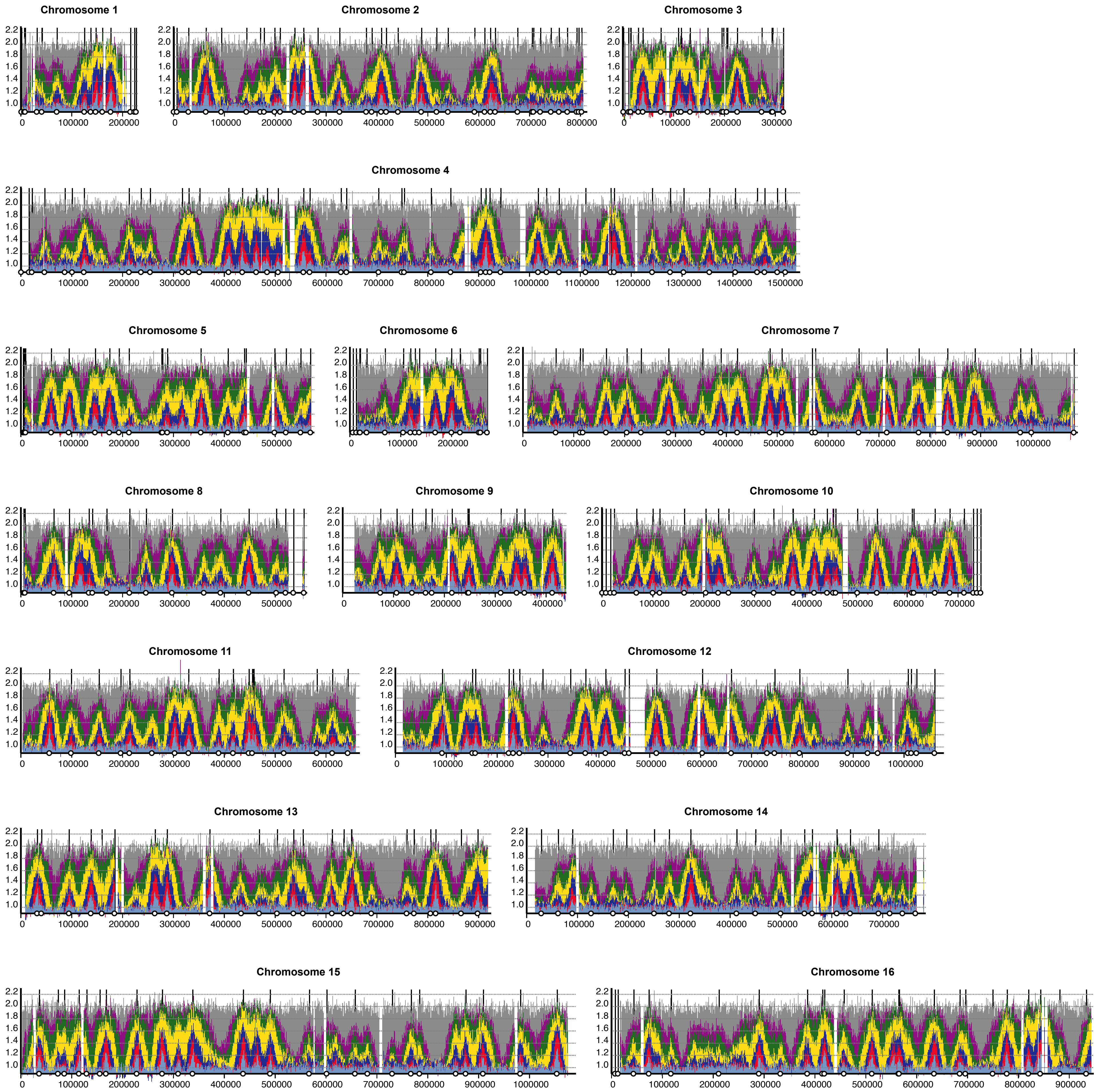
Chromosome 1
Chromosome 2
Chromosome 3
Chromosome 4
Chromosome 5
Chromosome 6
Chromosome 7
Chromosome 8
Chromosome 9
Chromosome 10
Chromosome 11
Chromosome 12
Chromosome 13
Chromosome 14
Chromosome 15
Chromosome 16

Müller, Hawkins, et al. Figure S5

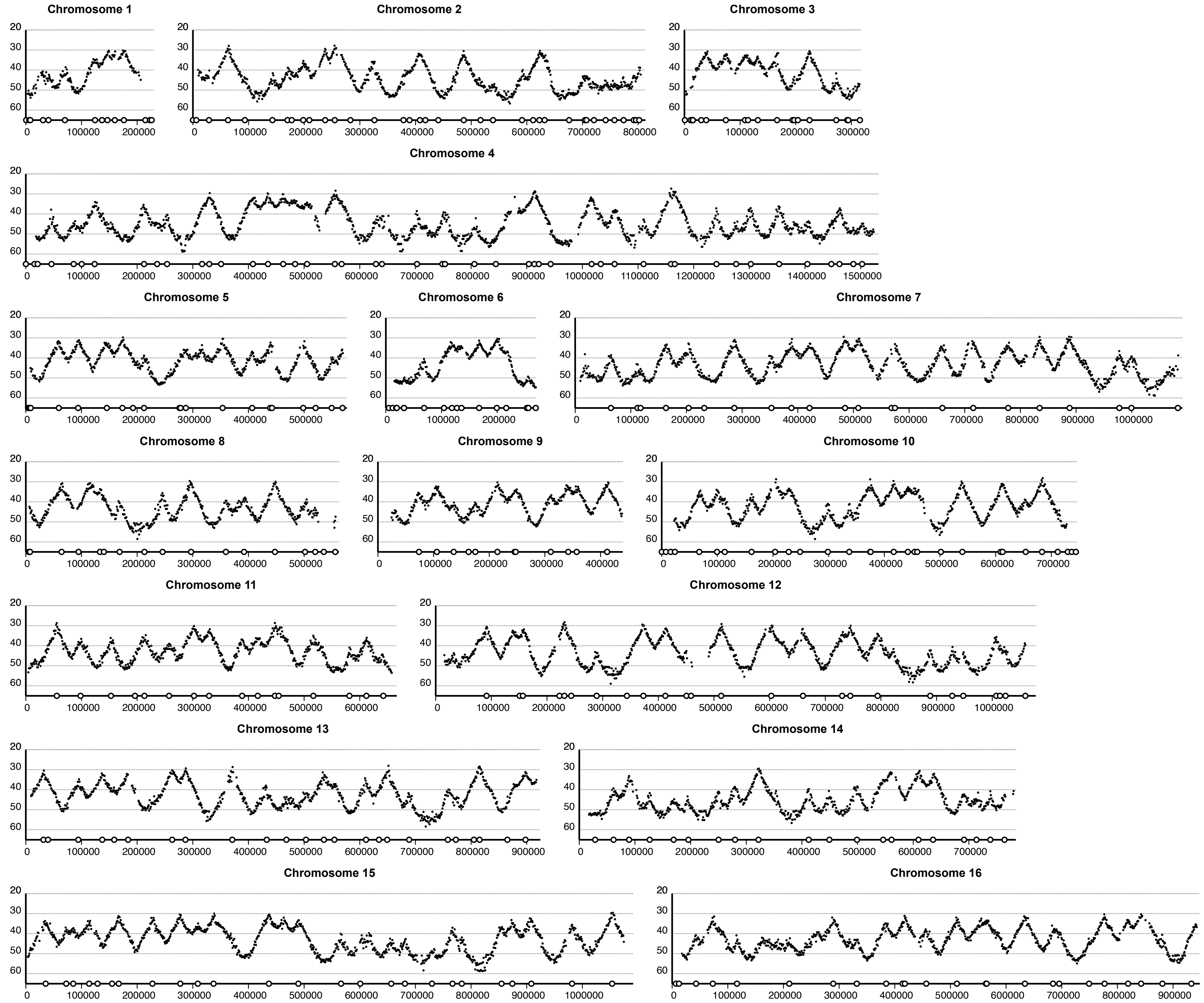

Müller, Hawkins, et al. Figure S6

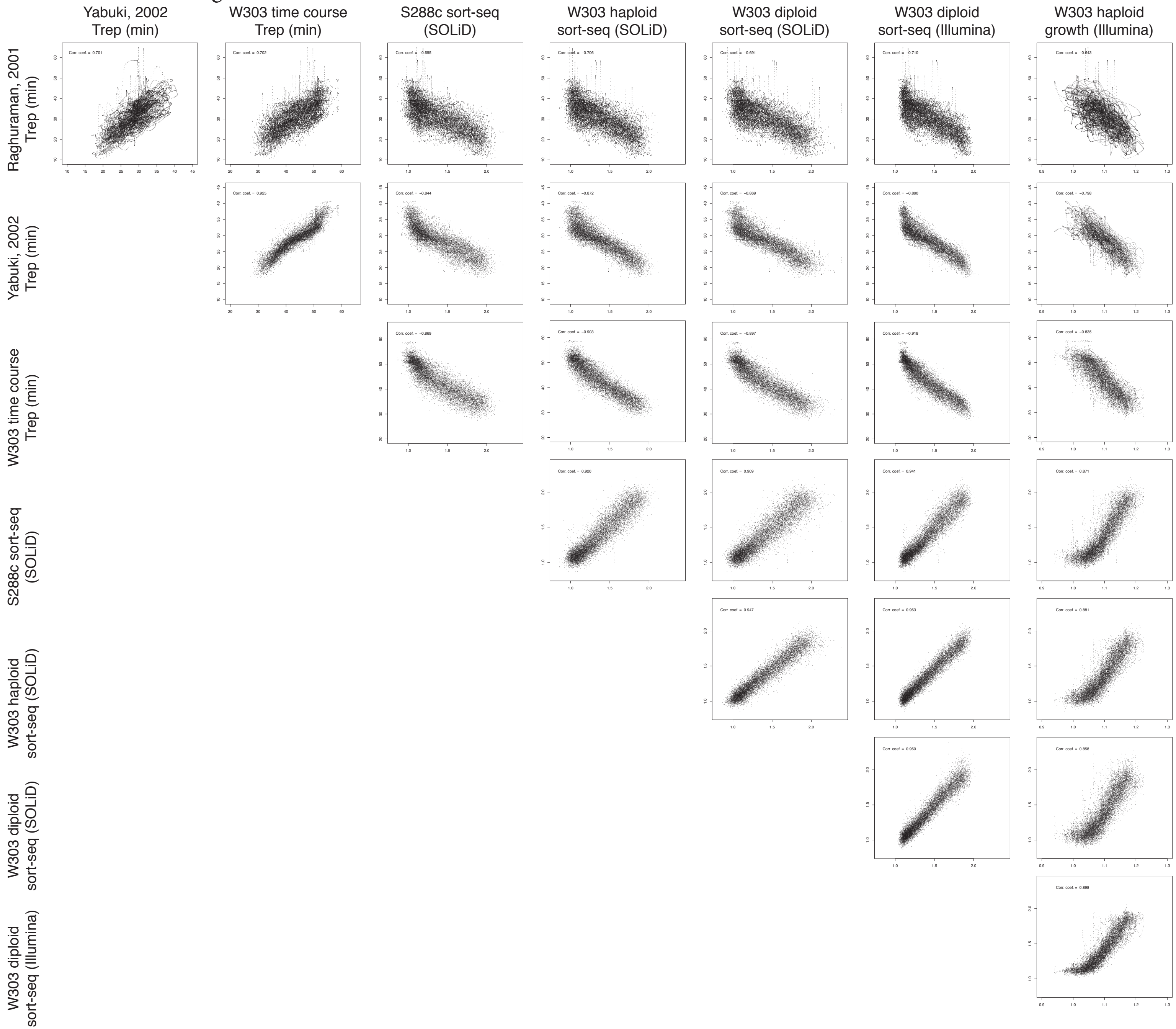

Müller, Hawkins, et al. Figure S7

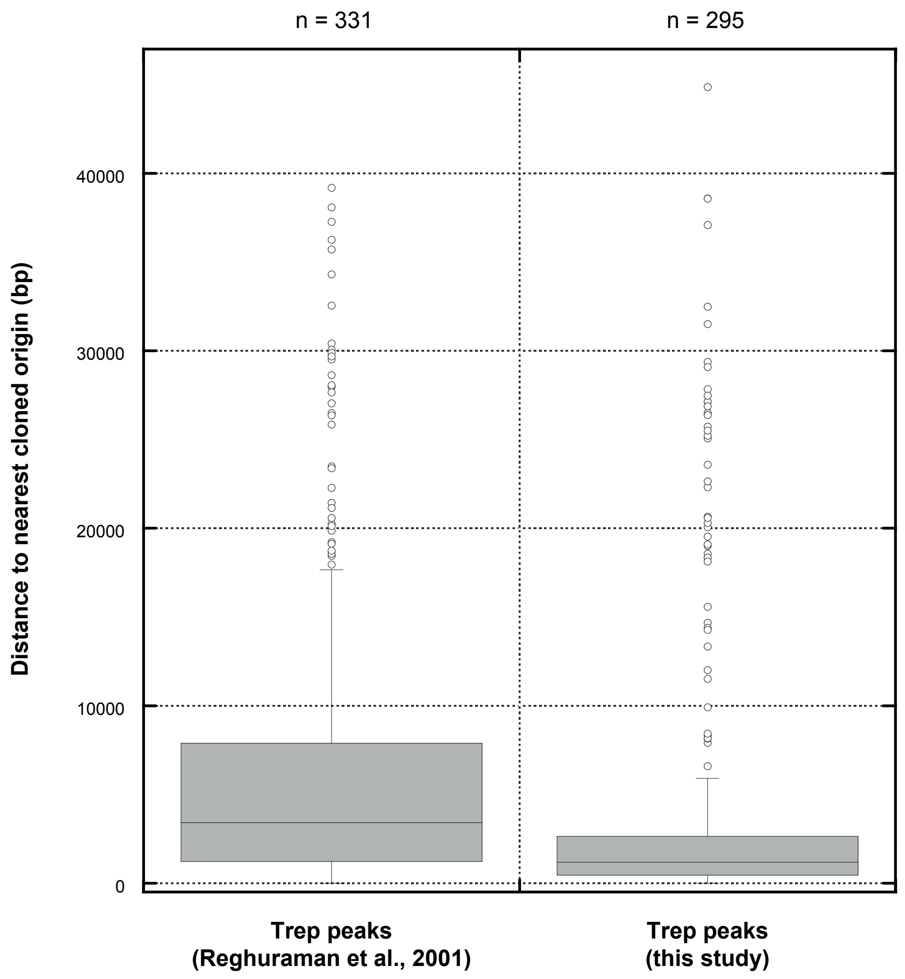

Müller, Hawkins, et al. Figure S8

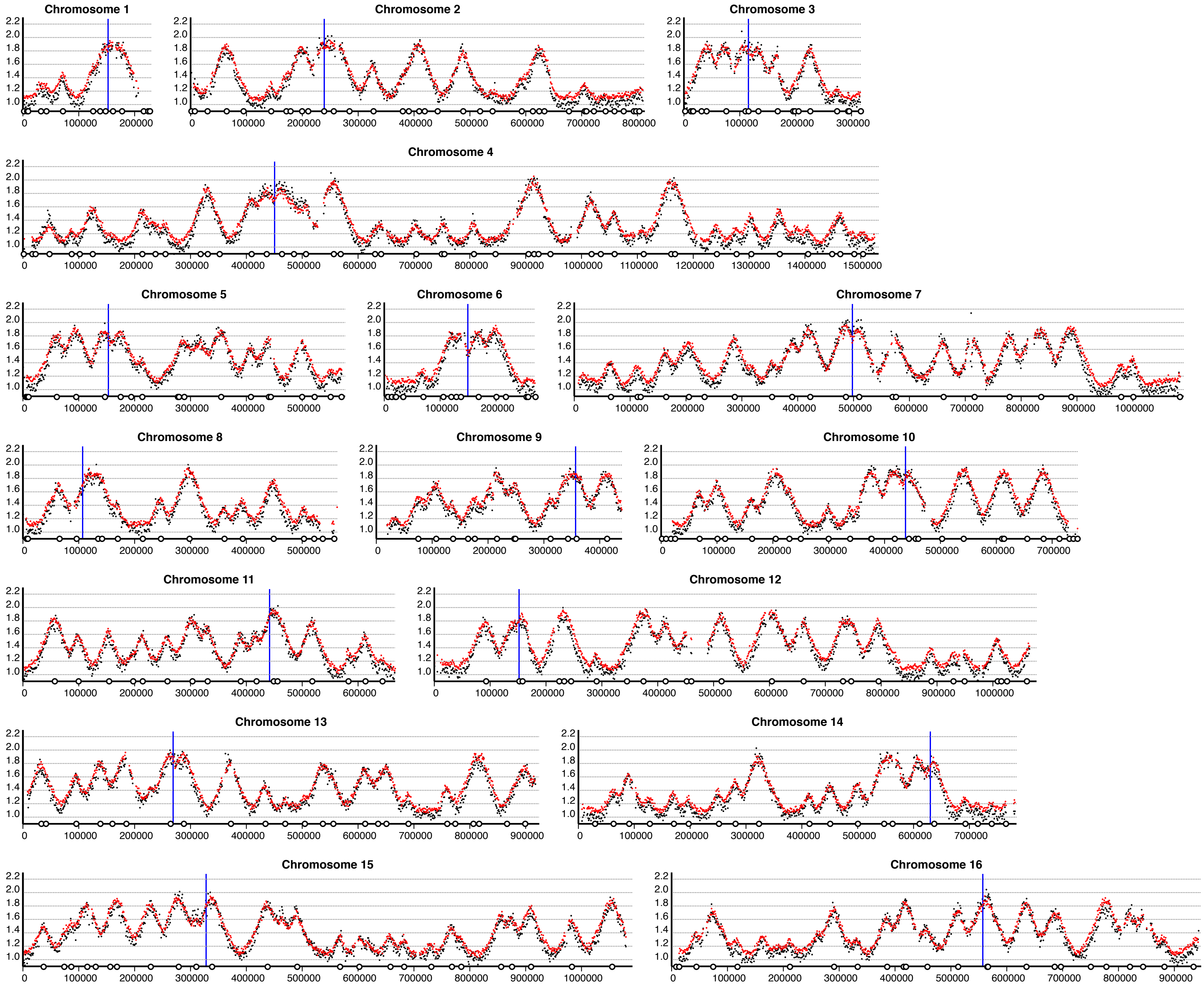

Müller, Hawkins, et al. Figure S9

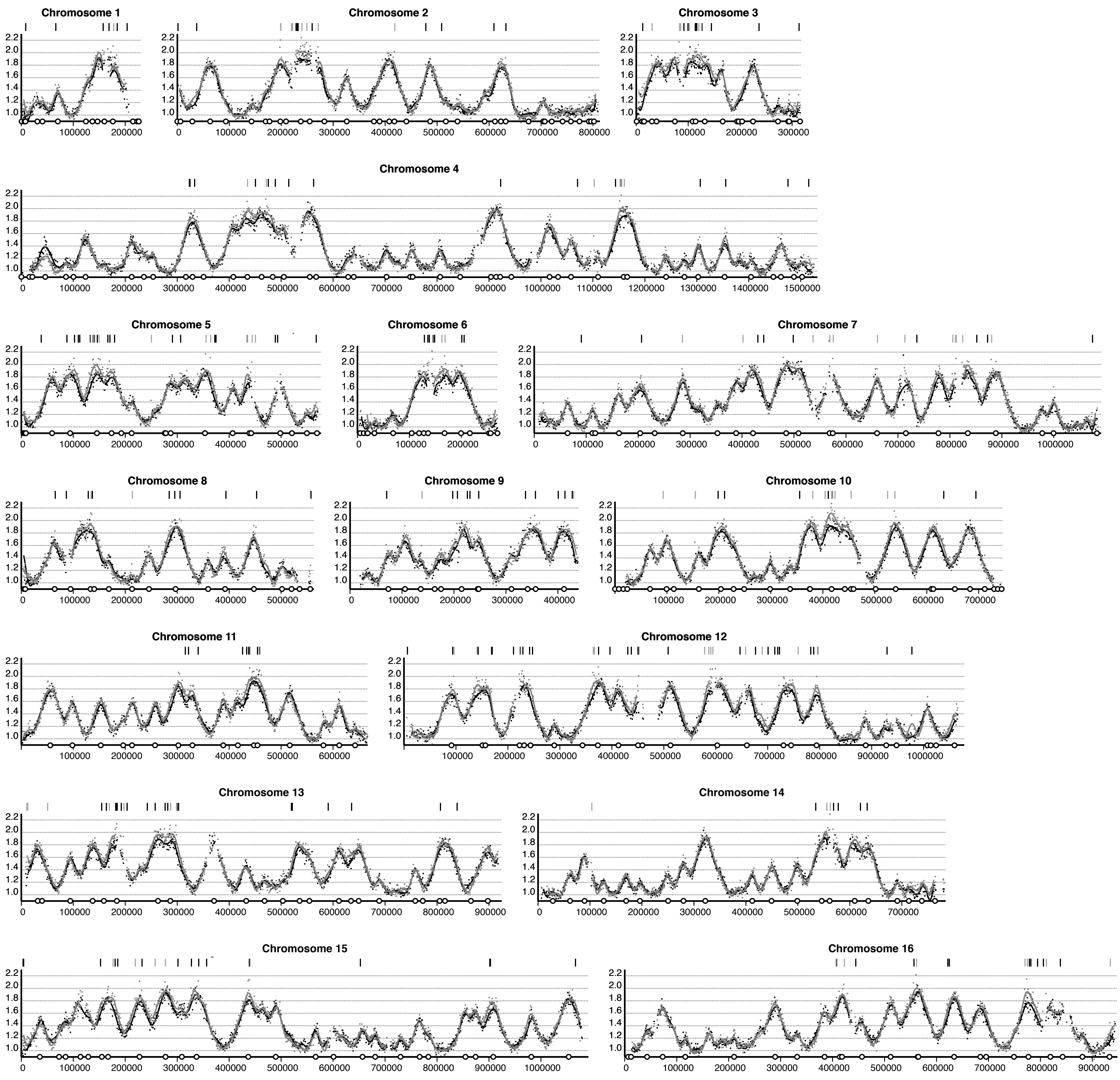

Müller, Hawkins, et al. Figure S10

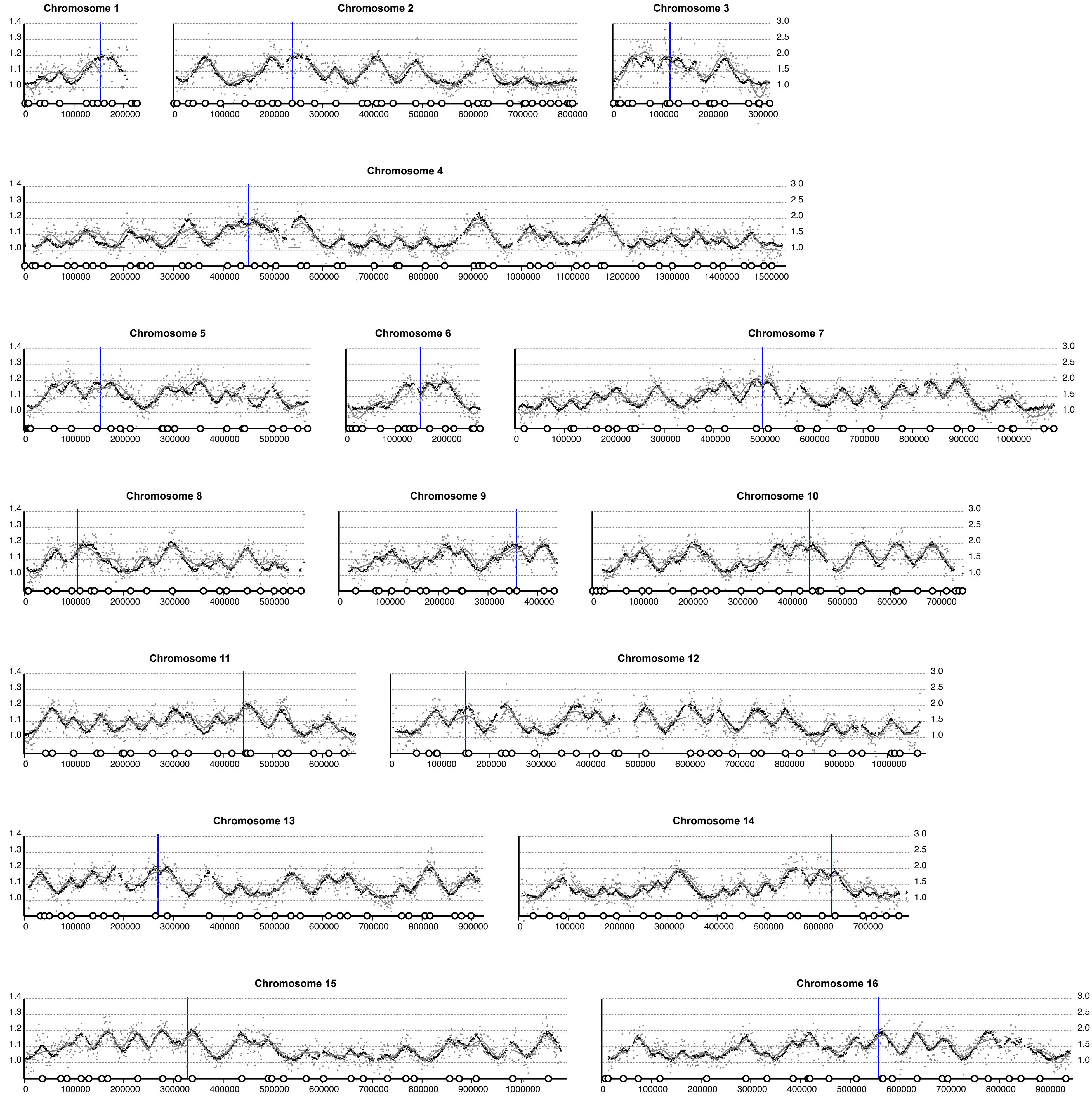

Technology & sequencing platform	Strain & Selected Genotype	Library construction	Sample	Mean Copy Number by Flow Cytometry	Total Reads	Uniquely Mapping Reads
**SOLiD** 5500 system	AUY077 MATa	5500 SOLiD Fragment library core kit (Applied biosystems, 4464412)	Alpha factor arrest	1.00	17,679,718	16,056,228
			25 min release	1.06	23,564,200	20,997,274
			30 min release	1.09	24,993,881	22,501,754
			35 min release	1.21	26,283,891	23,893,069
			40 min release	1.40	27,809,830	25,386,748
			45 min release	1.56	23,736,925	21,968,165
			50 min release	1.68	22,005,344	20,535,997
			90 min release	1.95	32,053,518	29,619,880
**SOLiD** 3 plus system	CAY106 *MATa rad53Δ sml1Δ*	SOLiD Fragment Library Construction Kit (Applied biosystems, 4443471	Alpha factor arrest	N/A	19,075,049	18,675,416
			60 min in HU	N/A	13,712,377	13,317,219
**SOLiD** 4 system	T7107 Haploid	SOLiD Fragment Library Construction Kit (Applied biosystems, 4443471)	G2 phase sorted cells	N/A	21,461,618	20,442,400
			S phase sorted cells	N/A	16,935,636	16,205,005
**SOLiD** 4 system	T9475 Diploid	SOLiD Fragment Library Construction Kit (Applied biosystems, 4443471)	G2 phase sorted cells	N/A	45,368,930	43,734,400
			S phase sorted cells	N/A	24,346,946	23,183,066
**Illumina** HiSeq2000	T9475 Diploid	NEBNext DNA Library Prep Master Mix Set for Illumina	G2 phase sorted cells	N/A	164,698,046	131,816,351
			S phase sorted cells	N/A	195,717,543	155,734,122
**Illumina** HiSeq2000	T7107 Haploid	NEBNext DNA Library Prep Master Mix Set for Illumina	Stationary phase cells	N/A	293,409,051	234,532,691
			Exponential phase cells	N/A	262,092,475	213,008,532

Müller, Hawkins et al. (2013) Supplementary Table S1

Strain name	Genotype	Source
**AUY077**	ade2-1 trp1-1 can1-100 leu2-3,112 his3-11,15 ura3 GAL+ ssd1,d2 RAD5+	this study
**CAY106**	MATa; ade2-1; trp1-1; can1-100; leu2,3-112; his3-11,15; ura3; ssd1: bar1::TRP1; sml1::URA3 rad53::LEU2	this study
**T7107**	MATa, RAD5, BUD4, leu2, ura3, trp1, ade2, his3	Tomoyuki Tanaka
**T9475**	MatA/MATalpha, RAD5/RAD5, BUD4/BUD4, leu2/leu2, ura3/ura3, trp1/trp1, ade2/ade2, his3/his3	Tomoyuki Tanaka

Origin name	ACS sequence	Reference
**ARS202**	TTTTAATTTTT	This study
**ARS209**	TTTTATGTTTT	(Bouton and Smith)
**ARS214**	ATTTATATTTT	(Hoggard *et al.*)
**ARS219.5**	TTCTATATTTT	(Hoggard *et al.*)
**ARS224**	TTTAACGTTTT	This study
**ARS301**	TTTTATGTTTT	(Sharma *et al.*)
**ARS302**	TTTTATATTTT	(Sharma *et al.*)
**ARS303**	ATTTATATTTT	(Vujcic *et al.*)
**ARS304**	TTATAAATTT	(Bouton and Smith 1986)
**ARS305**	TTATATGTTTT	(Huang and Kowalski)
**ARS306**	ATTTATATTTA	(Theis *et al.*)
**ARS307**	ATTTATGTTTT	(Palzkill and Newlon)
**ARS309**	GTTTATATCTT	(Theis and Newlon)
**ARS310**	TTTTACTTTTT	(Theis and Newlon)
**ARS313**	TTTTACTTTTA	(Chang *et al.*)
**ARS315**	TTTTATGTTTT	(Crampton *et al.*)
**ARS316**	TTTCAAATTTA	(Chang *et al.*)
**ARS317**	TTTTATATTTA	(Chang *et al.*)
**ARS318**	TATCATGTTTT	(Chang *et al.*)
**ARS319**	TTTTATGTTTA	(Chang *et al.*)
**ARS320**	TTTTATGTTAT	(Vujcic *et al.*)
**ARS404**	TTTAATATTTT	(Kearsey)
**ARS416**	TTTTATGTTTA	(Celniker *et al.*)
**ARS422**	ATTAATGTTTT	This study
**ARS423**	TTTTACATTTT	(Hoggard *et al.*)
**ARS428**	TTTTATATTTT	(Hoggard *et al.*)
**ARS432**	TTTTTTTTCTTTTCT	(Nieduszynski *et al.*)
**ARS432.5**	TTATTTACATTTTGT	(Nieduszynski *et al.*)
**ARS442**	ATTTATGTTTA	(Hoggard *et al.*)
**ARS512**	AATTATGTTTA	(Hoggard *et al.*)

**ARS514**	ATTTATGTTTT	(Hoggard *et al.*)
**ARS516**	ATTTACTTTTT	(Hoggard *et al.*)
**ARS601**	ATTTCCATTTT	(Shirahige *et al.*)
**ARS602**	TTATACGTTTA	(Shirahige *et al.*)
**ARS603**	TTTAAAGTTTT	(Shirahige *et al.*)
**ARS603**	TTTCATATTTT	(Shirahige *et al.*)
**ARS603.5**	TTCCATATTTT	(Yamashita *et al.*)
**ARS604**	TTTTACGTTTT	(Shirahige *et al.*)
**ARS605**	AATTACGTTTT	(Shirahige *et al.*)
**ARS606**	ATTTATATTTT	(Shirahige *et al.*)
**ARS607**	GTTTATATTTA	(Shirahige *et al.*)
**ARS608**	TTTTACTTTTA	(Shirahige *et al.*)
**ARS609**	TTTTATGTTTT	(Shirahige *et al.*)
**ARS702**	TTTTTAATATTTTGT	(Nieduszynski *et al.*)
**ARS704**	TTTTATACGTTTATG	(Nieduszynski *et al.*)
**ARS707**	CATTTTATAATTTGT	(Nieduszynski *et al.*)
**ARS710**	TTTTTTATATTTATT	(Nieduszynski *et al.*)
**ARS714**	TTATTTACTTTTAGT	(Nieduszynski *et al.*)
**ARS716**	AATTACGTTTA	(Hoggard *et al.*)
**ARS717**	TTATTTAACTTTTGT	(Nieduszynski *et al.*)
**ARS718**	AAATTATTGTTTAGT	(Nieduszynski *et al.*)
**ARS719**	TTATTTATGTTTTGC	(Nieduszynski *et al.*)
**ARS721**	GTATTTATATTTAGC	(Nieduszynski *et al.*)
**ARS727**	TATTTTATGTTTACT	(Nieduszynski *et al.*)
**ARS728**	TTGTTTATATTTTGT	(Nieduszynski *et al.*)
**ARS729**	TTTTTTACCTTTTGT	(Nieduszynski *et al.*)
**ARS731**	TGTATATAGTTTAGT	(Nieduszynski *et al.*)
**ARS731.5**	ATTTTAATATTTTGT	(Nieduszynski *et al.*)
**ARS733**	TTTTTTAATTTTTTT	(Nieduszynski *et al.*)
**ARS809**	TTAGACATTTA	(Hoggard *et al.*)
**ARS818**	TTTCATGTTTT	(Hoggard *et al.*)
**ARS822**	TTATACATTTT	(Hoggard *et al.*)
**ARS911**	GTTTATGTTTT	(Hoggard *et al.*)

**ARS920**	TTTTATATTTT	(Hoggard *et al.*)
**ARS1001**	TTTTATGTTTA	(Xu *et al.*)
**ARS1002**	TTTTATGTTTA	(Xu *et al.*)
**ARS1003**	TTTTATTTTTA	(Xu *et al.*)
**ARS1004**	TTTTTAGTTTT	(Xu *et al.*)
**ARS1005**	TTATATGTTTT	(Xu *et al.*)
**ARS1007**	ATATATATTTA	(Xu *et al.*)
**ARS1007.5**	ATCTATGTTTA	(Xu *et al.*)
**ARS1009**	ATTTATATTTA	(Xu *et al.*)
**ARS1011**	TTTTATGTTTA	(Xu *et al.*)
**ARS1014**	TTTTATATTTA	(Xu *et al.*)
**ARS1015**	ATTTATATTTT	(Xu *et al.*)
**ARS1018**	TTTTACATTTA	(Xu *et al.*)
**ARS1019**	TTTTATCTTTA	(Xu *et al.*)
**ARS1020**	ATTTACATTTT	(Xu *et al.*)
**ARS1021**	TTGTTTAACATTAGT	(Nieduszynski *et al.*)
**ARS105**	ATTAACAATTA	(Hoggard *et al.*)
**ARS1114**	TTTTATGTTTT	(Hoggard *et al.*)
**ARS1118**	TTTTACATTTA	This study
**ARS1123**	TTTTATATTTA	(Hoggard *et al.*)
**ARS1216.5**	GTTTATGTTTT	(Nieduszynski *et al.*)
**ARS1307**	ATTTATGTTTT	(Hoggard *et al.*)
**ARS1320**	ATTTATATTTA	(Hoggard *et al.*)
**ARS1323**	GTTTATGTTTA	(Hoggard *et al.*)
**ARS1324**	TTTTACTATTT	This study
**ARS1325**	TTTCATATTTT	(Hoggard *et al.*)
**ARS1329**	ATTTAGTCTTT	(Hoggard *et al.*)
**ARS1332**	TTTTATGTTTG	(Hoggard *et al.*)
**ARS1405**	TTTTATTTTTA	(Hoggard *et al.*)
**ARS1413**	ATTTGTATTTA	(Friedman *et al.*)
**ARS1420**	CTTTATGTTTA	(Hoggard *et al.*)
**ARS1513**	TTTTACCTTTT	(Hoggard *et al.*)
**ARS1521**	TTTTATATTTT	(Hoggard *et al.*)

**ARS1526**	ATTTTAATATTTGTT	(Nieduszynski *et al.*)
**ARS1528**	TTTGTTTATGTTTAGGT	(Breier *et al.*)
**ARS1528**	GTTTATGTTTA	(Hoggard *et al.*)
**ARS1529.5**	TTGTTTAAATTTTGT	(Nieduszynski *et al.*)
**ARS1529.5**	GTTTAAATTTT	(Hoggard *et al.*)
**ARS1625**	ATTTACGTTTA	(Hoggard *et al.*)
**ARS1626.5**	TTATTTATATTTTGG	(Nieduszynski *et al.*)
**ARS1631**	TTTTATATTTG	(Hoggard *et al.*)

Bouton AH, Smith MM. 1986. Fine-structure analysis of the DNA sequence requirements for autonomous replication of Saccharomyces cerevisiae plasmids. *Molecular and cellular biology* **6**(7): 2354-2363.

Breier AM, Chatterji S, Cozzarelli NR. 2004. Prediction of Saccharomyces cerevisiae replication origins. *Genome Biol* **5**(4): R22.

Celniker SE, Sweder K, Srienc F, Bailey JE, Campbell JL. 1984. Deletion mutations affecting autonomously replicating sequence ARS1 of Saccharomyces cerevisiae. *Molecular and cellular biology* **4**(11): 2455-2466.

Chang F, Theis JF, Miller J, Nieduszynski CA, Newlon CS, Weinreich M. 2008. Analysis of chromosome III replicators reveals an unusual structure for the ARS318 silencer origin and a conserved WTW sequence within the origin recognition complex binding site. *Mol Cell Biol* **28**(16): 5071-5081.

Crampton A, Chang F, Pappas DL, Frisch RL, Weinreich M. 2008. An ARS element inhibits DNA replication through a SIR2-dependent mechanism. *Mol Cell* **30**(2): 156-166.

Friedman KL, Diller JD, Ferguson BM, Nyland SV, Brewer BJ, Fangman WL. 1996. Multiple determinants controlling activation of yeast replication origins late in S phase. *Genes Dev* **10**(13): 1595-1607.

Hoggard T, Shor E, Müller CA, Nieduszynski CA, Fox CA. 2013. Linking ORC-Origin Binding Mechanisms to the Regulation of Origin Activation Time. *PLoS Genet, in press.*

Huang RY, Kowalski D. 1996. Multiple DNA elements in ARS305 determine replication origin activity in a yeast chromosome. *Nucleic Acids Res* **24**(5): 816-823.

Kearsey S. 1984. Structural requirements for the function of a yeast chromosomal replicator. *Cell* **37**(1): 299-307.

Nieduszynski CA, Knox Y, Donaldson AD. 2006. Genome-wide identification of replication origins in yeast by comparative genomics. *Genes Dev* **20**(14): 1874-1879.

Palzkill TG, Newlon CS. 1988. A yeast replication origin consists of multiple copies of a small conserved sequence. *Cell* **53**(3): 441-450.

Sharma K, Weinberger M, Huberman JA. 2001. Roles for internal and flanking sequences in regulating the activity of mating-type-silencer-associated replication origins in Saccharomyces cerevisiae. *Genetics* **159**(1): 35-45.

Shirahige K, Iwasaki T, Rashid MB, Ogasawara N, Yoshikawa H. 1993. Location and characterization of autonomously replicating sequences from chromosome VI of Saccharomyces cerevisiae. *Mol Cell Biol* **13**: 5043–5056.

Theis JF, Newlon CS. 1997. The ARS309 chromosomal replicator of Saccharomyces cerevisiae depends on an exceptional ARS consensus sequence. *Proc Natl Acad Sci U S A* **94**(20): 10786-10791.

Theis JF, Newlon CS. 2001. Two compound replication origins in Saccharomyces cerevisiae contain redundant origin recognition complex binding sites. *Mol Cell Biol* **21**(8): 2790-2801.

Theis JF, Yang C, Schaefer CB, Newlon CS. 1999. DNA sequence and functional analysis of homologous ARS elements of Saccharomyces cerevisiae and S. carlsbergensis. *Genetics* **152**(3): 943-952.

Vujcic M, Miller CA, Kowalski D. 1999. Activation of silent replication origins at autonomously replicating sequence elements near the HML locus in budding yeast. *Mol Cell Biol* **19**(9): 6098-6109.

Xu W, Aparicio JG, Aparicio OM, Tavaré S. 2006. Genome-wide mapping of ORC and Mcm2p binding sites on tiling arrays and identification of essential ARS consensus sequences in S. cerevisiae. *BMC Genomics* **7**: 276.

Yamashita M, Hori Y, Shinomiya T, Obuse C, Tsurimoto T, Yoshikawa H, Shirahige K. 1997. The efficiency and timing of initiation of replication of multiple replicons of Saccharomyces cerevisiae chromosome VI. *Genes to Cells* **2**: 655-666.
